# Extreme Dry‐Heat Climate Impacts on Greenhouse Gas Emission Intensity in Wheat Production: Insights and Mitigation Strategies

**DOI:** 10.1111/gcb.70349

**Published:** 2025-07-11

**Authors:** Yu Shi, Shufen Pan, Yongfa You, Stephen A. Prior, Di Tian, Huiqian Yu, Qiang Yu, Hanqin Tian

**Affiliations:** ^1^ Institute of Carbon Neutrality, Sino‐French Institute for Earth System Science, College of Urban and Environmental Sciences Peking University Beijing China; ^2^ Center for Earth System Science and Global Sustainability, Schiller Institute for Integrated Science and Society Boston College Chestnut Hill Massachusetts USA; ^3^ Department of Engineering and Environmental Studies Program Boston College Chestnut Hill Massachusetts USA; ^4^ USDA‐ARS National Soil Dynamics Laboratory Auburn Alabama USA; ^5^ Department of Crop, Soil, and Environmental Sciences, College of Agriculture Auburn University Auburn Alabama USA; ^6^ State Key Laboratory of Soil Erosion and Dryland Farming on the Loess Plateau Institute of Soil and Water Conservation, Northwest A&F University Yangling China; ^7^ Department of Earth and Environmental Sciences Boston College Chestnut Hill Massachusetts USA

**Keywords:** crop production, DLEM (dynamic land ecosystem model), dry‐heat climate, greenhouse gas emission intensity, tillage effects, wheat

## Abstract

Extreme dry‐heat (EDH) climate poses significant challenges to global food production and exacerbates greenhouse gas (GHG) emissions, impeding efforts to mitigate agricultural climate impacts. However, the concurrent effects of long‐term EDH climate and mitigation strategies on cropland productivity and GHG emissions remain poorly understood. Here, we integrated field observations, agroecosystem model outputs, and nursery data to examine how environmental factors and management practices influence wheat GHG emission intensity across the U.S. over the past six decades. Our findings indicate an overall increase in U.S. wheat production over the past 60 years, despite fluctuations in planted areas that have led to declines in production after 1990. The decline in GHG emissions from winter wheat after 1990 corresponds to fluctuations in planting areas, whereas emissions from spring wheat have continued to rise. Climate change and nitrogen fertilizer application have emerged as the primary drivers of these trends. EDH climates have intensified emissions intensity in over 80% of wheat‐growing regions under current agricultural management practices. Specifically, the dry‐heat sensitivity of emission intensity for spring wheat increased by 130% from 1960 to 2018, while for winter wheat, it surged several‐fold after 2008. To address these challenges, we propose environment‐specific tillage strategies to significantly reduce the dry‐heat sensitivity of GHG emission intensity under local conditions. These strategies identify regionally optimal tillage schemes (including no‐tillage and conventional tillage) to mitigate the adverse impacts of EDH climates. The implementation of these strategies in selected wheat‐producing regions reduced dry‐heat sensitivity by 9.8% (5.8%–17.7%) for spring wheat and 13.3% (8.0%–20.9%) for winter wheat emissions intensity. These findings underscore the critical need for targeted management approaches to alleviate the escalating indirect impacts of EDH climates. Such strategies are crucial for shaping agricultural and environmental policies aimed at achieving high‐yield and low‐emission targets in a warming world.

## Graphical abstract

Extreme dry‐heat events increasingly threaten crop yields and intensify greenhouse gas emissions from agriculture, yet their long‐term impacts and mitigation potential remain poorly understood. Using field data, models, and nursery experiments across the U.S., we show that dry‐heat climates have significantly increased the greenhouse gas emission intensity of wheat production, with sensitivity rising several‐fold over the past six decades. Region‐specific tillage strategies that reduce dry‐heat sensitivity by up to 13% point to the urgent need for climate‐adaptive management to maintain yields and lower emissions as heat extremes intensify.

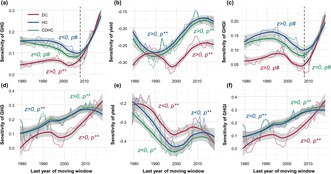

## 1 Introduction

Amid global climate change, the world's major grain‐producing regions are experiencing varying degrees of aridification and warming, accompanied by a series of extreme dry‐heat (EDH) events. EDH conditions threaten agricultural production by depleting available water (Jin et al. 2023), limiting plant physiological processes (Nicolas et al. 1984; Lobell et al. 2012), and shortening crop growth periods (Nicolas et al. 1984; Asseng et al. 2015). These conditions triggered heat and drought stress, further reducing the carbon fixation capacity in crops (Lobell et al. 2012) and enhancing autotrophic and heterotrophic respiration (Bardgett et al. 2008; O'Connell et al. 2018; Harris et al. 2022). As a result, they cause sharp increases in agricultural greenhouse gas (GHG) emissions and the climate costs of food production. Non‐linear and intensified adverse effects of extreme climates on crop yields have been observed in previous studies conducted in warming environments (Schlenker and Roberts 2009; Hogan and Schlenker 2024). However, the intricate response of cropland GHG emissions to long‐term exposure to EDH climates remains largely understudied, particularly regarding its interaction with key biophysical factors such as soil properties and management practices (Dong et al. 2025). This significantly hinders our understanding of carbon and nitrogen cycling in agricultural ecosystems and limits accurate assessment of GHG budgets.

Agricultural tillage practices offer a potential solution for mitigating the adverse effects of climate change (Gilbert 2011; Lipper et al. 2014). Conventional tillage, characterized by plowing and soil disturbance, is the most widely adopted tillage practice and has been shown to significantly improve crop productivity and resilience to environmental stresses (Pittelkow, Liang, et al. 2015; Cui et al. 2024). As concerns regarding soil degradation and GHG emissions increase, sustainable tillage designs are increasingly advocated for local agricultural production and emission reduction due to their potential benefits in sustainability, cost‐effectiveness, and ease of implementation (Gan et al. 2014; Jat et al. 2020). These practices include no‐tillage (NT), reduced tillage, and conservation tillage. Nevertheless, farmers must balance the need for production with the imperative to reduce environmental burdens, as these objectives are often in conflict within the context of individual tillage practices. Previous studies have often reported inconsistent or even contradictory findings. This is likely because the tillage effects are highly dependent on site‐specific factors such as soil characteristics, local climate, and biodiversity. For example, plow tillage has been found to improve soil aeration and the transport of NO_3_^−^, NH_4_^+^, and organic carbon to the soil surface, thereby promoting carbon dioxide (CO_2_), nitrous oxide (N_2_O), and NO emissions (Carbonell‐Bojollo et al. 2022; Li et al. 2023; Guo et al. 2024). In contrast, other studies suggest that NT increases denitrification rates and microbial biomass in the upper soil layers, potentially contributing to soil CO_2_ and N_2_O emissions (Jantalia et al. 2008; Mkhabela et al. 2008; Lognoul et al. 2017). Additionally, some studies have reported reduced crop productivity under NT (Pittelkow, Linquist, et al. 2015; Cui et al. 2024), while others suggest that plow tillage may harm crop yields by disrupting the soil‐aggregate structure and altering soil water and gas dynamics (Guo et al. 2024). In summary, the complexity of underlying surface conditions—particularly in the context of increasing EDH events—presents challenges to the effectiveness, viability, and sustainability of tillage schemes. Optimizing trade‐offs in tillage practices is therefore crucial for balancing productivity and emissions at the levels of farmers, regions, and nations.

The United States (US) is a major wheat producer and exporter, playing a critical role in global food security and significantly influencing food prices through its impact on the global supply chain and trade. However, severe aridity and heatwaves have increasingly impacted US wheat production over the past four decades (Zhu and Burney 2021; Zhao et al. 2022), resulting in yield reductions of 10%–40% (Lollato et al. 2017; Zhao et al. 2022) and potential GHG emissions associated with winter wheat production (Smith et al. 2013; Ghimire et al. 2019). In the U.S. Great Plains, EDH climates have led to annual yield losses of up to 9 kg ha^−1^ in severely affected counties (Zhao et al. 2022), equivalent to approximately ~1.9 million tons of winter wheat production. The interactions between climate extremes and food production in this region facilitate the investigation of how climate costs respond to EDH climates within the context of food production, the dynamics in their sensitivity, and the mitigating effects of environment‐specific tillage practices.

Given the increasing frequency of EDH events linked to climate change, agricultural sustainable development confronts dual challenges: ensuring food production to safeguard livelihoods, while minimizing the environmental impacts. Mitigating GHG emission intensity (GHGI, the amount of GHG emissions per unit of food production) through tillage management is therefore critical to achieving both food security and cleaner production. For this, we employed a model‐data integration framework that couples a process‐based terrestrial biosphere model [Dynamic Land Ecosystem Model (DLEM)] with multiple in situ measurements and nursery yield statistics to investigate the sensitivity and dynamics of US wheat yield (spring and winter wheat), net GHG emissions [CO_2_, N_2_O, and methane (CH_4_)], and GHGI under EDH climates, including both individual and compound events. Additionally, two tillage scenarios were simulated to examine the feasibility and sustainability of implementing environment‐specific tillage practices to mitigate the adverse effects of EDH climates on GHGI. Our study aimed to: (i) quantify the magnitude, trends, and spatiotemporal variations of wheat yield, net GHG emissions, and GHGI in the US from 1960 to 2018; (ii) attribute variations in yield and GHGs to natural and anthropogenic management factors (as detailed in Table S1); (iii) analyze the temporal variations in wheat GHGI sensitivity to EDH climates; and (iv) evaluate the effects of context‐specific tillage practices—NT and conventional tillage (CT)—in mitigating sensitivity to EDH climates and assess the potential of environment‐specific tillage strategies. The findings of this study will provide insights into the systematic changes in wheat sensitivity to EDH climates under long‐term warming. Furthermore, this research proposes climate‐dependent agricultural tillage strategies to mitigate the adverse impacts of extreme climate conditions, offering a valuable foundation for policymakers to develop effective agricultural and environmental policies aimed at achieving high‐yield and low‐emission pathways under a warming climate.

## 2 Materials and Methods

### 2.1 Model Description

#### 2.1.1 Overview

The new agricultural module of the DLEM v4.0 model (You et al. 2022) is developed based on previous versions (DLEM‐Ag, Ren et al. 2012; and DLEM‐Ag2, Zhang et al. 2018) and includes significant advancements in crop growth processes and key management practices (e.g., fertilization, irrigation, tillage, and cover cropping). For wheat, the new model significantly improves regional simulation performance and the capacity to quantify the impacts of agricultural activities on biosphere‐atmosphere interactions. These improvements are achieved through better representation of crop phenological development, dynamic carbon allocation, yield formation, and management practices. Specifically, we first introduced phenological development frameworks for winter and spring wheat, accounting for the environmental stresses associated with wheat growth. Second, a new scheme for dynamic carbon allocation was implemented (i.e., the allocation ratio of net assimilates was determined by the carbon allocation curve in different vegetation pools and adjusted based on water, light, and nitrogen stress). Third, yield formation was calculated as the balance between the carbon supply in the reproductive pool and the carbon demand for grain filling. Finally, we incorporated essential management practices into DLEM v4.0, such as tillage, cover cropping, and crop genetic improvements, and implemented a dynamic rotation scheme using real‐time rotation maps to reflect annual changes in crop distribution and types.

In DLEM v4.0, net CO_2_ emissions are derived from soil carbon pool turnover, regulated by vegetation photosynthesis, litter accumulation, autotrophic and heterotrophic respiration, and environmental changes. CH_4_ exchange is the balance between production, oxidation, and transportation from the soil pore water spaces to the atmosphere. The N_2_O module captures nitrification and denitrification processes and considers relevant factors such as nitrogen availability, soil characteristics, and thermal and moisture statuses. Previous studies have detailed the model's mechanisms for yield formation, GHG emission, the interaction between carbon dynamics and environmental stress, and the response of agricultural carbon cycling to anthropogenic management practices (Tian, Xu, et al. 2010; Ren et al. 2012; Lu et al. 2022; You et al. 2022). Notably, the definitions and units of all terminology used in this study are listed in Table S6.

#### 2.1.2 Wheat Phenological Development and Yield Formation in DLEM v4.0

(1) Wheat phenological development: The new model explicitly considers phenological differences between wheat and other crops, as well as phenological stage‐dependent environmental stresses. Specifically, DLEM v4.0 follows a phenological development cycle similar to that of DLEM‐Ag2 (Ren et al. 2012; Zhang et al. 2018) but incorporates more detailed phenological stages and considers the effects of environmental stresses, such as water and nitrogen, on phenological development (Figure S1). Additionally, the model divided the wheat life cycle into ten stages: sowing, germination, emergence, terminal spikelets, end of leaf growth, end of ear growth, beginning of grain filling, end of grain filling, physiological maturity, and harvest (Figure S1). If the number of days after the simulated sowing date exceeded a phenology‐specific threshold, seed germination was triggered, followed by subsequent phenological development. The beginning time and duration of specific phenological stages were determined according to the phenological development scheme, that is, Biological Days (BD) (Soltani and Sinclair 2012). Subsequently, the fraction of cumulative biological days (fCBD), which served as an indicator of growth rate, was calculated by dividing the actual accumulated BD from germination to the current day by the total BD required for maturity. The specific formulas for BD and fCBD, along with the target fCBD for each phenological stage, are detailed in Text S1 and S2 and You et al. (2022).

(2) Yield formation: In DLEM v4.0, yield formation follows a supply–demand relationship. Specifically, it is estimated as the balance between the available carbon assimilation supply in reproduction pools and the actual carbon demand for crop grain filling (Jones et al. 2003). Additionally, we considered crop‐specific grain‐filling characteristics to calculate the actual carbon demand for wheat (You et al. 2022). The new model also accounts for translocation of dry matter between stem tissues and the reproductive pool. If the available carbon assimilates fall short of meeting the actual carbon demand, the model allows the transfer of carbon from the stem pool to the reproduction pool to supplement grain filling, limited to a maximum of 20%. If excess assimilates were present, carbon exceeding the actual demand was transferred back from the reproduction pool to the stem pool to ensure mass balance.

#### 2.1.3 Improved Tillage and Other Management Practices

DLEM v4.0 incorporates four tillage practices (NT, conservation tillage, reduced tillage, and CT) based on tillage depth, mixing efficiency, and the proportion of soil surface covered by residues after tillage (You et al. 2022). The effects of tillage on the agricultural ecosystem are represented in three aspects: (1) changes in surface residue coverage due to tillage mixing, resulting in the redistribution of soil organic matter and nutrients; (2) changes in litter content, bulk density, moisture, and nitrogen cycling processes (e.g., nitrification, denitrification, and leaching); and (3) changes in soil decomposition rates (You et al. 2022). A comprehensive description of the effects of tillage practices on soil organic matter and nutrient contents, soil water processes, and decomposition is provided in Text S3. Additionally, relevant tillage parameters are listed in Table S1.

The new model also includes cover cropping, representing practices by planting crops (e.g., winter rye and peas) in the fallow period and leaving crop biomass in the field for the next crop‐growing season (Huang et al. 2020). We also incorporated simplified representations of genetic improvement strategies because the substantial increase in crop yields over the past decades can largely be attributed to advancements in management practices and crop breeding (Hammer et al. 2009; Pingali 2012). The effects of crop genetic improvements on the yield are achieved through two mechanisms: increasing the photosynthetic rate (maximum carboxylation) and enhancing the nitrogen uptake ability of wheat. Further details and parameters on wheat genetic improvements are provided in Lu et al. (2018) and You et al. (2022). In addition to these new considerations, DLEM v4.0 also enhances the representation of existing crop rotation practices by introducing time‐varying crop rotation schemes into the new model, rather than the static rotation maps used in previous versions.

### 2.2 Input Data

#### 2.2.1 Gridded Datasets

We developed long‐term spatial datasets for the continental US with 5 × 5 arcmin to drive DLEM v4.0, including atmospheric CO_2_ concentrations, nitrogen deposition, soil properties, land cover change, crop rotation, nitrogen fertilizer and manure applications, irrigation, tillage intensity, and phenological periods. These historical dynamic data, spanning from 1860 to 2018, served as fundamental elements driving the model. These data have been extensively used to estimate regional terrestrial carbon and nitrogen cycles and the budget for GHG emissions. Table S2 provides details of the sources, periods, temporal–spatial resolution, and processing methods for the driving data. This study provides detailed descriptions of wheat production simulation data, including wheat varieties, planting dates, rotations, fertilization, irrigation, and tillage, rather than other fundamental drivers. For more details on the environmental drivers, refer to You et al. (2022).

##### 2.2.1.1 Wheat Varieties

The model categorizes national winter wheat into three main varieties based on the classification of relative maturity groups: soft white winter wheat, hard red winter wheat, and soft red winter wheat. The growth characteristics of wheat under diverse temperature and precipitation conditions were well captured by these variety groups. A wheat variety map was created based on data from the National Association of Wheat Growers. Unlike the widespread cultivation of winter wheat, owing to the limited distribution of spring wheat, DLEM v4.0 includes only a single spring wheat variety for parameter calibration and simulation.

##### 2.2.1.2 Sowing Dates

The sowing dates in the DLEM v4.0 were dynamically simulated rather than prescribed. These dates depend on the sowing trigger criteria modified from CLM4.5 within the earliest and latest planting windows (You et al. 2022). The earliest and latest state‐level crop‐planting dates were obtained from the United States Department of Agriculture National Agricultural Statistics Service (USDA‐NASS) survey report (USDA NASS 2010), which provides planting and harvesting windows for most historical years. If environmental stress makes sowing impractical within the planting window, the model will suspend planting for that year. Consequently, wheat exposure to dry‐heat conditions varies annually with changes in sowing dates. Furthermore, the planting window for spring wheat is typically concentrated in spring, leading to its critical growth and developmental stages being consistently exposed to high temperatures and water scarcity during summer (even with delayed or advanced sowing dates). Therefore, it is foreseeable that spring wheat is inevitably more exposed to dry heat stress than winter wheat.

##### 2.2.1.3 Planted Area and Crop Rotation

Wheat production was calculated based on wheat yield and a dataset of dynamically changing annual planting areas. This dataset, encompassing annual crop types and crop rotation patterns from 1960 to 2018, was developed by combining the 30‐m Cropland Data Layer product (CDL) from the United States Department of Agriculture (USDA) and the National Agricultural Statistics Service (NASS) survey of county‐level crop planting areas using the spatialization method of Yu et al. (2018). We extracted the annual dynamic distribution and planting area of wheat from the crop‐rotation maps and allocated them to a 5‐arcmin spatial grid. Additionally, the dataset was utilized to simulate variations in GHG emissions resulting from land‐use changes.

##### 2.2.1.4 Manure and Nitrogen Fertilizer

According to Cao et al. (2018), annual crop‐specific nitrogen usage data from 1910 to 2018 were reconstructed using state‐level nitrogen use rates from the USDA‐NASS and national‐level commercial nitrogen consumption data from the USDA‐ERS (2019). Annual manure nitrogen application data from 1860 to 2018 were obtained from Bian et al. (2021).

##### 2.2.1.5 Irrigation

Annual crop‐specific irrigation data from 1950 to 2018 were downscaled based on county‐level irrigation reanalysis data (McManamay et al. 2021) and county‐level irrigated cropland areas from the USDA‐NASS using the MODIS Irrigated Agriculture Dataset as a baseline.

##### 2.2.1.6 Tillage

Annual tillage intensity data from 1960 to 2018 were reconstructed using county‐level tillage practice survey data from the National Crop Residue Management (CRM) Survey. Tillage maps for missing years were assumed to be the same as those from the nearest available years. We reorganized the original five tillage practices in the CRM dataset into four types by combining ridge‐ and mulch‐till practices with conservation tillage in the DLEM v4.0. The historical spatial distribution of tillage practices was estimated by integrating the county‐level CRM dataset with crop rotation maps derived from the CDL and crop planting area data from the USDA‐NASS.

##### 2.2.1.7 Other Meteorological Data

Two additional independent gridded meteorological datasets, Livneh and ERA5, were used for the uncertainty analysis of dry‐heat sensitivity. The Livneh hydrometeorological dataset consists of gridded daily precipitation, maximum and minimum air temperature, and wind speed for the continental U.S. at a 1/16° resolution (Livneh et al. 2015). The updated dataset extends the time range to 2018, matching the period of this study. The ERA5 reanalysis dataset, provided by the European Centre for Medium‐Range Weather Forecasts, offers global meteorological data at a 0.25° resolution for the period 1960–2018. Both datasets were re‐interpolated to a 5 arcmin resolution using bilinear interpolation to match the original resolution and assess wheat GHGI sensitivity to dry‐heat climates.

#### 2.2.2 Field Observations and Statistics

**FIGURE 1:**
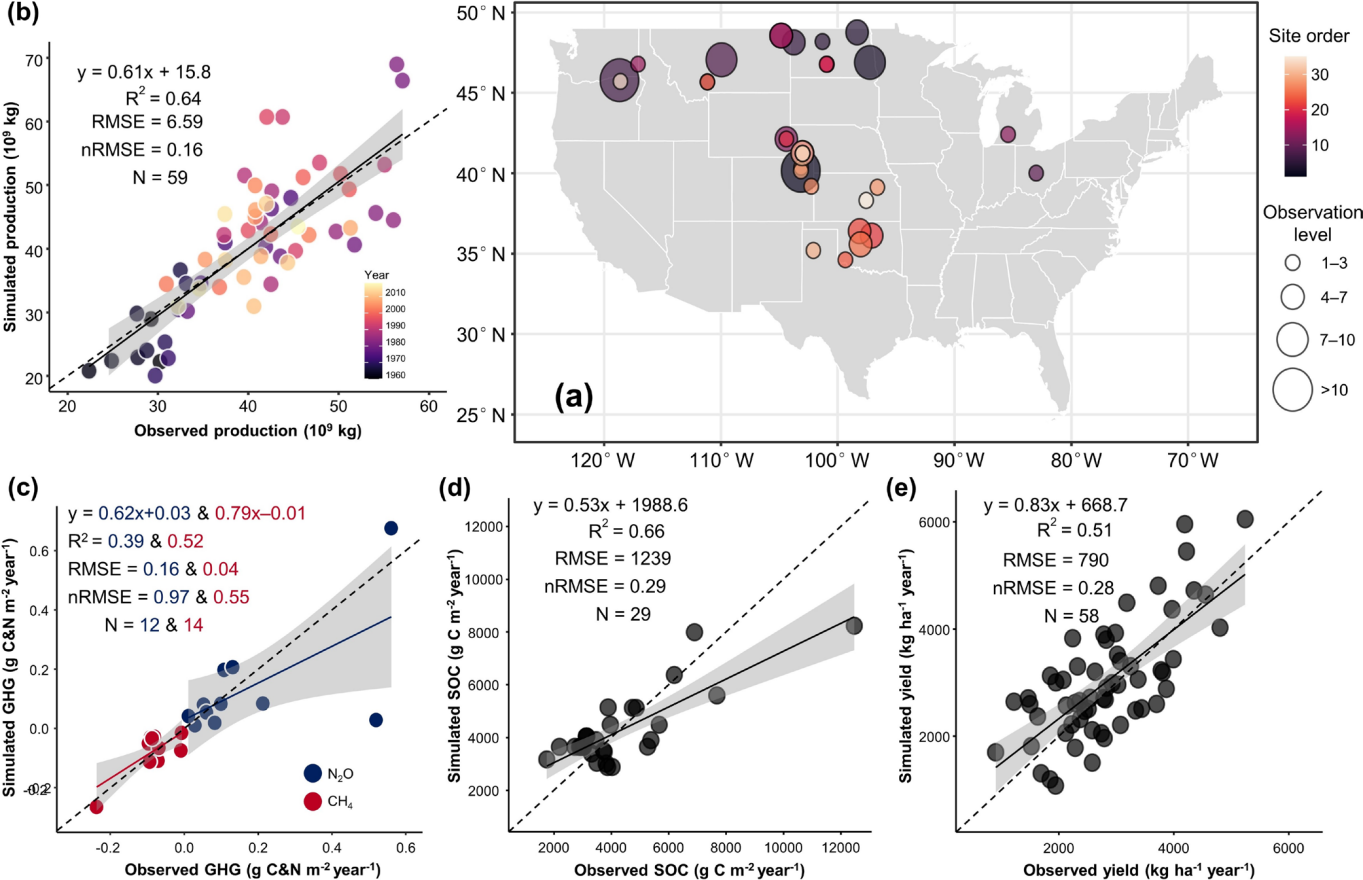
Spatial distribution of site observations and model validation. (a) Site‐level observations of annual N_2_O, CH_4_, SOC, and yield for wheat in the USA. The color gradient indicates the relative site order across all observations. The bubble size reflects the number of observations at each site. (b) Comparison between model‐simulated production and observed production. (c–e) Site‐level comparisons between model simulations and observations for greenhouse gases (c, N_2_O and CH_4_), SOC stock (d), and yield (e). The dashed and solid lines in each scatter plot represent the 1:1 line and the linear regression between observed and simulated values, respectively. Shaded bands indicate 95% confidence intervals for the mean predictions. Map lines delineate study areas and do not necessarily depict accepted national boundaries.

We conducted a comprehensive literature search of Google Scholar and Web of Science to identify peer‐reviewed publications reporting site‐scale wheat yields and soil GHG emissions in US croplands. The search keywords included “cropland or crop or wheat,” “the United States or America or U.S. or USA”, “soil organic carbon or SOC,” “methane or CH_4_,” “nitrous oxide or N_2_O,” and/or “greenhouse gases or GHG.” The identified papers were refined based on the following criteria to ensure data quality: (1) measurement in the field rather than in the laboratory; (2) supplemental information, such as cropping systems, experimental duration and conditions, and management practices (e.g., nitrogen fertilizer, tillage types, and irrigation); and (3) replicated field experiments. We identified annual data from 141 site‐years across 42 locations (Figure 1a and Figure S2), comprising 73 observations for yield, 32 observations for GHG emissions (N_2_O and CH_4_), and 36 observations for SOC stock (Table S3). These observations encompassed various management practices, including tillage (NT and CT), nitrogen fertilizer use, and irrigation. WebPlotDigitizer software was used to extract precise values when the data were presented graphically.

Wheat production statistics from 1960 to 2018 were collected from the USDA to evaluate the performance of DLEM v4.0, to simulate annual spatial variations in wheat production. Moreover, we employed wheat yield reports from 1960 to 2018 derived from the Southern Regional Performance Nursery, Northern Regional Performance Nursery, and Hard Red Spring Wheat Uniform Regional Nursery to examine dry‐heat‐driven sensitivity; this included 29 sites for winter wheat and 23 sites for spring wheat (Zhang et al. 2022). These sites cover the US Great Plains, subjected to less than 5% irrigation (Zhu and Burney 2021; Zhang et al. 2022). Common wheat varieties were used in this study (Kharkof for winter wheat and Matquis for spring wheat).

### 2.3 Model Calibration and Validation

The DLEM has been widely validated and applied to estimate N_2_O and CH_4_ emissions, as well as SOC stocks across multiple sites and large‐scale regions (Tian, Chen, et al. 2010; Tian, Xu, et al. 2010; Ren et al. 2012; Yu et al. 2018; Lu et al. 2022). It has also contributed to the Global Carbon Project for regional carbon and nitrogen cycle assessments (Friedlingstein et al. 2023; Tian et al. 2024). In this study, we rigorously calibrated and validated DLEM v4.0 using the environmental driver datasets outlined in Section 2.2.1 to better simulate SOC stocks and N_2_O and CH_4_ emissions in US wheat, leveraging field observations compiled from the metadata described in Section 2.2.2. In addition, we output simulations corresponding to the soil depths for validation because of the measurements of soil carbon stocks collected across varying soil depths (e.g., 0–15 cm or 0–30 cm). The coefficient of determination (*R* ^2^), root mean square error (RMSE), and normalized root mean square error (nRMSE) were employed to quantify the model performance.

Observations from 141 site years representing 42 U.S. sites covering major wheat cropping systems were used to calibrate, validate, and corroborate model simulations (Figure 1 and Figure S2). Specifically, we initially ran the model using default parameters and subsequently manually adjusted the parameter values within the reported range to achieve a close match between the observed and simulated values. During calibration, data from 15, 6, and 7 site years (approximately 20% of the original dataset) were used for yield, GHGs (N_2_O and CH_4_), and SOC stocks, respectively (Figure S3). Calibration for yield and GHGs was conducted separately for different tillage practices (NT and CT). Therefore, a parameter set from the calibration dataset that minimized the discrepancy between simulated and observed values was applied to regional validations.

After calibration, the remaining 80% of the field data were used to validate the model's regional simulations (Figure 1). We specifically focused on evaluating the model's performance in simulating the tillage effects on yield and GHGs. Therefore, additional validation was conducted under different tillage practices to compare their effects and simulation accuracy (Figure S4).

Finally, we performed a time‐series validation for production and GHGs (N_2_O and CH_4_) to assess the model's performance in simulating seasonal and interannual fluctuations. Interannual crop production statistics were obtained from the USDA‐NASS (Figure S5). Seasonal GHG observations were derived from long‐term site measurements at the Kellogg Biological Station Long‐term Ecological Research site (Figure S6). Wheat was planted at the ecological research station in 1995, 1998, 2001, 2004, 2007, 2010, and 2013, with continuous in situ measurements using static chambers and gas chromatography.

### 2.4 Experimental Design

The implementation of DLEM v4.0 consists of three main steps: an equilibrium run, a spin‐up run, and a transient run. The equilibrium run was driven by decadal average climate data during the 1860s period and other factors such as vegetation type, atmospheric CO_2_ concentration, and nitrogen deposition. The model assumes equilibrium is reached when changes in carbon, nitrogen, and water pools are less than 0.5 g C m^−2^ year^−1^, 0.5 g N m^−2^ year^−1^, and 0.5 mm m^−2^ year^−1^ over two consecutive 20‐year periods, respectively. The spin‐up run was conducted using detrended climate data from the 1860s to stabilize the fluctuations arising from the transition between the equilibrium and transient runs. Finally, the transient run was driven by historical data from 1860 to 2018, with simulations considered representative of real‐world changes. In this study, we focused on simulation results from 1960 to 2018, and model runs from 1860 to 1959 were considered as representing the slow accumulation of soil biogeochemical cycling.

We conducted 11 simulation experiments to determine the yield and GHG emission variations of US wheat (spring and winter wheat) induced by climate, CO_2_, land use and cover change (including crop rotations), nitrogen deposition, tillage, irrigation, and nitrogen fertilizer and manure application from 1860 to 2018 (Table S4). Specifically, the first simulation experiment (S1) keeps all drivers constant from the earliest available years. The second simulation experiment (S2) represents the optimal estimates of yield and GHG emissions in the US States driven by historical force inputs. The net yield and GHG variations driven by all factors were estimated as the difference between S1 and S2: Other experiments (S3–S9) were set with a driver that did not vary with time for each simulation (Table S4), and the contribution of the individual driver was acquired based on the results of “optimal estimates” (S2) minus “all‐drivers‐without‐one” (S3–S9). We also used the difference between S10 and S11 to quantify the effects of land use and land cover change (LULC, specifically referring to crop rotation), as changes in land cover may also influence inputs to agricultural management practices (such as nitrogen fertilizer and manure). Hence, for the two experiments, we maintained all management practices in 1860 to differentiate the contribution of LULC without management. Furthermore, two tillage scenarios were designed to simulate the potential effects of tillage practices on wheat yield and GHG emissions, where tillage practices were fixed as NT and CT (S12 and S13).

The sum of net GHGs from wheat soils was calculated based on the global warming potential (GWP) of CO_2_, CH_4_, and N_2_O on a 100‐year time scale from gram carbon and gram nitrogen to gram CO_2_ equivalents (IPCC 2021). Specifically, the GWP is the sum of the CO_2_ equivalents from the SOC sequestration of CO_2_, N_2_O, and CH_4_ emissions in wheat croplands:

(1) $$E_{{\mathrm{CO}}_{2}}=\left(F_{{\mathrm{CO}}_{2}}/12\right)\times 44$$

(2) $$E_{N_{2}O}=\left(F_{N_{2}O}/28\times 44\right)\times 273$$

(3) $$E_{{\mathrm{CH}}_{4}}=\left(F_{{\mathrm{CH}}_{4}}/12\times 16\right)\times 27$$

(4) $$E_{\mathrm{GHG}}=E_{{\mathrm{CO}}_{2}}+E_{N_{2}O}+E_{{\mathrm{CH}}_{4}}$$

(5) $$\text{GHGI}=E_{\mathrm{GHG}}/Y$$

where $F_{{\mathrm{CO}}_{2}}$, $F_{N_{2}O}$, and $F_{{\mathrm{CH}}_{4}}$ represent CO_2_ (Tg C year^−1^), N_2_O (Tg N year^−1^), and CH_4_ (Tg C year^−1^) fluxes, respectively. $E_{{\mathrm{CO}}_{2}}$, $E_{N_{2}O}$, and $E_{{\mathrm{CH}}_{4}}$ are the CO_2_, N_2_O, and CH_4_ emissions in Tg CO_2_ equivalents. Y, $E_{\mathrm{GHG}}$, and GHGI denote wheat production (Tg year^−1^ for total production; kg ha^−1^ for yield), net GHG emissions (Tg CO_2_‐eq year^−1^ for total emissions; kg CO_2_‐eq ha^−1^ for emissions per unit area), and GHG emission intensity (kg CO_2_‐eq kg^−1^), respectively.

### 2.5 Extreme Dry‐Heat Conditions

This study aimed to investigate how the carbon‐nitrogen cycle in wheat production responds to changes in extreme dry‐heat conditions (EDHs) under ongoing climate warming. To this end, we used the daily maximum temperature to characterize variations in heat conditions (HC), following the guidelines set by CCl/CLIVAR/JCOMM Expert Team on Climate Change Detection and Indices. Specifically, HC is defined as the number of days when the daily maximum air temperature exceeds 30°C for the period from March 1 through September 30 in each year (equation 6). This temperature threshold significantly surpasses the upper limit of the optimal temperature for photosynthesis in the absence of irrigation.

(6) $$\mathrm{HC}=\sum_{i=1}^{N}\left\{\begin{array}{c} 1\;T\max_{i}>30{}^{\circ}C\\ \begin{array}{cc} 0 & \text{otherwise} \end{array} \end{array}\right\}$$

where $T\max_{i}$ denotes the daily maximum air temperature on *i*‐th day between March and September, and *N* represents the total number of days in this period.

Additionally, we characterized dry conditions (DC, i.e., environmental water scarcity) using the aridity index (AI), calculated at daily intervals as the ratio of daily precipitation (*P*, mm year^−1^) to potential evapotranspiration (ET_P_, mm year^−1^). Specifically, *DC* is defined as the number of days when the AI falls below 0.2 for the period from March 1 through September 30 in each year (equation 10). An AI value below this threshold signifies the onset of aridity at a given time and location and is widely applied to identify the global occurrence of aridity conditions (Middleton and Thomas 1992; Berg and McColl 2021; Shi et al. 2021). ET_P_ was calculated using the well‐known Hargreaves model, which is constrained by solar radiation and temperature (Hargreaves and Samani 1982; Shi et al. 2020). Previous studies proved that air temperature and solar radiation can capture more than 80% of ET_P_ variations (Almorox et al. 2015; Shi et al. 2020). The formulae are parameterized as follows:

(7) $$\mathrm{AI}=P/{\mathrm{ET}}_{P}$$

(8) $${\mathrm{ET}}_{p}=0.0023\times 0.408R_{a}\times {\left(T_{\max}-T_{\min}\right)}^{0.5}\times \left(T+17.8\right)$$

(9) $$R_{a}=\frac{S_{0}}{\pi }{\left(\frac{r_{0}}{r}\right)}^{2}\left(H\sin\phi \sin\delta +\sin H\cos\phi \cos\delta \right)$$

(10) $$\mathrm{DC}=\sum_{i=1}^{N}\left\{\begin{array}{l} \begin{array}{cc} 1 & {\mathrm{AI}}_{i}<0.2 \end{array}\\ \begin{array}{cc} 0 & \text{otherwise} \end{array} \end{array}\right\}$$

where *R*_a_, *T*_max_, and *T*_min_ are the extraterrestrial solar radiation (MJ m^−2^ d^−1^), maximum air temperature (°C), and minimum air temperature (°C), respectively; T (°C) is the mean daily air temperature at a height of 2 m; *S*_0_ is solar constant (118.02 MJ m^−2^ d^−1^); *r* is the Earth‐Sun distance; *r*_0_ is the mean Earth‐Sun distance; H is sun hour angle at sunset; ϕ is latitude (°); and δ is solar declination (°). AI_*i*_ denotes the aridity index on *i*‐th day between March and September, and *N* represents the total number of days in this period. For the compound effects of dry‐heat conditions (CDHC), we defined them as the number of days with simultaneous dry and heat conditions for the period from March 1 through to September 30 in each year (equation 11). This approach has been extensively utilized in previous studies of compound events, and thus, we did not consider cascading effects between dryness and heat in this study. CDHC is parameterized as follows:
(11)

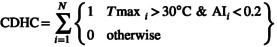




### 2.6 Sensitivity to EDHs and Environment‐Specific Tillage Scheme

The crop GHGI sensitivity to EDHs is typically described as a coupling relationship in terms of direction and magnitude between the GHGI and EDHs within specific periods. Here, we adopted the Pearson correlation coefficient (*R*) to characterize the sensitivity variation between EDHs (HC, DC, and CDHC) and wheat GHGI (yield and GHGs). The sign of *R* indicates the direction of sensitivity, whereas its magnitude signifies the sensitivity strength. To detect spatiotemporal trends in the GHGI sensitivity to EDHs, we employed linear regressions based on Pearson correlations from a 20‐year moving window method over a six‐decade period (1960–1979 to 2009–2018). Specifically, all the applied variables were linearly detrended during the six‐decade period. Then, all correlation results in a given time window were indexed to the previous year; that is, *R*_EDH‐GHGI_ in 1979 represents the correlation between EDHs and wheat GHGI from 1960 to 1979. Finally, we calculated the slope and significance (*p*‐value) in each grid using the linear regression method to analyze significant trends (*p* < 0.05). In addition, for the sensitivity variations of whole regions, we used the Mann‐Kendall's test to detect overall trends and significance, which does not require data with normal distribution. Based on the same approach, we calculated the spatiotemporal patterns of the GHGI sensitivity under different tillage scenarios (CT and NT, Table S4).

From the perspective of decreasing sensitivity, the benefits of environment‐specific tillage in resisting dry‐heat events were further explored. Initially, the effects of tillage practices were defined as the differences between the annual sensitivity variations under the actual scenario with all drivers and those under the tillage scenarios. For specific grids and sliding windows, the changes in sensitivity between tillage scenarios (S12 and S13 in Table S4) and the actual scenario (S2 in Table S4) were determined as effects induced by alterations in tillage practices (NE, negative effect; PE, positive effect). The specific rules are as follows.

(12) $$\left\{\begin{array}{c} \begin{array}{cc} \text{i}f & \begin{array}{cc} {\mathrm{DY}}_{t-a}>0, & \begin{array}{cc} \mathrm{PE} & \end{array} \end{array} \end{array}\\ \begin{array}{cc} \text{if} & \begin{array}{cc} {\mathrm{DY}}_{t-a}<0, & \begin{array}{cc} \mathrm{NE} & \end{array} \end{array} \end{array}\\ \begin{array}{cc} \text{if} & \begin{array}{cc} {\text{DGHG}}_{t-a}>0, & \begin{array}{cc} \mathrm{NE} & \end{array} \end{array} \end{array}\\ \begin{array}{cc} \text{if} & \begin{array}{cc} {\text{DGHG}}_{t-a}<0, & \begin{array}{cc} \mathrm{PE} & \end{array} \end{array} \end{array}\\ \begin{array}{cc} \text{if} & \begin{array}{cc} {\text{DGHGI}}_{t-a}>0, & \begin{array}{cc} \mathrm{NE} & \end{array} \end{array} \end{array}\\ \begin{array}{cc} \text{if} & \begin{array}{cc} {\text{DGHGI}}_{t-a}<0, & \begin{array}{cc} \mathrm{PE} & \end{array} \end{array} \end{array} \end{array}\right.$$

where ${\mathrm{DY}}_{t-a}$, ${\text{DGHG}}_{t-a}$, and ${\text{DGHGI}}_{t-a}$ represent the sensitivity differences in yield, GHG emissions, and GHGI between the tillage and actual scenarios in each 20‐year moving window, respectively. Moreover, the frequency of tillage effects (PE or NE) between 1960–1979 and 2009–2018 was quantified to assess the overall tillage impact. For example, if the occurrence frequency of positive effects falls below 50%, the region is predominantly governed by the negative consequences of tillage.

A significant effect zone was defined when the occurrence frequency of positive tillage effects exceeded 75%, indicating that particular tillage practices have a probability greater than 75% for mitigating dry‐heat sensitivity in wheat. If tillage effects remain positive across all moving window periods, tillage practices in these specific areas can fully mitigate the negative impacts of extreme dry heat events.

Therefore, the implementation of tillage practices in specific grids was determined based on significant effects (occurrence frequency of positive effects > 75%) of tillage practices (NT or CT). Optimal tillage maps were generated by superimposing the spatial distribution of the significant effects arising from the different tillage practices (NT and CT). Finally, we utilized these tillage maps to simulate changes in the sensitivity of yield, GHG emissions, and GHGI to EDHs, to characterize the potential of climate‐adapted tillage practices.

### 2.7 Uncertainty Analysis

The dry‐heat conditions derived from a single meteorological dataset may influence the estimation of sensitivity trends and magnitudes, potentially limiting the generalizability and robustness of the findings. Moreover, the sensitivity of GHGs to EDH climate using Pearson's correlation may be inadequate for capturing potential non‐linear relationships between these variables. To address these limitations, we introduced two additional independent meteorological datasets (Livneh and ERA5) and employed non‐linear methods (Kendall and Spearman correlations) for comparative analysis. We integrated all datasets and methods to generate nine sets of sensitivity results, providing a comprehensive basis for uncertainty analysis. The uncertainty in the simulated sensitivity was expressed as the standard error (SE):

(13) $$\mathrm{SE}=\frac{s}{\sqrt{n}}$$

where *s* and *n* represent the standard deviation and sample size, respectively.

We previously conducted parameter sensitivity tests for DLEM v4.0 using a Monte Carlo sampling approach to evaluate its performance in simulating N_2_O, CH_4_, and SOC. Specifically, based on the probability distribution functions, parameters were randomly varied within 20% of their calibration values to generate 100 sets of parameter scenarios. The parameter sets were used as inputs for the DLEM to simulate wheat carbon and nitrogen balances and to compute standard deviations. The parameter sensitivity results previously presented by You et al. (2024) are not shown again in this study. Additional parameter sensitivity analyses and more detailed validation of the LAI and aboveground biomass are outlined by You et al. (2022).

## 3 Results

### 3.1 Model Performance Evaluation

We rigorously calibrated and validated the DLEM to simulate net GHGs (N_2_O, CH_4_, and CO_2_) and wheat yield at both site and regional scales, with a particular focus on model performance across different tillage practices (NT and CT). For site‐level calibration, 20% of the 141 collected observations (73 for yield, 32 for GHGs, and 36 for SOC stock) were randomly selected. The calibrated model demonstrated robust performance, achieving *R* ^2^ values of 0.56 for yield, 0.67 for SOC stock, and 0.85 for GHG emissions (Figure S3). Validation with the remaining data indicated that the model explained 66%, 64%, and 52% of the variance (*R* ^2^) in net GHGs, SOC, and wheat yield at the regional level, respectively (Figure 1c–e). The model effectively captured wheat productivity and emissions across varying yield conditions, exhibiting low simulation error relative to observational data (Figure 1). Although there were occasional discrepancies in the N_2_O simulations, the majority of simulated values closely approximated the observed data.

Next, model simulations under CT and NT management practices were compared with field observations (Figure S4). The results showed that the calibrated model reliably reproduced wheat yield, SOC, and GHG emissions (primarily N_2_O) under NT and CT practices, showing close agreement with observations and strong correlations (Figure S4). This further confirms the model's effectiveness in simulating wheat productivity and associated GHG emissions under contrasting tillage practices.

Finally, we compared interannual and seasonal variations in GHG emissions and yield between measured and simulated values using annual national production statistics and observations from a long‐term ecological research site (Figures S5 and S6). The calibrated model accurately simulated the magnitude of national production (*R* ^2^ = 0.64, Figure 1b) and captured interannual variability in survey data (Figure S5). Moreover, the model performed well in estimating seasonal variations in soil N_2_O and CH_4_ fluxes at a wheat experimental site (Figure S6). While a few observations deviated from simulated values, these discrepancies were likely associated with greater uncertainty, potentially due to systematic errors in the original experimental design involving four replicates.

### 3.2 US Wheat GHGI Magnitudes and Trends From 1960 to 2018

**FIGURE 2:**
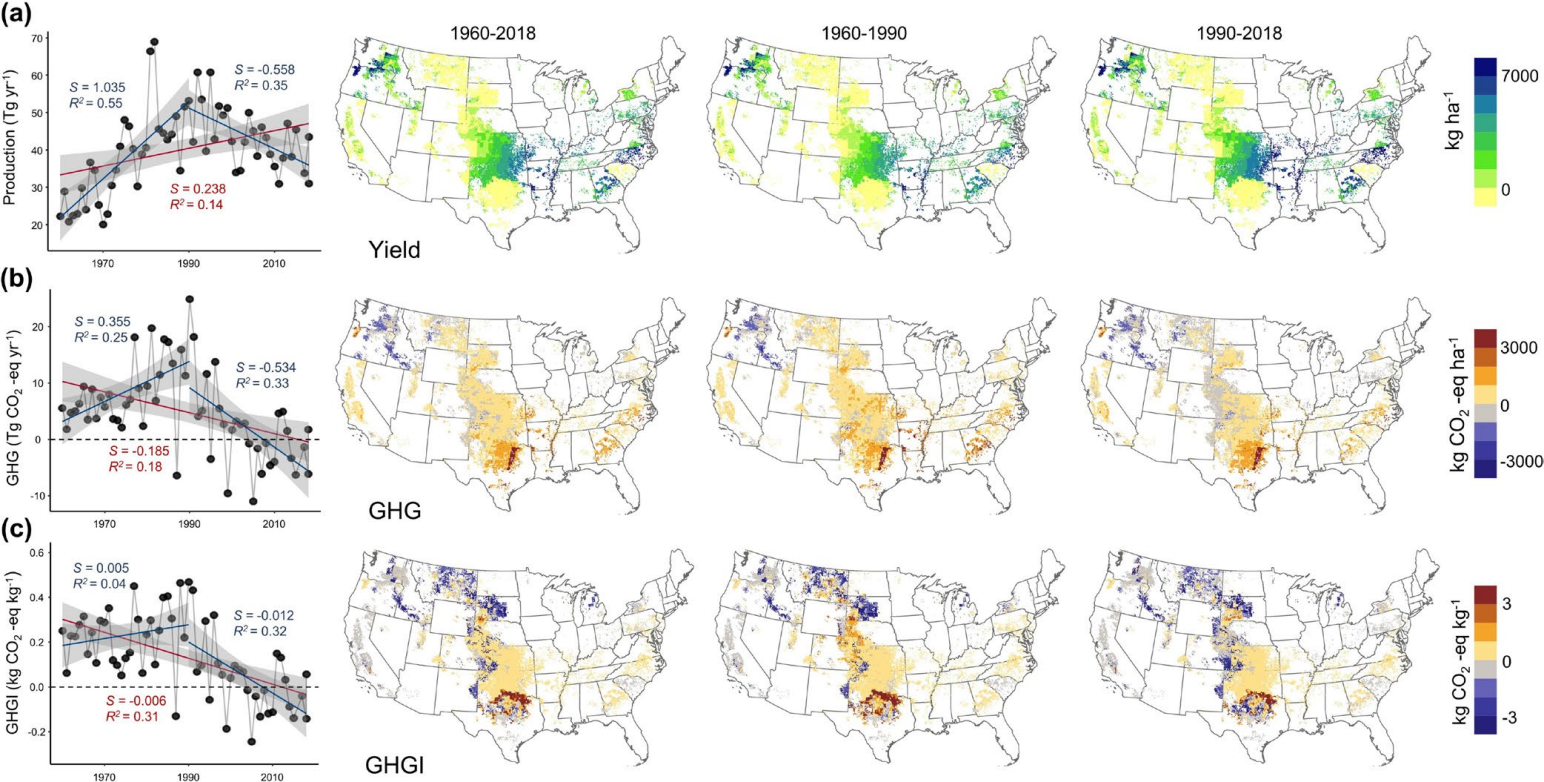
Spatial–temporal variations in production, net greenhouse gas (GHG) emission, and GHG emission intensity for winter wheat in the United States (1960–2018). Results were simulated using the Dynamic Land Ecosystem Model (v4.0). Line graphs show trend lines for two sub‐periods (1960–1990 and 1990–2018) and the entire period (1960–2018), which are represented by two solid blue lines and one solid red line, respectively. Shaded bands indicate 95% confidence intervals for the mean predictions. Negative values for GHG emission and GHG emission intensity in each graph indicate uptake. Map lines delineate study areas and do not necessarily depict accepted national boundaries.

Our simulation results showed that US wheat production has contributed an estimated 55.6 Tg of production per year (72.3% from winter wheat and 27.7% from spring wheat, Figure 2a) and 5.6 Tg of GHGs per year (88.0% from winter wheat and 12.0% from spring wheat, Figure 2b) over the past six decades. The production, net GHG, and GHGI of winter wheat increased substantially from 1960 to 1990 but experienced a significant decline from 1990 to 2018 (Figure 2a–c, and Table S5). In comparison, spring wheat showed a marked increase in the GHGI owing to significant increases in GHGs and production during this period (Figure S3 and Table S5). The spatial patterns of GHGs and yield for winter wheat showed considerable heterogeneity, with higher GHGI and net GHGs distributed in the central southern US, which coincided with regions of high yield from 1960 to 2018. Conversely, winter wheat in the northern US acts as a GHG sink, resulting in a decrease in the GHGI (Figure 2). In addition, there was a negative trend in spring wheat production after 1990, in sharp contrast to the significant increase observed over the preceding three decades (Figure S7a). Overall, the GHG emissions and yields of spring wheat were much lower than those of winter wheat, and no prominent cold or hot spots were observed in the spatial patterns (Figure S7).

### 3.3 Attribution Analysis of Variations in GHG Emissions and Yield From 1960 to 2018

**FIGURE 3:**
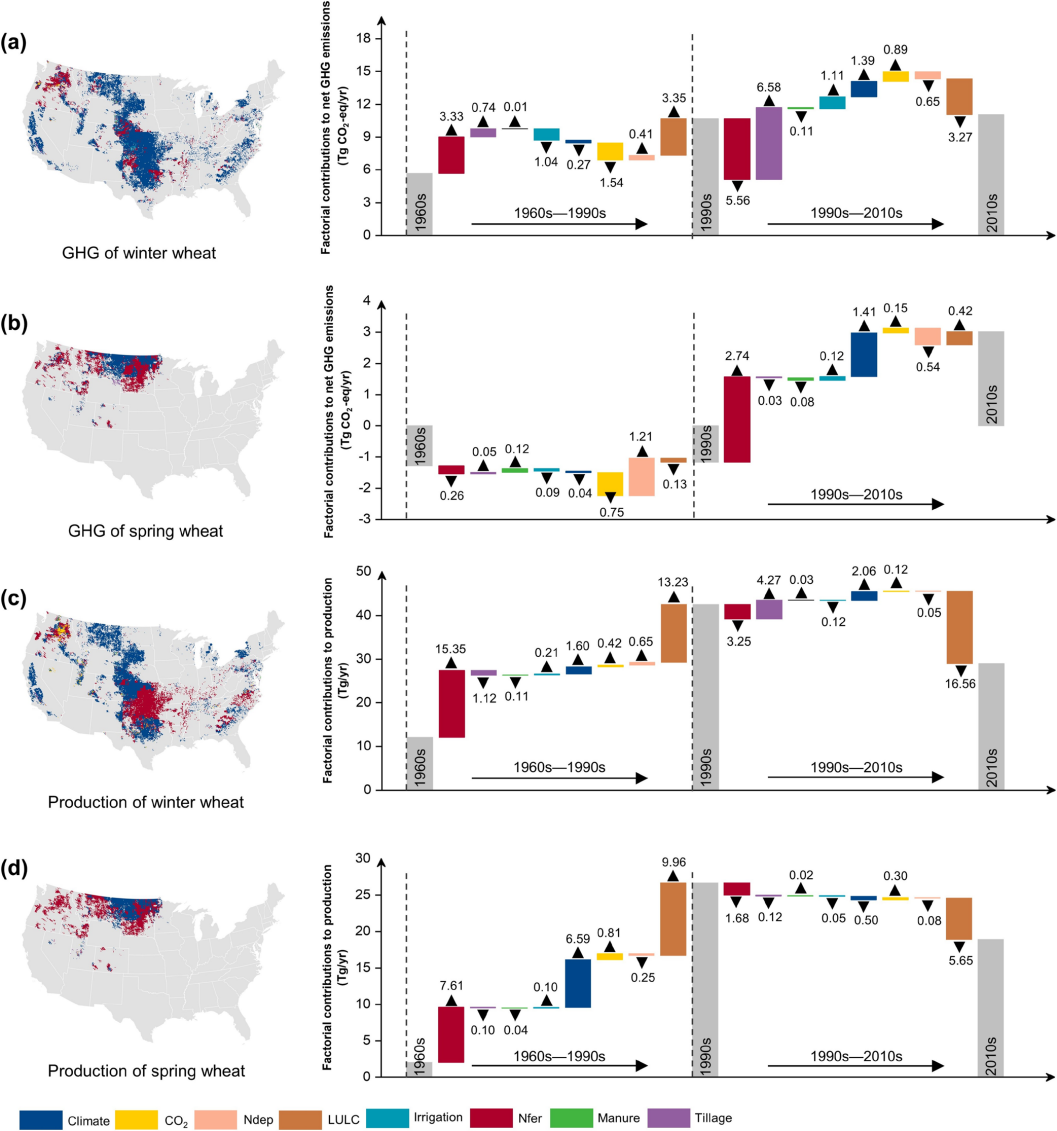
Spatial–temporal contribution of multi‐environmental factors to net greenhouse gas (GHG) emissions and yield for wheat in the United States. Spatial–temporal patterns of dominant factors for winter wheat (panels a and c) and spring wheat (panels b and d) from 1960 to 2018 were derived from scenario‐based simulations. The contribution of each environmental factor was quantified as the difference between the all‐drivers scenario and the corresponding single‐factor scenario, as detailed in the experimental design (Section 2.4 and Table S4). The maps illustrate the spatial contributions of environmental factors to wheat emissions per unit area and yield, while the bar plots summarize their contributions to total wheat emissions and production. The direction of the black triangle in the bar plots indicates the variation in the contribution of environmental factors to net GHG emissions and wheat production during specific periods. Ndep: Nitrogen deposition; LULC: Land use and land cover change; and Nfer: Nitrogen fertilizer application. Map lines delineate study areas and do not necessarily depict accepted national boundaries.

We conducted an attribution analysis based on a set of simulation experiments to investigate the impacts of climate, CO_2_, LULC, nitrogen deposition, and agricultural management practices on US wheat yield and GHG emissions from 1960 to 2018, with the experimental design described in Section 2.4 and Table S4. The results showed that climate change and nitrogen fertilization (Nfer) were the dominant drivers of changes in GHG emissions and yield across the region (excluding the impact of LULC on yield). Anthropogenic tillage practices and atmospheric CO_2_ fertilization played more significant roles in certain parts of the wheat‐growing regions (Figure 3). We also conducted a further analysis of two distinct periods, 1960–1990 and 1990–2018, which revealed that Nfer, LULC, and tillage management were the key factors influencing temporal variations in GHG emissions and winter wheat production over time.

Specifically, the contributions of Nfer and LULC to the total GHG emissions of winter wheat increased before the 1990s but declined over the subsequent three decades (Figure 3). This trend closely aligns with the changes in harvested area and total nitrogen fertilizer inputs, which first increased and then decreased (Figures S8 and S9). Therefore, the substantial decline in harvested area and total nitrogen fertilizer inputs since the 1990s has significantly driven the decrease in the contributions of LULC and nitrogen fertilizer use to production and GHG emissions (Figures S8 and S9). With little effect on production, changes in tillage practices led to a substantial increase in GHG emissions from 1990 to 2018 by offsetting emission reduction efforts due to reduced nitrogen fertilizer use (Figure 3a,c). Unlike winter wheat, LULC did not contribute to increased GHG emissions from spring wheat, and Nfer and climate change were the primary factors driving the changes in net GHG emissions and production over time. However, LULC plays an essential role in spring wheat production. The increased production of spring wheat was dominated by climate, Nfer, and LULC during the 1960s and the 1990s (Figure 3b,d). After the 1990s, LULC hindered spring wheat production (Figure 3d), which decreased significantly (Figure S7).

We further quantified the influence of multiple environmental factors on individual GHG emissions (N_2_O, CO_2_, and CH_4_), along with the spatial patterns of GHG emissions induced by historical tillage practices (Figure S10). For winter wheat, nitrogen fertilizer application predominantly drove variations in N_2_O emissions (Figure S10b), whereas historical climate variability controlled the net CO_2_ and CH_4_ emissions across the US (Figure S10d,f). Similarly, net emissions of N_2_O and CO_2_ were primarily influenced by increased nitrogen fertilizer application in spring wheat. Over the past 60 years, tillage practices have primarily affected variations in CO_2_ emissions throughout US wheat‐growing regions (Figure S10g,h). Additionally, tillage activities contributed to the observed proportion of N_2_O net emissions in the winter wheat‐growing areas, particularly in the central and western coastal regions (Figure S10h).

### 3.4 Sensitivity of Wheat GHG Emissions and Yield to Extreme Dry‐Heat Events

Over the past six decades, US wheat‐growing regions have experienced varying levels of EDHs, with more than 75% of the growing regions at risk of aridity occurrence (Figure S11). The risk of high temperatures and compound events with aridity also increased from north to south along the latitudinal distribution (Figure S11). Although no significant temporal trend was observed in heatwave events and their compounds at high temperatures (Figure S11), the wheat‐growing regions experienced consistent drying. From 1960 to 2018, there were substantial increases in the aridity periods for winter wheat (2.3 days/10 years, *p* < 0.01) and spring wheat (1.6 days/10 years, *p* < 0.01) (Figure S11c). Consequently, the frequency of compound dry heat events may increase in the future, further exacerbating potential carbon losses.

**FIGURE 4:**
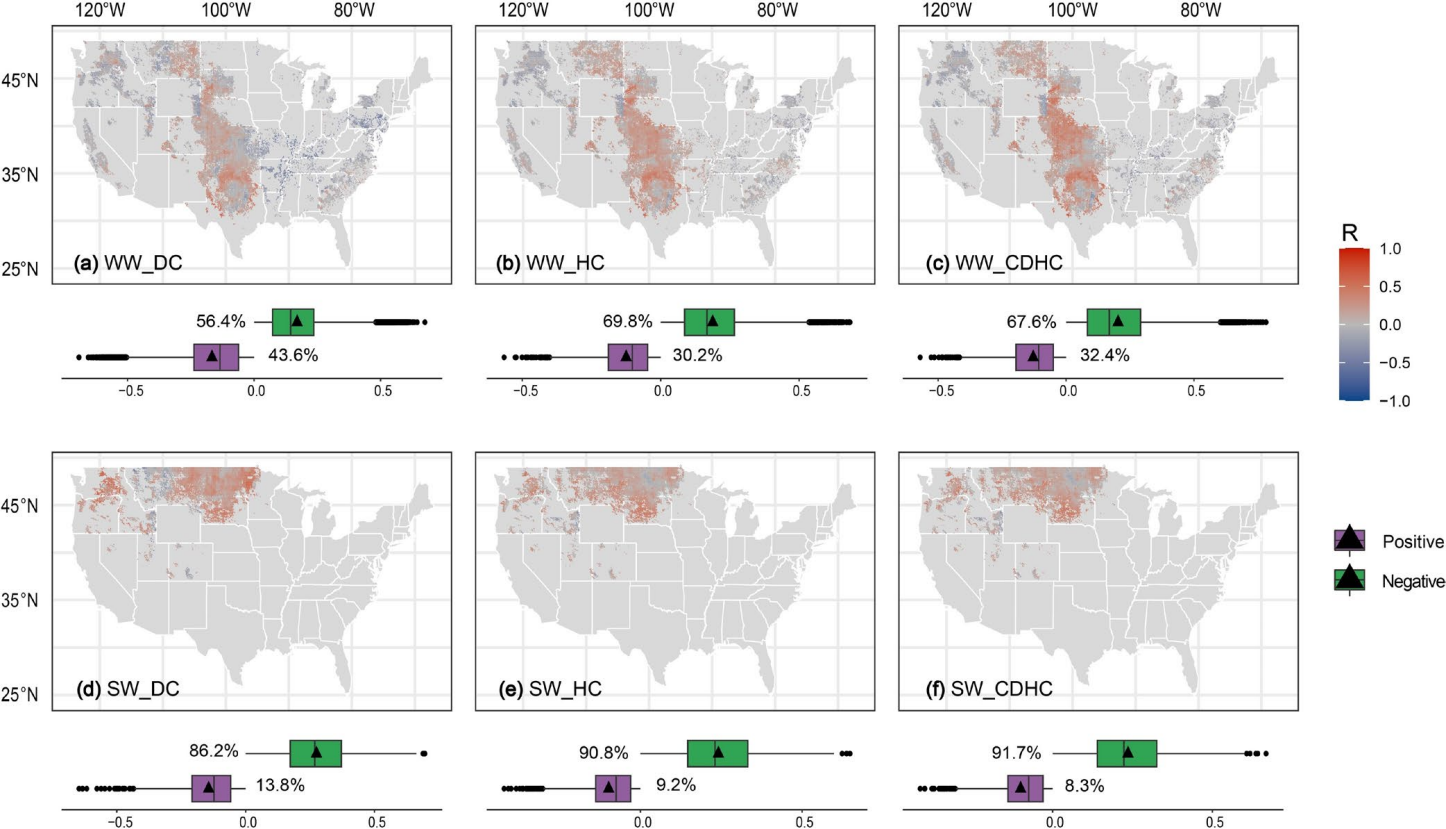
Sensitivity of wheat greenhouse gas (GHG) emission intensity to dry‐heat events from 1960 to 2018. Pearson correlation coefficients (*R*) are used to reveal the sensitivity of GHG emission intensity to dry‐heat events. The box plots summarize the distribution of pixel‐level sensitivity values (x‐axis) derived from gridded maps, separately aggregated for regions showing negative (green) and positive (purple) responses of GHGI to EDH. The percentages adjacent to each box indicate the proportion of the wheat‐growing region exhibiting each response type. In each boxplot, black triangles indicate mean values and black points denote outliers. WW (panel a–c) and SW (panel d–f) are winter wheat and spring wheat, respectively. DC, HC, and CDHC represent heat, dry, and compound dry‐heat conditions, respectively. Map lines delineate study areas and do not necessarily depict accepted national boundaries.

EDHs have exacerbated GHGI in a significant portion of winter (~70%) and spring wheat (~90%) regions across the US (Figure 4). This adverse effect caused by extreme climates is particularly evident in the central Great Plains region of the US (Figure 4). Dry‐heat events caused an increase in net GHG emissions from 1960 to 2018 in 67.7% ± 7.4% of winter wheat‐growing regions and 91.3% ± 1.9% of spring wheat‐growing regions (Figure S12). Across the wheat region, high‐temperature events had a more significant negative impact on GHG emissions than aridity (Figure 4 and Figure S12). The projected increase in aridity could further amplify adverse effects on GHGs (Figure 4 and Figure S11). EDHs triggered CO_2_ and CH_4_ release from winter wheat but suppressed N_2_O emissions (Figure S13). Given that the total CH_4_ emissions in the wheat‐planted areas were negligible, the CO_2_ emissions induced by EDHs contributed the most to GHG emissions. In contrast, more GHGs from spring wheat have been attributed not only to EDHs that cause large CO_2_ emissions but also to aridity events that release more N_2_O (Figure S14).

Increasing EDHs resulted in significant reductions in wheat yield, particularly in the central–southern US (Figure S12). The impact of dry‐heat conditions on yield was also evident in most regions, with 76.7% ± 0.7% of winter wheat‐production regions and nearly 86.9% ± 13.7% of spring wheat‐production regions experiencing reduced yields (Figure S12d–f). While modest aridity may be beneficial for spring wheat grown in the northern regions of the US, this advantage is likely to diminish as the area becomes more arid (Figure S12j–l).

### 3.5 Sensitivity Variations in Wheat GHG Emissions and Yield to Extreme Dry‐Heat Events

**FIGURE 5:**
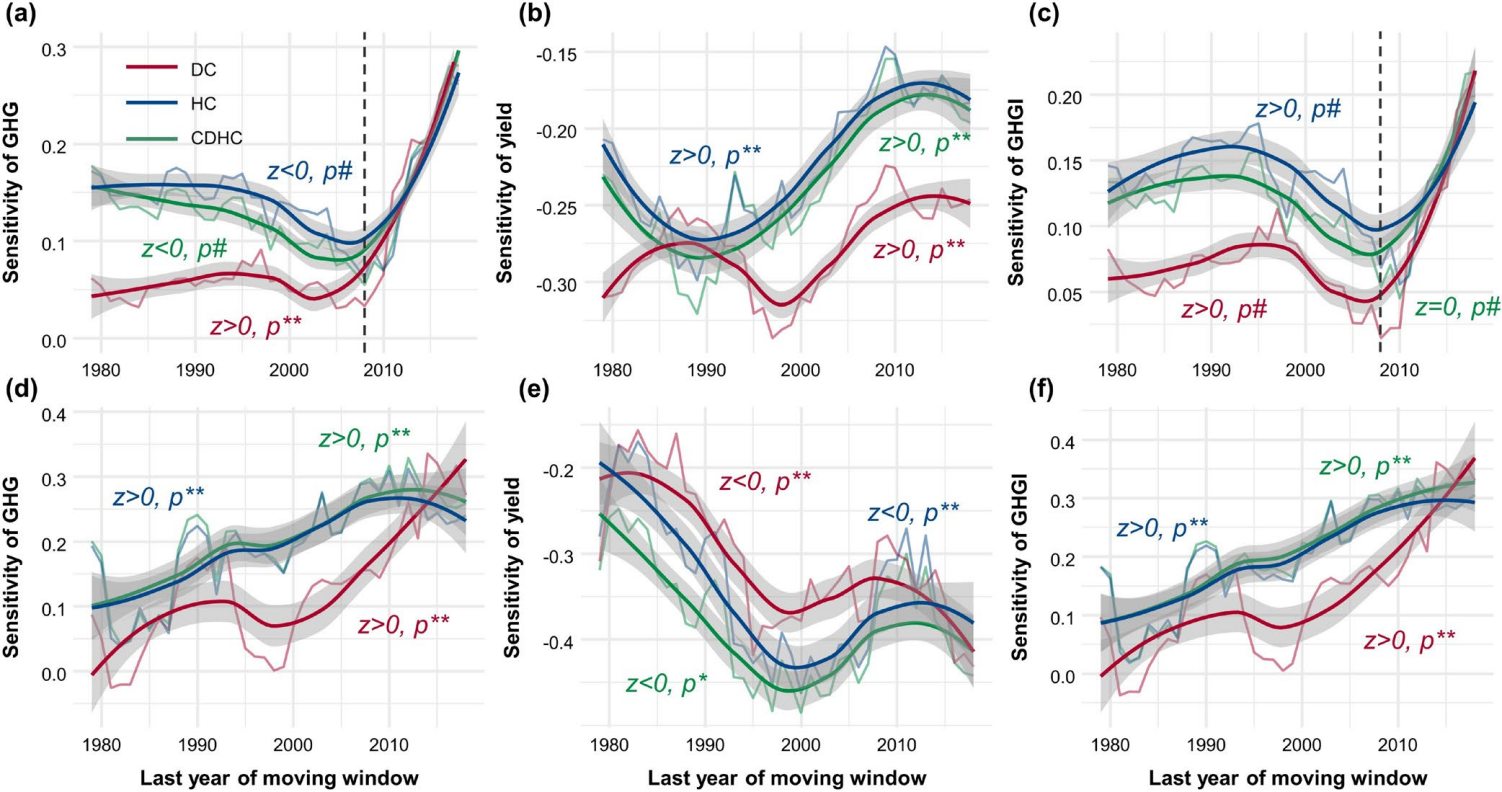
Temporal dynamics in the sensitivity of wheat GHG emissions (kg CO_2_‐eq/ha) and yield (kg/ha) to dry‐heat conditions across the United States. Net GHG emissions (panels a and d) of winter wheat (a–c) and spring wheat (d–f), as well as their yield (panels b and e) and GHG emission intensity (panels c and f) under dry‐heat conditions are shown. The annual sensitivity variations were calculated as the median value from spatial grids. In panel (a), the dashed line represents the year 2008, while the solid lines indicate the loess regression fitting. Shaded bands indicate 95% confidence intervals for the mean predictions. Symbols indicating the significance test of trends are as follows: ^#^ *p* > 0.05, *p < 0.05, and **p < 0.01. DC, HC, and CDHC represent heat, dry, and compound dry‐heat conditions, respectively.

Varying temporal dynamics in wheat production sensitivity to EDHs were observed in spring and winter wheat (Figure 5). Specifically, the sensitivity of wheat GHGs to EDHs has intensified over the years, resulting in a significant increase in the GHGI (Figure 5). The sensitivity of GHGs in spring wheat has significantly increased over the past six decades under EDHs, and the sensitivity of winter wheat after 2008s also showed a significant increase (*p* < 0.01; Figure 5a,d). Aridity‐related negative effects are approaching or even exceeding those at high temperatures for wheat GHGs and their intensities (Figure 5). Although the detrimental impact of dry heat on winter wheat yield steadily weakened statistically (*p* < 0.01), adverse effects for spring wheat significantly increased, especially for aridity under sustained drying (Figure 5b,e).

**FIGURE 6:**
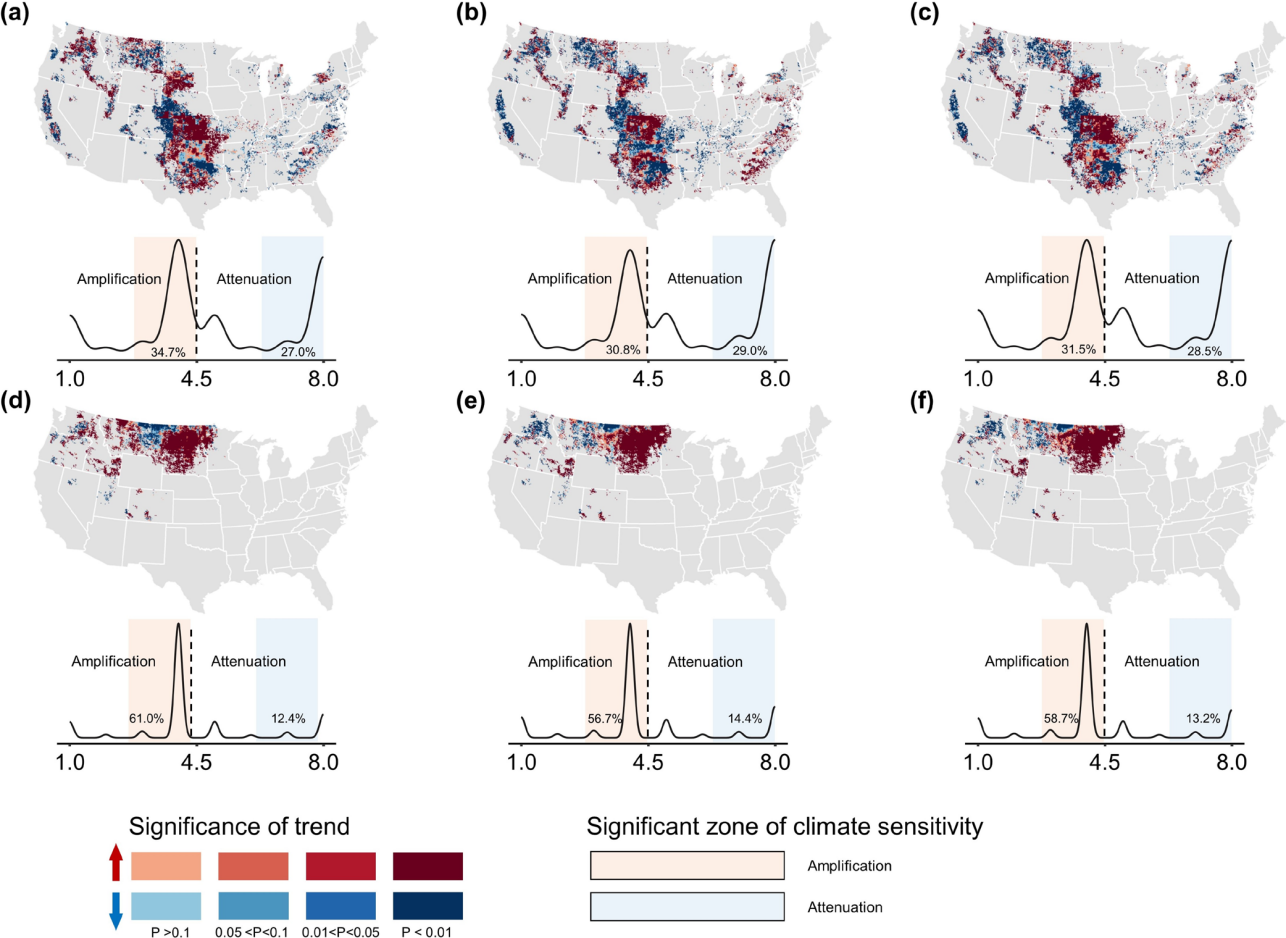
Spatial patterns of GHGI sensitivity variations to dry‐heat conditions for winter wheat (panels a–c) and spring wheat (panels d–f). The panels show sensitivity variations under dry (a, d), heat (b, e), and compound dry‐heat conditions (c, f). Linear trends and their significance are calculated at the pixel level. The probability density plots below each map summarize the distribution of classified pixels, with x‐axis values 1–4 (red) and 5–8 (blue) indicating increasing and decreasing sensitivity, respectively. Higher values reflect stronger statistical significance, and orange and blue bands highlight regions with significant changes (*p* < 0.05). These classifications correspond to the color bands in the maps. Map lines delineate study areas and do not necessarily depict accepted national boundaries.

The sensitivity dynamics of the wheat GHGI to EDHs also exhibited distinct regional characteristics (Figure 6). Across US wheat regions, the negative impacts of EDHs on wheat GHGs in 2018 (the last year in the 20‐year sliding window; also applies to the remaining text) were considerably higher than those in 1979 (Figures S15a, S16a, and S17a). This resulted in a more dramatic wheat GHGI controlled by EDHs than it was 60 years ago (Figure 6). Moreover, the adverse impacts of EDHs increased significantly over time in the south‐central US of the winter wheat planted regions and the majority of spring wheat planted regions (Figure 6, Figures S15b, S16b, and S17b). A few sporadic regions are favored by dry‐heat climates, such as the northern areas of winter wheat and the western areas of spring wheat. Significant increases (*p* < 0.05) in GHGI sensitivity to EDHs were observed in 32.3% ± 1.7% of winter wheat areas and 58.8% ± 1.8% of spring wheat areas in the US, indicating that climate costs in wheat under dry‐heat conditions were further amplified over time (Figure 6). Additionally, the probability distribution revealed that aridity had a more detrimental impact on the GHGI in recent years than heat waves and their combined effects (Figures S15b, S16b, and S17b). This trend was evident across a broader region of the US.

Significant regional trends underscored that the impact of EDHs on yield shifted from positive to negative in the eastern spring wheat region and caused yield fluctuations in other regions (Figures S15a, S16a, and S17a). In contrast, the sensitivity of winter wheat yields to EDHs showed large regional heterogeneity. This was also confirmed by the results derived from 29 winter wheat and 23 spring wheat nursery statistics spanning 1960–2018 (Figure S18). At most experimental stations, the wheat yields experienced negative dry‐heat climate shocks (e.g., 20 of 29 winter wheat observations and 15 of 23 spring wheat observations under a compound climate). Significant increases in negative impacts on yield under dry‐heat climate were observed at 29.9% and 27.5% of stations (Figure S18).

### 3.6 Potential of Environment‐Specific Tillage Practices in Mitigating Negative Impacts of EDHs on GHGI

We first assessed the impact of switching from historical tillage to specific tillage practices (CT and NT) on GHG emissions (Figures S19 and S20). Our findings demonstrate that the adoption of CT across the entire wheat‐growing region resulted in a substantial release of N_2_O and CO_2_ (Figures S19a,c and S20). Even with the transition to NT, the CO_2_ emissions from spring wheat could not be completely avoided (Figure S19d). However, NT presents a greater opportunity to reduce N_2_O emissions, particularly for spring wheat (Figure S19b,d). The negative effects of switching between individual tillage practices extended throughout the wheat‐growing region. Consequently, untimely tillage practices hinder food production and contribute to GHG emissions at spatial–temporal scales. To address these gaps, we developed an optimal tillage scheme based on spatiotemporal and environment‐specific conditions to mitigate the negative impacts of dry‐heat events on the GHGI.

**FIGURE 7:**
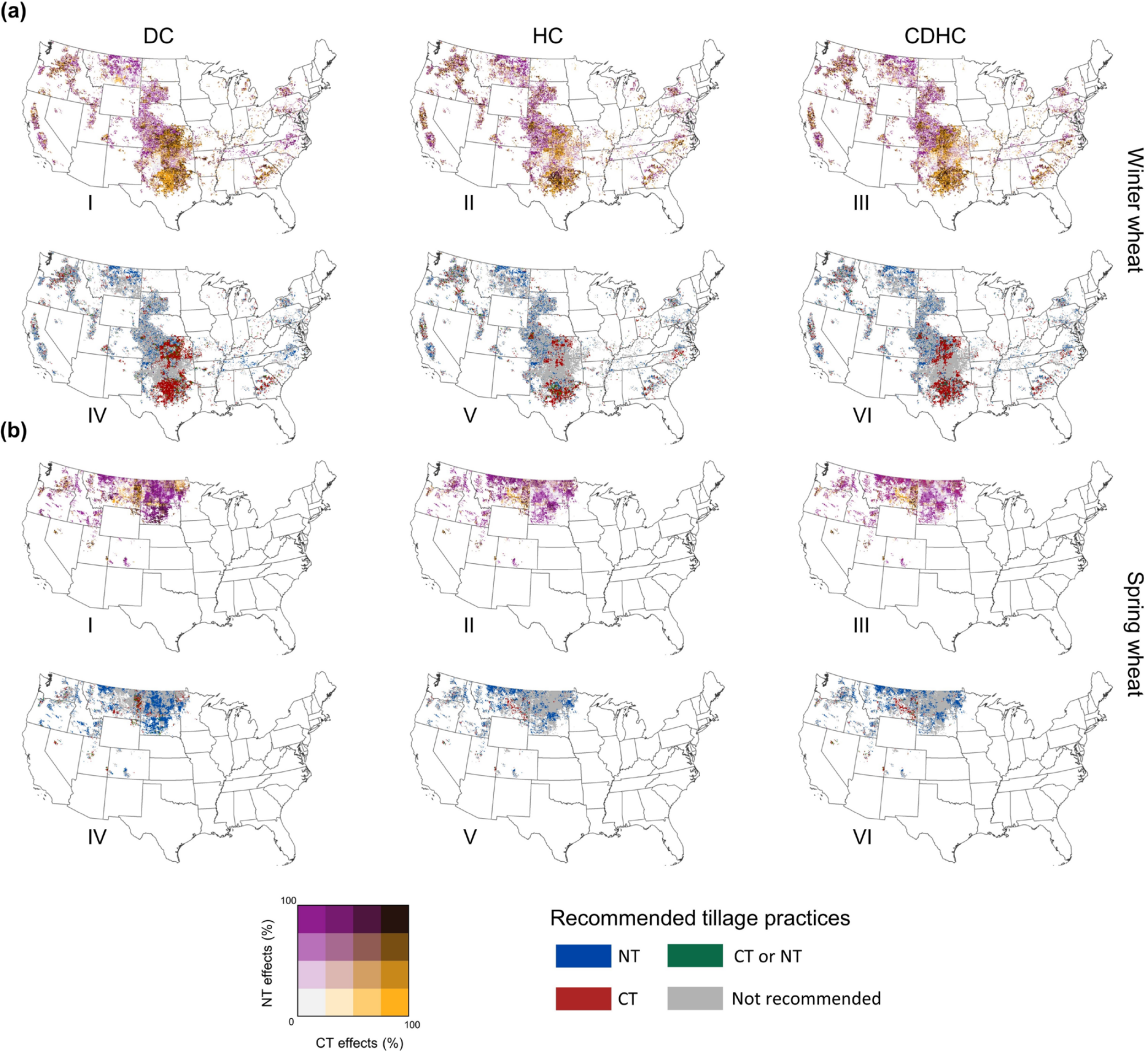
Tillage practice effects and recommended strategies for mitigating the impact of dry‐heat climates on GHG emission intensity in winter‐spring (a) and spring wheat (b). From left to right, the three columns represent dry (DC), heat (HC), and compound dry‐heat conditions (CDHC). The first (panels I–III) and second (panels IV–VI) rows of Figures a and b depict the spatial patterns of tillage effects and recommended tillage strategies. Specifically, tillage effects are expressed as the probability of reducing the negative impacts of dry‐heat conditions through tillage practices, based on 20‐year sliding windows. Tillage practices with a probability greater than 75% are recommended as effective strategies for each grid. Map lines delineate study areas and do not necessarily depict accepted national boundaries.

Specifically, this tillage was designed as a choice between CT and NT, depending on the optimal mitigation effects under specific historical and location‐based dry‐heat conditions. Our results demonstrate that both CT and NT had comparable effects in decreasing the GHGI sensitivity in the winter wheat region, whereas NT had a greater impact in the spring wheat region (Figure 7). Specifically, an analysis of 60 years of data revealed that the synergistic effects of CT and NT significantly reduced the heat sensitivity of winter wheat in the central United States. Notably, NT showed more remarkable performance in the northern region of winter wheat (Figure 7) and played a predominant role in over 90% of the spring wheat‐growing regions (Figure 7b). Tillage practices that decreased GHGI sensitivity to EDHs by over 75% were recommended as an optimal tillage scheme in specific regions, indicating the robustness of tillage strategies for decreasing climate costs under worsening future dry‐heat events. Thus, the recommended CT and NT practices were intermingled in the central region of the US, whereas NT was more highly recommended in the northern region (Figure 7). The regions implementing optimal tillage practices account for ~32.8% (27.5%–41.5%) of spring wheat‐growing regions and ~28.8% (26.7%–30.6%) of winter wheat‐growing regions across the US. Nonetheless, some areas were not included in the recommended tillage scheme, which means that potential tillage practices failed to mitigate the dry heat dependence of the GHGI in these regions, necessitating other options to bridge this gap (Figure 7).

**FIGURE 8:**
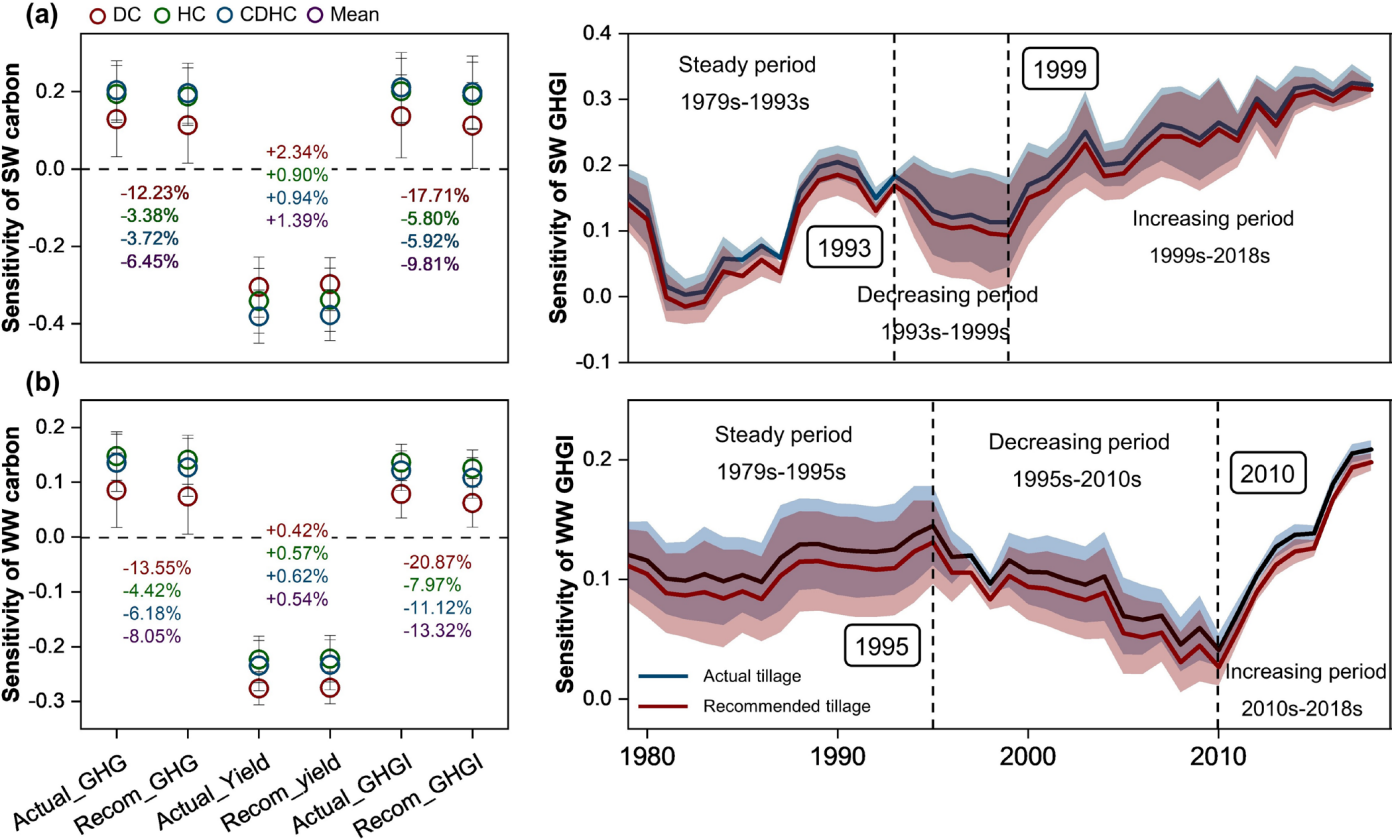
Sensitivity variations to EDH conditions based on recommended tillage strategies. Panels (a) and (b) represent winter wheat and spring wheat, respectively. The standard deviation in the left panel represents the fluctuation from 1979s to 2018s. The shaded area in the right panel is expressed as uncertainties under different dry‐heat conditions. GHG and GHGI note greenhouse gas emissions and their emission intensity, respectively. DC, HC, and CDHC represent heat, dry, and compound dry‐heat conditions, respectively.

The implementation of environment‐specific tillage schemes has demonstrated a significant reduction in the sensitivity of wheat GHGs to dry‐heat conditions, which amounted to ~6.5% for spring wheat (3.4%–12.2%) and ~8.1% for winter wheat (4.4%–13.6%), respectively, across the US (Figure 8). However, the benefits of this tillage practice are limited. Co‐alterations between GHGs and yield dependence drive further decreases in GHGI sensitivity, leading to a reduction of ~9.8% for spring wheat (5.8%–17.7%) and ~13.3% for winter wheat (8.0%–20.9%) across the US (Figure 8). Moreover, the environment‐specific tillage scheme reduced the negative impacts of EDHs across different historical periods (Figure 8).

The long‐term sustainability of tillage practices in regions with significant effects, where tillage practices continuously reduce or enhance carbon sensitivity, has been questioned. Specifically, under dry‐heat conditions, the negative effects of tillage practices deteriorated over time, whereas the positive effects gradually diminished for winter wheat GHGs and yield (Figure S21a–c). Unlike winter wheat, the dynamic changes in tillage practice effects (positive and negative) over time did not follow a consistent trend for spring wheat GHGs and yields (Figure S22a–c). If tillage schemes are not adjusted for negatively affected regions, EDHs can further exacerbate the climatic costs of winter wheat food production over time. This underscores the importance of adopting region‐specific tillage practices in specific locations to fully offset dry heat‐induced losses.

### 3.7 Uncertainty of Sensitivity Variations From Different Meteorological Datasets and Methods

We introduced two independent meteorological drivers (Livneh and ERA5) and nonlinear methods (Kendall and Spearman correlations) for a comparative analysis of sensitivity variations (Figures S23, S24, S25, and S26). Additionally, we integrated all datasets and methods to generate nine sets of sensitivity results for uncertainty analysis (Figure S27). We observed consistent spatial patterns of sensitivity across different meteorological datasets for both winter and spring wheat. Furthermore, a coherent trend was identified, showing a sharp increase in the dry‐heat sensitivity of winter wheat GHG emissions after 2008 and a continuous rise in spring wheat sensitivity over the past six decades (Figures S23, S24, S25, and S26). These results indicate that the meteorological datasets employed in this study are sufficient to capture the spatiotemporal variability of wheat sensitivity. Additionally, the various methods produced highly consistent outcomes, with nearly identical spatial distributions and temporal dynamics in sensitivity. Notably, variations in dry‐heat sensitivity across methods were independent of wheat type, demonstrating that the use of linear correlation as a sensitivity probe did not undermine result robustness, despite minor uncertainty.

Using the nine datasets, we estimated the uncertainty in the simulated dry‐heat sensitivity of spring wheat over the past 60 years as 0.08 ± 0.02, 0.16 ± 0.01, and 0.16 ± 0.01 for aridity, heat, and compound conditions, respectively (Figure S27). For winter wheat, the uncertainties were 0.09 ± 0.03, 0.15 ± 0.03, and 0.13 ± 0.03. In summary, the aggregated sensitivity results were consistent with the magnitude and trends observed in this study (Figure S27).

## 4 Discussion

### 4.1 Climate and Fertilization Dominate Yield and GHG Variations

While previous studies have extensively investigated crop productivity, relatively few have focused on the GHG emissions associated with wheat cultivation. Our study aims to bridge this gap through a model‐data integration framework that combines a process‐based agricultural model with multiple in situ measurements and nursery yield statistics. Validation with observational data demonstrated that this framework explained most of the variation in both wheat productivity and GHG emissions (Figure 1). Moreover, Figure S28 shows regional estimates of wheat productivity that closely match the spatial patterns and magnitudes reported in the USDA survey data. Wheat production hotspots were located in the northwestern US (e.g., Washington and Montana) and the central Great Plains (e.g., Kansas and Oklahoma) (Figure S28). Our estimated N_2_O emissions from US wheat soils totaled 0.04 Tg N year^−1^ from 1960 to 2018, which were comparable to previous studies. For example, Tesfaye et al. (2021) conducted global estimates of wheat N_2_O emissions and reported US wheat N_2_O emissions of ~0.02 Tg N in 2013. Similarly, Lu et al. (2022) estimated wheat N_2_O emissions from US cropland soils ranging from 0.01 to 0.02 Tg N year^−1^.

Given that methane emissions in dryland cropping systems are negligible (Figure S10), our analysis focused on how climate and nitrogen fertilizer application influence variations in N_2_O and SOC. Results confirmed that anthropogenic nitrogen input has significant effects on wheat N_2_O variation across the US (Figure 3 and S10a,b). In the DLEM, cropland N_2_O emissions are regulated by processes such as nitrification and denitrification, along with environmental stresses during crop growth, and typically respond nonlinearly to nitrogen inputs. We found that nitrogen fertilizer application rates in the US have continued to rise over the past six decades, making nitrogen fertilizer use the most important factor driving wheat N_2_O emissions (Figure S9a). This is supported by previous studies on the response of agricultural N_2_O emissions to nitrogen fertilizer application (Prosser et al. 2020; Zhang et al. 2020).

Nitrogen fertilizer application also emerged as the primary driver of SOC variations, followed by climate change (Figure S10c,d). In the DLEM, this is primarily due to the increase in aboveground and belowground biomass under fertilization, which subsequently enhances carbon inputs to the litter and organic matter pools (You et al. 2024). When carbon decomposition exceeds (or falls short of) carbon accumulation over a given period, SOC exhibits a net decrease (or increase), respectively. In the Central Great Plains, climate‐driven dry‐heat conditions appear to accelerate SOC decomposition (Figure S11), making climate the dominant factor in these regions (Figure S10c,d). Over the past 60 years, climate‐induced SOC loss has been observed in US croplands, whereas nitrogen fertilization has contributed to SOC accumulation (You et al. 2024).

Climate change was also a key factor influencing wheat yield variation, particularly for winter wheat (Figure 3c,d). This is because climate factors (e.g., solar radiation, temperature, and precipitation) affect key physiological processes such as crop photosynthesis, transpiration, phenological development, and carbon allocation, which in turn influence biomass accumulation and yield formation. Additionally, we have conducted a sensitivity analysis to identify the most influential parameters affecting wheat yield formation (You et al. 2022). The results revealed that the lower cardinal temperature for heat stress reduction in grain number, the lower optimal cardinal temperature required for photosynthesis, and the threshold of the 10‐day running average temperature for sowing are decisive factors influencing yield formation (You et al. 2022). These findings suggest that temperature‐related processes have a greater impact on modeled wheat yield than other climate drivers. Therefore, significant changes in environmental temperature are likely to generate more pronounced yield responses. This is consistent with previous findings highlighting the impact of temperature on wheat yield. For example, Asseng et al. (2017) reported that rising temperatures hinder photosynthesis by altering plant physiology and metabolism, which accelerates wheat senescence and ultimately reduces yield. Trnka et al. (2014) further showed that wheat resistance to high temperatures declines over time, suggesting a higher risk of yield reduction with future warming.

### 4.2 Dry‐Heat Events Cause Yield Losses and GHG Emissions

After experiencing EDH events, wheat production and GHG emissions in US wheat regions exhibited synergistic changes, with 81.4% of regions showing yield reductions while 79.2% experienced increased GHG emissions (calculated as the average affected area across spring and winter wheat, Figure S12). Summer dry‐heat events were the dominant factor affecting annual carbon flux variability in these areas. Water scarcity experiments have shown an increase in plant mortality under arid conditions (Phillips et al. 2010; Moser et al. 2014) and their potential effects on aboveground carbon variation (Bonal et al. 2016). Decreased chlorophyll content (Mafakheri et al. 2010), shortened grain‐filling duration (Lobell et al. 2012), and early flowering induced by dry‐heat stress lead to the premature senescence of wheat and even crop failure. These dry‐heat stresses usually lead to decreased carbon input to soils and thus prevent SOC accumulation. Dry‐heat stress also stimulates ecosystem respiration rate and litter turnover, which directly influences CO_2_ and N_2_O emissions (Chaves et al. 2002; O'Connell et al. 2018). During short‐term water deficits, both plants and soil microbes adapt metabolically, which promotes root and microbial respiration, consequently intensifying the oxidation of soil organic matter and resulting in an additional release of CO_2_ (Bista et al. 2017).

In parts of the US Great Plains, the sensitivity of winter wheat yield to EDHs has decreased over time, leading to a significant yet subtle trend (Figure 5b). This phenomenon was possibly due to increased irrigation activities in the central Great Plains (Figure S29) (Wang et al. 2021). Wang et al. (2021) reported a significant yield gap between rainfed and irrigated farming systems, indicating the need for improved irrigation infrastructure in the winter wheat planting regions. Therefore, reducing the sensitivity of wheat yield to EDHs could benefit from irrigation practices in drier areas and even help offset climate‐induced production losses. In contrast, trends in spring wheat indicated that the negative impacts of EDHs on yield will significantly increase in most spring wheat areas and are expected to suffer substantial losses in an enhanced dry‐heat climate (Figures 5 and 6). This may be explained by the fact that the flowering of spring wheat experiences higher temperature exposure than winter wheat (Zhang et al. 2022). As summer warming continues, this has likely contributed to an increasing negative sensitivity of spring wheat yield to heat over time (Tack et al. 2015).

### 4.3 Effects of Environment‐Specific Tillage on Mitigating Negative Impacts of EDH Climate

This study examined environment‐specific tillage practices, tailored to the exposure characteristics of EDHs, to analyze their potential for mitigating the sensitivity of wheat GHGI to dry‐heat stress. The effectiveness of a given tillage practice depends on the interplay among EDH exposure, environmental factors, and local management strategies. Accordingly, we observed that the effects of tillage practices varied across wheat‐growing regions, with some areas showing positive responses and others negative (Figure 7). Nevertheless, the positive effects of NT in mitigating GHGI sensitivity have been widely observed in both spring and winter wheat regions (Figure 7). These benefits may be attributed to NT's capacity to improve soil water retention, stabilize crop yields, and enhance microbial resilience under stress (Abdalla et al. 2016; Ruis et al. 2022). Our results also indicate that the positive effects of NT in reducing GHGI sensitivity may stem from its ability to reduce N_2_O emissions, particularly in spring wheat regions (Figure S19). Nitrogen mineralization in NT soils tends to occur more slowly than in tilled soils, leading to lower concentrations of NH_4_^+^ and NO_3_^−^, and, consequently, reduced N_2_O emissions (Text S3). The spatial variation in NT benefits is partly due to latitude‐related temperature gradients. Previous modeling studies have shown that the benefits of NT in reducing N_2_O emissions diminish significantly with increasing air temperatures, while precipitation has little effect (Huang et al. 2022). Soil texture is another important factor influencing NT benefits, with these benefits increasing as clay content rises (Huang et al. 2022). However, this factor may not fully explain the spatial variation in NT benefits between spring and winter wheat regions.

Our findings further revealed that CT partially mitigates the negative impacts of EDH conditions on GHGI in irrigated regions of the Central Plains (Figure 7). This is likely due to higher yields under CT than NT in irrigated regions, with the effect particularly pronounced under EDH conditions (Figures S11 and S29). The yield benefit under CT may reflect its role in incorporating surface residues and redistributing soil organic matter and nutrients, thereby enhancing nutrient availability and supporting crop growth under EDH conditions [Text S3.1; (Williams et al. 2008)]. However, tillage‐induced disturbances would also affect the soil carbon pool turnover by directly enhancing decomposition rates and indirectly altering nutrient and moisture availability (Text S3.3; Huang et al. 2020), potentially contributing to increased CO_2_ emissions (Figure S20). Their overall effects on GHGI sensitivity thus reflect a balance between these opposing processes. When selecting tillage methods, it is essential to comprehensively consider various factors such as soil type, climatic conditions, crop types, and the resources and technical expertise of farmers.

Our results indicate that optimal tillage significantly mitigated wheat GHGI sensitivity in less than one‐third of wheat‐growing regions (Figure 7). This limited effectiveness is likely due to complex interactions between tillage and local environmental factors, including differences in soil conditions, microbial communities, tillage duration and depth, and other concurrent management practices (Groffman 1985; Venterea and Stanenas 2008; Van Kessel et al. 2013). Therefore, we recommend implementing region‐specific tillage strategies to partially offset the negative effects of dry‐heat extremes. However, achieving a trade‐off between extreme dry heat shock and crop resistance cannot rely completely on tillage practices. Efforts to implement smart agricultural practices that vary case by case are needed to minimize the impact of extreme weather on crop production.

### 4.4 Limitations and Perspectives

This study has several limitations and uncertainties. First, the forcing datasets used in the model introduced uncertainties. For instance, although we reconstructed fertilizer and irrigation data based on county‐ or state‐level statistics, these datasets may not fully capture the spatial variability in the magnitude and timing of management inputs. Tillage intensity data have been available at the county level over the past few decades, maintaining high data quality but lacking detailed spatial information. Therefore, the dataset inevitably introduced extrapolation errors for earlier years. Additionally, the model assumes that the percentage of crop residue removal associated with different tillage practices introduces additional uncertainty that may vary significantly from the actual residue input into soils.

Second, the DLEM provides a simplified representation of some management practices, potentially leading to a simulation bias. For example, the current model represents the crop response to irrigation as unaffected by water stress without accounting for the irrigation amount and timing. This may lead to biases in the simulation of soil moisture, thereby influencing the prediction of GHG emissions. It is essential to acknowledge that our model structure is inherently incomplete and uncertain, resulting in overall uncertainty in the model simulations. Another limitation of the current model is its failure to account for heat stress effects on other phenological events, such as spikelet number. This simplification may lead to an underestimation of heat stress impacts on wheat yield, as heat stress during the reproductive stage can also have a significant negative effect under real‐world conditions. Consequently, we acknowledge that the model may introduce uncertainty when applied to different geographic regions, climatic conditions, and wheat varieties. Future studies could improve the model to include the impact of reproductive‐stage heat stress on parameters such as spike number and other growth metrics, thereby enhancing its predictive capacity. To this end, the accuracy and reliability of the models should be enhanced through additional field validation and testing using datasets from diverse regions. The Coupled Model Comparison Programs (CMCP) provide crucial examples for quantifying the uncertainty associated with model structures (Friedlingstein et al. 2023; Tian et al. 2024). Therefore, we also advocate initiating CMCP to decrease the uncertainty associated with the model structures.

Third, the model parameters also contribute to simulation uncertainty. Although these parameters fall within reasonable ranges as derived from past experimental and simulation studies, previous studies have only provided simplified estimates of parameter sensitivity using prior probability sampling methods. However, this approach may not accurately capture true spatial distribution. Previous studies have shown that parameters related to crop growth and development vary with changes in the underlying surface environment (Huang et al. 2019). Despite calibrating the model with parameters for the three major winter wheat varieties grown in the United States, many physiological and biochemical parameters remain constant. This constraint limits the capacity of the model to accurately simulate regional carbon and nitrogen cycles.

Finally, we did not investigate the duration of dry‐heat events, which may have failed to capture the occurrence of dry‐heat stress and duration at specific growth stages that impact wheat carbon sequestration. Additionally, we did not consider consecutive water scarcity events (e.g., a series of water scarcity events that occurred in succession without significant intervals between them); thus, the effects of post‐EDHs on crops in the following year were not examined. Although current models incorporate the mechanisms of crop carbon assimilation and allocation under dry‐heat stress, more research on model development is needed to incorporate the dynamics of yield and GHG sensitivity, and how they differ among wheat types.

Uncertainty in yield and GHG prediction will be amplified in future warming climates owing to the rapidly increasing negative impacts of EDHs on crop GHGI. Therefore, our results highlight the urgent need to consider the differences in GHGI dependence on dry heat stress among crop types and their long‐term dynamics under multiple agricultural management strategies.

## Author Contributions

**Yu Shi:** conceptualization, data curation, formal analysis, investigation, methodology, software, visualization, writing – original draft, writing – review and editing. **Shufen Pan:** conceptualization, funding acquisition, writing – review and editing. **Yongfa You:** data curation, methodology, software, writing – review and editing. **Stephen A. Prior:** writing – review and editing. **Di Tian:** writing – review and editing. **Huiqian Yu:** writing – review and editing. **Qiang Yu:** writing – review and editing. **Hanqin Tian:** conceptualization, funding acquisition, software, supervision, writing – review and editing.

## Conflicts of Interest

The authors declare no conflicts of interest.

## Supporting information


Data S1.

Table S6.

## Data Availability

All data and source code supporting the findings of this study are openly available in Zenodo at https://zenodo.org/records/15545654. Wheat production data were obtained from the USDA National Agricultural Statistics Service at https://quickstats.nass.usda.gov. Seasonal greenhouse gas observation data were obtained from the Kellogg Biological Station Long‐term Ecological Research site at https://lter.kbs.msu.edu/datatables/28. Tillage practice survey data were obtained from the National Crop Residue Management Survey at https://www.ctic.org/CRM. Wheat variety data were obtained from the National Association of Wheat Growers at https://www.wheatworld.org. The ERA5 meteorological data were obtained from the Copernicus Climate Change Service (C3S) Climate Data Store (CDS) at https://doi.org/10.24381/cds.4991cf48.
