# Supplementary material for: Extreme Dry‐Heat Climate Impacts on Greenhouse Gas Emission Intensity in Wheat Production: Insights and Mitigation Strategies
Source: Glob Chang Biol. 2025 Jul 11;31(7):e70349. doi: 10.1111/gcb.70349 (PMC12247449; doi:10.1111/gcb.70349)
Supplement: Supplementary file 1 — Data S1. [file GCB-31-e70349-s001.doc]

Supplementary Materials for

**Extreme Dry-Heat Climate Impacts on Greenhouse Gas Emission Intensity in Wheat Production: Insights and Mitigation Strategies**

Yu Shi *et al.*

*Corresponding author. Email: hanqin.tian@bc.edu

**This file includes:**

Text S1 to S4

Figures S1 to S29

Tables S1 to S5

**Supplementary texts**

## Text S1. Response of crop phenological development to environment stresses in DLEM 4.0

The Growing Degree Day (GDD) is a widely used trigger criterion for crop development in models such as CLM4.5. However, it has been shown to lead to earlier sowing dates than observed, as the GDD criterion is often met prematurely (Chen et al., 2015; Chen et al., 2018). Moreover, the simple linear accumulation of GDD based on daily mean air temperature fails to accurately represent crop development rates and underestimates responses to extreme dry-heat conditions. A recent comparison of various thermal functions has shown that nonlinear thermal functions typically outperform empirical linear thermal functions in predicting phenological development (Kumudini et al., 2014). This is likely to provide a more accurate representation of the phenological response of wheat to supra-optimal temperatures, which are anticipated to increase in frequency under future climate change. To this end, the GDD criterion was modified to the accumulated thermal time (*ATT*) from the earliest sowing date that exceeds the crop-specific threshold. And TT is calculated through a nonlinear (piece-wise linear) relationship [like APSIM, (Peng et al., 2018)]. The revised sowing trigger criteria used in DLEM v4.0 are as follows, with the terminology used in the equations defined in Table S0.

$$\left\{\begin{matrix} SDate_{earliest}\leq SDate\leq SDate_{latest} \\ T_{avg}^{p}<T_{avg}^{10d} \\ T_{min}^{p}<T_{min}^{10d} \\ ATT_{min}<ATT \end{matrix}\right.$$

where $SDate_{earliest}$ and $SDate_{latest}$ denote the crop-specific prescribed earliest and latest sowing dates, respectively, which are obtained from input data. $SDate$ denotes the simulated sowing date. $T_{avg}^{p}$ and $T_{min}^{p}$ denote the crop-specific thresholds of the 10-day running average and minimum temperatures for sowing. $T_{avg}^{10d}$ and $T_{min}^{10d}$ denote the actual 10-day running average and minimum air temperatures, respectively. $ATT_{min}$ denotes the crop-specific threshold of minimum thermal time for sowing. The accumulated thermal time from the earliest sowing date to the current day is denoted by $ATT$(Table S0):

$$ATT=\sum_{\begin{matrix} earliest & day \end{matrix}}^{\begin{matrix} current & day \end{matrix}} dailyTT$$

$$dailyTT=\left\{\begin{matrix} \begin{matrix} 0, & \begin{matrix} if & \begin{matrix} T_{avg}\leq Card_{min} & \begin{matrix} or & T_{avg}\geq Card_{max} \end{matrix} \end{matrix} \end{matrix} \end{matrix} \\ \begin{matrix} T_{avg}-Card_{min}, & \begin{matrix} if & Card_{min}<T_{avg}\leq Card_{opt1} \end{matrix} \end{matrix} \\ \begin{matrix} Card_{opt1}-Card_{min}, & if & Card_{opt1}<T_{avg}<Card_{opt2} \end{matrix} \\ \begin{matrix} \begin{matrix} \frac{\left(Card_{max}-T_{avg}\right)\times\left(Card_{opt1}-Card_{min}\right)}{Card_{max}-Card_{opt2}}, & if \end{matrix} & Card_{opt2}<T_{avg}<Card_{max} \end{matrix} \end{matrix}\right.$$

where $dailyTT$ is the daily thermal time; $T_{avg}$ is the average air temperature; $Card_{min}$, $Card_{opt1}$ $Card_{opt2}$, and $Card_{max}$ are the crop-specific minimum, lower optimal, upper optimal, and maximum air temperatures required for photosynthesis, respectively.

Various environmental stresses influence crop daily development rates, thereby governing the initiation and duration of subsequent phenological stages (e.g., from emergence to maturity). Consequently, a Biological Days (BD)-based framework is adopted to represent crop growth under multiple environmental stressors. Specifically, daily BD is first calculated using a 3-segment temperature response function that accounts for vernalization, photoperiod effects, and environmental stresses (Eq. 4). Subsequently, the fraction of Cumulative Biological Days ($fCBD$), which represents the cumulative crop development rate updated at a daily time-step, is computed by dividing the actual accumulated BD from germination to the current day by the total BD required for maturity (Eq. 5). A phenological stage is predicted to occur when the calculated $fCBD$ reaches the corresponding target value for that stage. The definitions of the terminology used in the equations can be found in Table S0.

$$dailyBD=tempfun\times ppfun\times verfun\times f_{stress}$$

$$fCBD=\frac{\sum_{germination}^{\begin{matrix} current & day \end{matrix}} dailyBD}{\sum_{germination}^{maturity} dailyBD}$$

where $dailyBD$ denotes daily crop development rate; $tempfun$, $ppfun$, $verfun$, and $f_{stress}$ denote the inhibition of the potential crop development rate by temperature, photoperiod, vernalization, and environmental stresses, respectively. $fCBD$ denotes the fraction of accumulated$dailyBD$ to total BD required for maturity, in which $fCBD$ is equal to 0 at the germination stage and equal to 1 at the maturity stage. The crop is harvested either immediately after reaching maturity or when the growing season exceeds the crop-specific maximum duration of growth.

The effects of temperature ($tempfun$), photoperiod ($ppfun$), vernalization ($verfun$), and environmental stresses ($f_{stress}$) on crop development rate are calculated as:

$$tempfun=\frac{dailyTT}{Card_{opt1}-Card_{min}}$$

$$ppfun=\left\{\begin{matrix} \left\{\begin{matrix} \begin{matrix} 1-ppsen\times\left(CPP-PP\right), & if\text{ }PP<CPP \end{matrix} \\ \begin{matrix} 1, & if\text{ }PP\geq CPP \end{matrix} \end{matrix}\right.\text{ }(\text{for long day crop}) \\ \left\{\begin{matrix} \begin{matrix} 1, & if\text{ }PP<CPP \end{matrix} \\ \begin{matrix} 1-ppsen\times\left(PP-CPP\right), & if\text{ }PP\geq CPP \end{matrix} \end{matrix}\right.\text{ (for short day crop)} \end{matrix}\right.$$

$$verfun=\left\{\begin{matrix} \begin{matrix} 1-vsen\times\left(VDSAT-CUMVER_{i}\right), & if\text{ }CUMVER_{i}<VDSAT \end{matrix} \\ \begin{matrix} 1, & if\text{ }CUMVER_{i}\geq VDSAT \end{matrix} \end{matrix}\right.$$

$$CUMVER_{i}=\left\{\begin{matrix} \begin{matrix} CUMVER_{i-1}+VERDAY-0.5\times\left(T_{\max}-30\right), & if\text{ }CUMVER_{i-1}<10\text{ }and\text{ }T_{max}>30 \end{matrix} \\ \begin{matrix} CUMVER_{i-1}+VERDAY, & other\text{ }conditions \end{matrix} \end{matrix}\right.$$

$$VERDAY=\left\{\begin{matrix} \begin{matrix} 0, & if\text{ }T_{avg}\leq Ver_{min}\text{ }or\text{ }T_{avg}\geq Ver_{max} \end{matrix} \\ \begin{matrix} \frac{T_{avg}-Ver_{min}}{Ver_{opt1}-Ver_{min}}, & if\text{ }Ver_{min}<T_{avg}\leq Ver_{opt1} \end{matrix} \\ \begin{matrix} 1, & if\text{ }Ver_{opt1}<T_{avg}<Ver \end{matrix} \\ \begin{matrix} \frac{Ver_{max}-T_{avg}}{Ver_{max}-Ver_{opt2}}, & if\text{ }Ver_{opt2}<T_{avg}<Ver_{max} \end{matrix} \end{matrix}\right.$$

$$fstress=\left\{\begin{matrix} \begin{matrix} \begin{matrix} \begin{matrix} max\left(fW,f_{W}^{min}\right), & if\text{ }sowing\leq stage\leq end\text{ }juvenile \end{matrix} \\ \begin{matrix} min\left(fW,max\left(fN,f_{N}^{min}\right)\right), & if\text{ }end\text{ }juvenile\leq stage\leq flowering \end{matrix} \end{matrix} & \end{matrix} \\ \begin{matrix} 1, & for\text{ }other\text{ }stages \end{matrix} \end{matrix}\right.$$

where $dailyTT$denotes the daily thermal time, which is calculated using Eq. (3); $Card_{min}$ and $Card_{opt1}$denote the minimum and lower optimal air temperatures required for photosynthesis, respectively; $ppsen$ is a cultivar-specific photoperiod sensitivity coefficient; $PP$ is daylength; $CPP$ is a cultivar-specific critical daylength, beyond which the rate of phenological development becomes restricted by daylength; $vsen$ is a cultivar-specific vernalization sensitivity coefficient; $VDSAT$ is the number of vernalization days needed to saturate the vernalization response; $CUMVER$ denotes cumulative vernalization days; $VERDAY$ denotes vernalization day, representing the contribution of each day to vernalization; $T_{max}$ denotes the maximum air temperature; $Ver_{min}$, $Ver_{opt1}$, $Ver_{opt2}$, and $Ver_{max}$ are the minimum, lower optimal, upper optimal, and maximum air temperatures required for vernalization, respectively; $fW$ and $fN$ denote drought and N stresses, respectively, which are calculated using Eq. (13 and 14); and $f_{W}^{min}$ and $f_{N}^{min}$ denote the minimum drought and N stresses, respectively (here set to be 0.5).

In DLEM v4.0, the devernalization process is also incorporated when winter crops are exposed to high temperatures, namely, if $CUMVER$ is less than 10 days and the maximum air temperature is higher than 30 ℃, then $CUMVER$ is decreased by 0.5 days per degree above 30 ℃; however, if $CUMVER$ is larger than 10 days, no devernalization will occur.

## Text S2. Response of carbon allocation strategy to environment stresses in DLEM 4.0

The effects of light ($fL$), nitrogen ($fN$), and water ($fW$) stresses on the carbon allocation process are calculated as (Table S0):

$$fL=exp\left(-ext_{coef}\times LAI\right)$$

$$fN=min\left(\frac{N_{act}}{N_{max}},1\right)$$

$$fW=\sum_{i=1}^{10} froot_{i}\times\beta_{i}$$

$$\beta_{i}=\left\{\begin{matrix} \begin{matrix} 0, & if\text{ }\psi_{i}\leq\psi_{close} \end{matrix} \\ \begin{matrix} \frac{\psi_{max}-\psi_{i}}{\psi_{max}-\psi_{sat,i}}, & if\text{ }\psi_{close}<\psi_{i}<\psi_{open} \end{matrix} \\ \begin{matrix} 1, & if\text{ }\psi_{i}\geq\psi_{open} \end{matrix} \end{matrix}\right.$$

where $ext_{coef}$ denotes the canopy light extinction coefficient; *LAI* denotes the leaf area index; $N_{act}$ denotes the actual N content in the vegetation pool; $N_{max}$ denotes the maximum N content in the vegetation pool; $froot_{i}$ denotes the root fraction in the soil layer *i*; $\beta_{i}$ is a soil matric potential-related factor; $\psi_{max}$ denotes the maximum water potential, which represents the wilting point potential of leaves (currently set to be -1.5×10^5^); $\psi_{i}$ denotes the water potential of layer *i*; and $\psi_{open}$ and $\psi_{close}$ denote the water potential under which the stomata fully opens and closes, respectively.

The actual kernel weight at physiological maturity (*AKW*) is calculated as the product of daily crop development rate (*dailyBD*) and the potential kernel growth rate (*pKGR*), as well as heat and N stresses (Table S0):

$$AKW=dailyBD\times pKGB\times fN\times fheat$$

$$pKGR=\frac{pKW}{\sum_{start\text{ }grain\text{ }fill}^{end\text{ }gra\mathrm{in}\text{ }fill} dailyBD}$$

$$fheat=\left\{\begin{matrix} \begin{matrix} 1, & if\text{ }T_{avg}\leq HeatTemp_{min} \end{matrix} \\ \begin{matrix} 1-\frac{T_{avg}-HeatTemp_{min}}{HeatTemp_{max}-HeatTemp_{min}}, & if\text{ }HeatTemp_{min}<T_{avg}\leq HeatTemp_{max} \end{matrix} \\ \begin{matrix} 0, & if\text{ }T_{avg}>HeatTemp_{max} \end{matrix} \end{matrix}\right.$$

where $fN$ and $fheat$ denote the N and heat stresses, respectively; $pKW$ denotes the potential kernel weight, which is estimated as the ratio of potential kernel weight to the target *BD* during the grain filling period; $HeatTemp_{min}$ and $HeatTemp_{max}$ denote the minimum and maximum cardinal temperatures at which heat stress occurs.

## Text S3. Tillage practice in DLEM 4.0

### Text S3.1. Effects of tillage implement on soil organic matter and nutrients contents

The impact of tillage practices on litter pools involves both the incorporation of surface residues into the soil and the redistribution of SOM and nutrients across the tilled soil layers. In DLEM v4.0, litter pool can be classified into two categories: aboveground litter pool ($Litter_{ag}$) and belowground litter pool ($Litter_{bg}$). Both of the dead shoot biomass of crops due to turnover and the crop residues not removed from the field are directly added to $Litter_{ag}$, and the dead root biomass as well as the root residue are added to the $Litter_{bg}$. Besides, part of $Litter_{ag}$ will be transferred to $Litter_{bg}$ through bioturbation and tillage mixing practice (Lutz et al., 2019). For the bioturbation pathway, we assumed that 0.1897% of the $Litter_{ag}$ is transferred to $Litter_{bg}$ per day to account for the vertical displacement of litter ($f_{mix}$) under no-tillage and natural vegetation conditions (Lutz et al., 2019). Definitions of the terms are provided in Table S0. For the tillage pathway, the amount of transfer depends on tillage intensity:

$$Litter_{bg,t+1}=Litter_{bg,t}+Litter_{ag,t}\times f_{mix}$$

$$Litter_{ag,t+1}=Litter_{ag,t}\times\left(1-EF_{mix}\right)$$

where $Litter_{bg,t+1}$ and $Litter_{ag,t+1}$ denote the belowground and aboveground litter pools in the (*t* +1)th day, respectively; $Litter_{bg,t}$ and $Litter_{ag,t}$ denote the belowground and aboveground litter pools in the *t*th day, respectively; and $EF_{mix}$ denotes the mixing efficiency, with a value between 0 and 1.

The redistribution of SOM and nutrients within the tilled soil layers is determined using the methods outlined in the Agricultural Policy Environmental EXtender (APEX) model (Williams et al., 2008):

$$X_{l}=X_{ol}\times\left(1-EF_{mix}\right)+\frac{Z_{l}-Z_{l-1}}{D_{t}}\times EF_{mix}\times\sum_{k=1}^{M} X_{ok}$$

where $X_{l}$ is the amount of SOM/nutrients in layer *l* after mixing; $X_{ol}$ is the original amount of SOM/nutrients in layer *l* before mixing; $Z$ is the depth to the bottom of the tilled layer; $D_{t}$ is the tillage depth; *M* is the total number of soil layers affected by tillage operation; and $X_{ok}$ is the original amount of SOM/nutrients in layer *k* before mixing.

### Text S3.2. Effects of tillage implement on soil water processes

The impacts of tillage operations on soil water processes in DLEM v4.0 are primarily reflected in two aspects: (1) alterations in litter interception resulting from reduced surface residue coverage, which affect litter evaporation, soil evaporation, infiltration, and soil moisture content; (2) changes in soil bulk density due to tillage mixing, which in turn influence soil moisture content at saturation and field capacity.

In DLEM, precipitation and irrigation either get intercepted by the crop canopy and surface litter or reach the ground as throughfall, from where they are lost through processes such as evapotranspiration, soil infiltration, and surface runoff. The calculation of crop canopy interception follows the same method as in the natural vegetation module of DLEM, where it is determined by the minimum value between the input water content and the canopy’s water holding capacity (Tian et al., 2010). Litter interception is determined as the balance of available input water content after canopy interception and actual water holding capacity of surface litter ($whc_{lit,act}$), in which $whc_{lit,act}$ is calculated as:

$$whc_{lit,act}=whc_{lit,\max}\times f_{lit}$$

where $whc_{lit,\max}$ denotes the maximum water holding capacity of surface litter, which is obtained by multiplying $Litter_{ag}$ with a conversion factor of 2 ×10^-3^mm kg^-1^, following Lutz et al. (2019) and Enrique et al. (1999); and $f_{lit}$ denotes the fraction of soil surface covered by litter, which is calculated through adapting the equation from Gregory (1982):

$$f_{lit}=1-e^{-Am\times Litter_{ag}}$$

where *Am* denotes the area covered per dry matter of surface litter and is set to 0.004 in DLEM (Dadoun, 1993).

The calculation of litter evaporation ($EVAP_{lit}$) is similar to the calculation of soil evaporation ($EVAP_{soil}$) in DLEM, which is obtained by multiplying the potential evaporation (*PET*) estimated from the Penman–Monteith equation with a LAI-adjusted item (Pan et al., 2015; Pan et al., 2020). Here, *f_lit_* is also included in the calculation process of $EVAP_{lit}$ and $EVAP_{soil}$ to account for the impacts of changes in surface litter coverage on evaporation:

$$EVAP_{lit}=PET_{lit}\times e^{-0.6\times LAI}\times f_{lit}$$

$$EVAP_{soil}=PET_{soil}\times e^{-0.6\times LAI}\times\left(1-f_{lit}\right)$$

Tillage practices typically reduce bulk density by incorporating surface residues into the soil and promoting soil fragmentation (Guérif et al., 2001; Maharjan et al., 2018), which subsequently leads to changes in soil moisture content at saturation and field capacity. Here, the impacts of tillage implement on bulk density and the subsequent soil moisture effects are calculated as (Lutz et al., 2019):

$$f_{BDtill,l,aft}=f_{BDtill,l,pri}-\left(f_{BDtill,l,pri}-0.667\right)\times EF_{mix}$$

$$\theta_{sat,l,aft}=1-\left(1-\theta_{sat,l,pri}\right)\times f_{BDtill,l,aft}$$

$$\theta_{fc,l,aft}=\theta_{fc,l,pri}-0.2\times\left(\theta_{sat,l,pri}-\theta_{sat,l,aft}\right)$$

where $f_{BDtill,l,aft}$ denotes the fraction of bulk density change after tillage in layer *l*; and $f_{BDtill,l,pri}$ denotes the density effect before tillage in layer *l*; $\theta_{sat,l,aft}$ and $\theta_{fc,l,aft}$ are the modified soil moisture content at saturation and field capacity after tillage in layer *l*; $\theta_{sat,l,pri}$ and $\theta_{fc,l,pri}$ are the original soil moisture content at saturation and field capacity before tillage in layer *l*. In DLEM v4.0, the vertical soil profile is represented by a ten-layer discretization of a 3-meter soil profile. The layer thickness increases geometrically from the top to the bottom of the profile, with successive values of 0.1 m, 0.1 m, 0.1 m, 0.2 m, 0.2 m, 0.2 m, 0.3 m, 0.4 m, 0.4 m, and 1 m. Soil water flow between layers is computed based on Darcy’s law, with the water flow rate *q* in layer *l* approximated as:

$$q_{1}=-k\left[Z_{h,l}\right]\left[\frac{\left(\Psi_{l}-\Psi_{l+1}\right)+\left(Z_{l+1}-Z_{l}\right)}{\left(Z_{l+1}-Z_{l}\right)}\right]$$

where $k\left[Z_{h,l}\right]$ is the hydraulic conductivity at the depth of the interface of two adjacent layers $Z_{h,l}$, $Z_{l}$ is the depth of soil layer *l*, and $\Psi_{l}$ is the soil matric potential. The definitions and units of all terms used in this section are provided in Table S0.

### Text S3.3. Effects of tillage implement on decomposition

In DLEM, the direct effect of tillage implement on the decomposition rate of litter pools is represented by a tillage scalar ($f_{till}$), which has a value greater than 1, indicating the promoting effect of tillage on decomposition (Huang et al., 2020). Additionally, the indirect effect of tillage implements on decomposition is considered, primarily reflected in their impact on SOM levels, nutrient availability, actual soil moisture content, and soil moisture content at saturation and field capacity. The actual decomposition rate of each litter pool ($k_{pool}$) within the tilled soil layers is calculated as (Table S0):

$$k_{pool}=k_{pot,pool}\times f\left(T{}_{soil\_dec}\right)\times f\left(w_{soil\_dec}\right)\times f\left(clay_{soil\_dec}\right)\times f\left(N_{soil\_dec}\right)\times f_{till}$$

$$f\left(T_{soil\_dec}\right)=4.89\times e^{-3.432+0.1\times T_{soil}\times\left(1-0.5\times T_{soil}/36.9\right)}$$

$$f\left(W_{soil\_dec}\right)=\left\{\begin{matrix} \frac{1-e^{-\theta/\theta_{sat}}}{1-e^{-\theta_{fc}/\theta_{sat}}},\begin{matrix} & \begin{matrix} if & \theta\leq\theta_{fc} \end{matrix} \end{matrix} \\ 1.0044-\frac{0.0044}{e^{-5\times\left(\frac{\theta/\theta_{sat}-\theta_{fc}/\theta_{sat}}{1-\theta_{fc}/\theta_{sat}}\right)}},\begin{matrix} & \begin{matrix} if & \theta\geq\theta_{fc} \end{matrix} \end{matrix} \end{matrix}\right.$$

$$f\left(clay_{soil\_dec}\right)=1-0.75\times P_{clay}/100$$

$$f\left(N_{soil\_dec}\right)=\left\{\begin{matrix} f\left(N_{mi}\right),\begin{matrix} & \begin{matrix} if & mineralization & occurs \end{matrix} \end{matrix} \\ f\left(N_{im}\right),\begin{matrix} & \begin{matrix} if & immobilization & occurs \end{matrix} \end{matrix} \end{matrix}\right.$$

$$f\left(N_{mi}\right)=\left\{\begin{matrix} 1-\frac{avn-avn_{opt}}{avn_{opt}},\begin{matrix} & \begin{matrix} if & avn>avn_{opt} \end{matrix} \end{matrix} \\ 1,\begin{matrix} & if & avn_{opt}/2\leq avn\leq avn_{opt} \end{matrix} \\ 1+\frac{0.5avn_{opt}-avn}{avn_{opt}},\begin{matrix} & if & avn\leq avn_{opt}/2 \end{matrix} \end{matrix}\right.$$

$$f\left(N_{im}\right)=avn/n_{imm}$$

$$f_{till,i}=1+f_{cm,i}$$

$$f_{cm,i}=\left\{\begin{matrix} \left(3+5\times e^{-5.5\times P_{clay}}\right)\times\frac{EF_{mix}}{EF_{mix}+e^{1-2\times EF_{mix}}},\begin{matrix} & if & i=1 \end{matrix} \\ f_{cm,i-1}\times\left(1-0.02\times\frac{\theta}{\theta_{sat}}\right),\begin{matrix} & if & i>1 \end{matrix} \end{matrix}\right.$$

where $k_{pot,pool}$ denotes the potential decomposition rate of each litter pool without limitation or stimulation;$f\left(T_{soil\_dec}\right)$, $f\left(w_{soil\_dec}\right)$, $f\left(clay_{soil\_dec}\right)$, and $f\left(N_{soil\_dec}\right)$ denote the effect of soil temperature, soil moisture, soil texture, and N on decomposition; $f_{till}$ is a tillage scalar; $T_{soil}$ is soil temperature; $\theta$, $\theta_{sat}$ and $\theta_{fc}$ denote the actual soil moisture content, soil moisture content at saturation, and soil moisture content at field capacity, respectively; $P_{clay}$ denotes the percentage of clay content; $f\left(N_{mi}\right)$ and $f\left(N_{im}\right)$ denote the effect of N availability when mineralization and immobilization occur, respectively; $avn$ and $avn_{opt}$ denote the actual and optimum available soil N, respectively; $n_{imm}$ denotes the potential N immobilization estimated by the tentative decomposition procedure; $f_{cm,i}$ denotes the cumulative effect of tillage at day *i*; $EF_{mix}$ denotes the mixing efficiency. The decomposition rate is calculated separately in each soil layer, and $f_{till}$ is only considered in the soil layers affected by tillage practice.

It is also worth noting that, in the DLEM model, the litter pool is partitioned into four components: aboveground added organic matter 1 (*AOM1_ag_*), belowground added organic matter 1 (*AOM1_bg_*), aboveground added organic matter 2 (*AOM2_ag_*), and belowground added organic matter 2 (*AOM2_bg_*). *AOM1* represents litter that is resistant to decomposition and has a longer turnover time, whereas *AOM2* consists of more labile material that decomposes readily. The initial values and tuning ranges of the potential decomposition rate for each litter pool ($k_{pot,pool}$) are inherited from the carbon decomposition module of the CENTURY model, where the $k_{pot,pool}$ for active to passive pools ranges from 0.0066 to 18.5 g C per month (converted to annual rates and allocated on a daily basis in DLEM). These values are derived from field observations, long-term experiments, and modelling experience, and represent reference decomposition rates under conditions free from external factors that either inhibit or enhance decomposition (Parton et al., 1987; Parton, 1996; Parton et al., 2005). DLEM allows users to calibrate these parameters within observationally constrained ranges to better match region-specific target variables, although such parameters are generally not adjusted between simulation runs.

## Text S4. Nitrification, Denitrification, and N_2_O emission in DLEM 4.0

### Text S4.1. Nitrification

Nitrification, a process converting ammonium into nitrate, is simulated as a function of soil temperature, moisture, ammonium content,

$$N_{nit}=k_{nit}f\left(T_{soil\_nit}\right)f\left(w_{soil\_nit}\right)av_{NH4}$$

where $k_{nit}$ is the daily potential fraction of ammonia that is converted into nitrate and nitrogen gases, which is set to be 0.1/day. $av_{NH4}$ is the available ammonia nitrogen in soil (Table S0). $f\left(T_{soil\_nit}\right)$ is the soil temperature’s effect on nitrification,

$$f\left(T_{soil\_nit}\right)=7.24\times e^{-3.432+0.168\times T_{soil}\times\left(1-0.5\times T_{soil}/36.9\right)}$$

$f\left(w_{soil\_nit}\right)$ is the soil moisture’s effect on nitrification,

$$f\left(w_{soil\_nit}\right)=min\left(\begin{matrix} 1\text{，} & -12.904\times wfp^{4}+17.651\times wfp^{3}+5.5368\times wfp^{2}+0.9975\times wfp-0.0243 \end{matrix}\right)$$

$wfp$ is the fraction of soil porosity that is filled with water,

$$wfp=\frac{\theta}{\theta_{sat}}$$

The nitrogen gas emission in nitrification process ($Ngas_{nit}$) is calculated as,

$$Ngas_{nit}=k_{np}\times f\left(T_{soil\_nit\_emi}\right)\times wfp\times N_{nit}$$

where $k_{np}$ is the proportion of N intermediates resulting in N_2_O. The effect of soil temperature on N₂O emissions from the nitrification process is summarized as follows:

$$f\left(T_{soil\_nit\_emi}\right)=e^{-0.5\left[\left(T_{soil}-34.2\right)/17.1\right]^{2}}$$

The definitions and units of all terms used in this section are provided in Table S0.

### Text S4.2. Denitrification

In DLEM, denitrification is the process that convert nitrate into three types of nitrogen gases, namely, nitric oxide, nitrous oxide, dinitrogen. Here, we focus on the dynamics of N₂O and its associated environmental stresses. The denitrification rate is calculated as,

$$N_{denit}=N_{pot,denit}\times f\left(T_{soil\_denit}\right)\times f\left(w_{soil\_denit}\right)\times f\left(c_{av_{NO3}}\right)$$

where $f\left(T_{soil\_denit}\right)$ is the soil temperature effect, same as $f\left(T_{soil\_nit}\right)$ in nitrification process (Table S0).

$f\left(w_{soil\_denit}\right)$ is the soil water effect,

$$f\left(w_{soil\_denit}\right)=min\left(\begin{matrix} 1, & 0.0116+1.36/\left(1+e^{-\frac{wfp-0.815}{0.0896}}\right) \end{matrix}\right)$$

$f\left(c_{av_{NO3}}\right)$ represents the effect of nitrate concentration ($c_{av_{NO3}}=av_{NO3}/BD_{soil}$, $BD_{soil}$ is the soil bulk density).

$$f\left(c_{av_{NO3}}\right)=min\left(\begin{matrix} 1, & 1.17c_{av_{NO3}}/(32.7+c_{av_{NO3}}) \end{matrix}\right)$$

$N_{pot,denit}$ is the potential denitrification rate, calculated as,

$$N_{pot,denit}=\left(0.151+0.015\times P_{clay}\right)\times Rh\times kden$$

where $P_{clay}$ is the percentage of clay content, $Rh$ is the soil respiration rate, $kden$ is a parameter depending on plant functional type to tune the potential denitrification rate. The amount of gas emission from denitrification is equal to denitrification rate.

$$Ngas_{denit}=N_{denit}$$

### Text S4.3. Nitrous oxide emission

Nitrous oxide (*N_2_O*) from nitrification and denitrification are,

$$N_{2}O=\left(Ngas_{nit}+Ngas_{denit}\right)\times f\left(T_{soil\_N2O\_emi}\right)\times\left(1-f\left(w_{soil\_N2O\_emi}\right)\right)\times f\left(clay_{N2O\_emi}\right)$$

is the temperature effect on N_2_O emission,

$$f\left(T_{soil\_N2O\_emi}\right)=\frac{1}{1+e^{-0.64+0.08T_{soil}}}$$

$f\left(w_{soil\_N2O\_emi}\right)$ is the soil water effect on N_2_O emission,

$$f\left(w_{soil\_N2O\_emi}\right)=min\left(\begin{matrix} 1, & 0.0116+1.36/\left(1+e^{-\frac{wfp-0.815}{0.0896}}\right) \end{matrix}\right)$$

$f\left(clay_{N2O\_emi}\right)$ is soil texture effect on N_2_O emission,

$$f\left(clay_{N2O\_emi}\right)=min\left(\begin{matrix} 1, & 1.26e^{-0.0116P_{clay}}-0.249 \end{matrix}\right)$$

Also, the definitions and units of all terms used in this section are provided in Table S0.


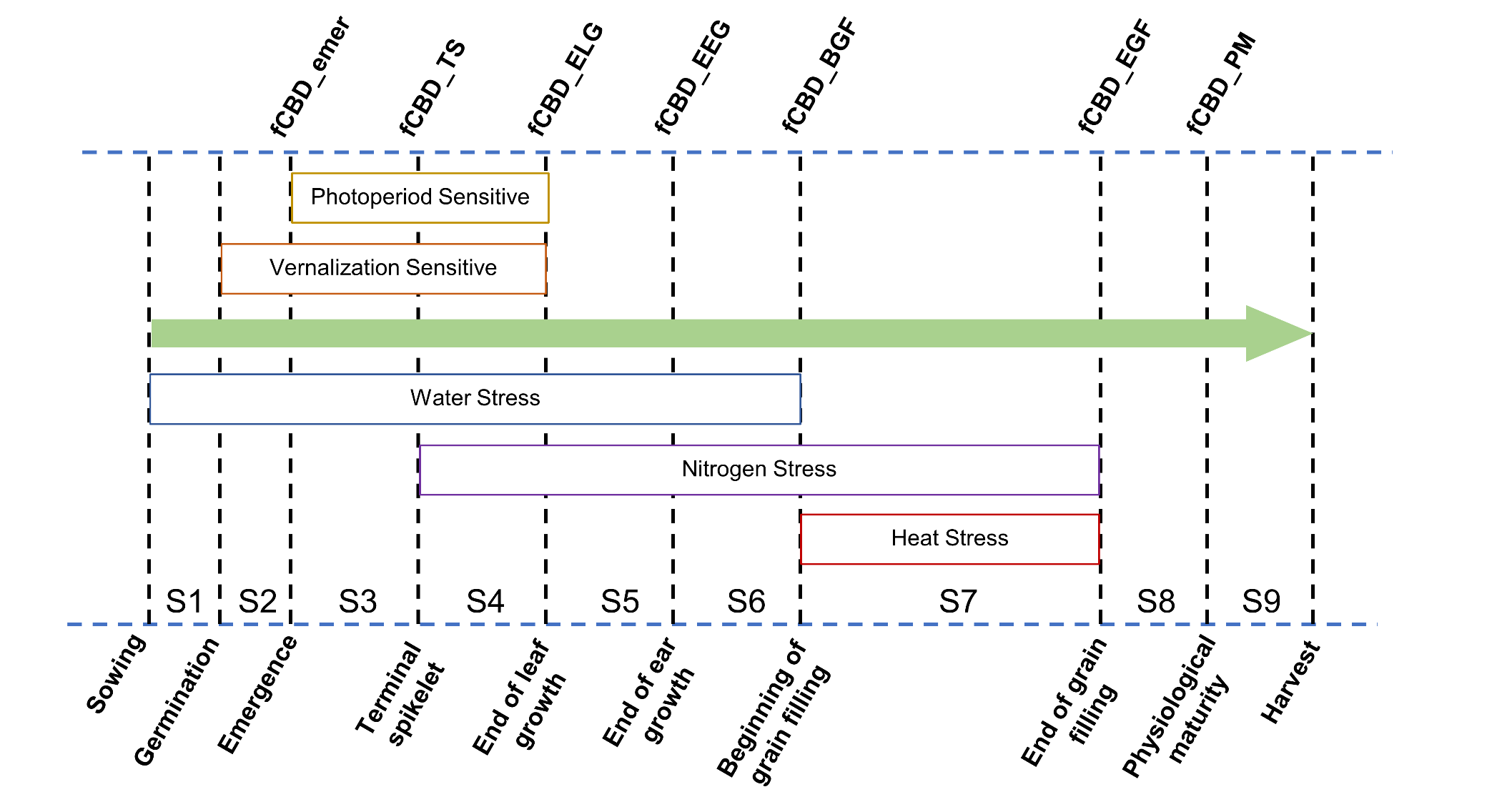


## Figure S1

**Wheat phenological development scheme in DLEM v4.0**. fCBD_emer, fCBD_TS, fCBD_ELG, fCBD_EEG, fCBD_BGF, fCBD_EGF, and fCBD_PM represent the target fraction of Cumulative Biological Days to reach the specific phenological stages.


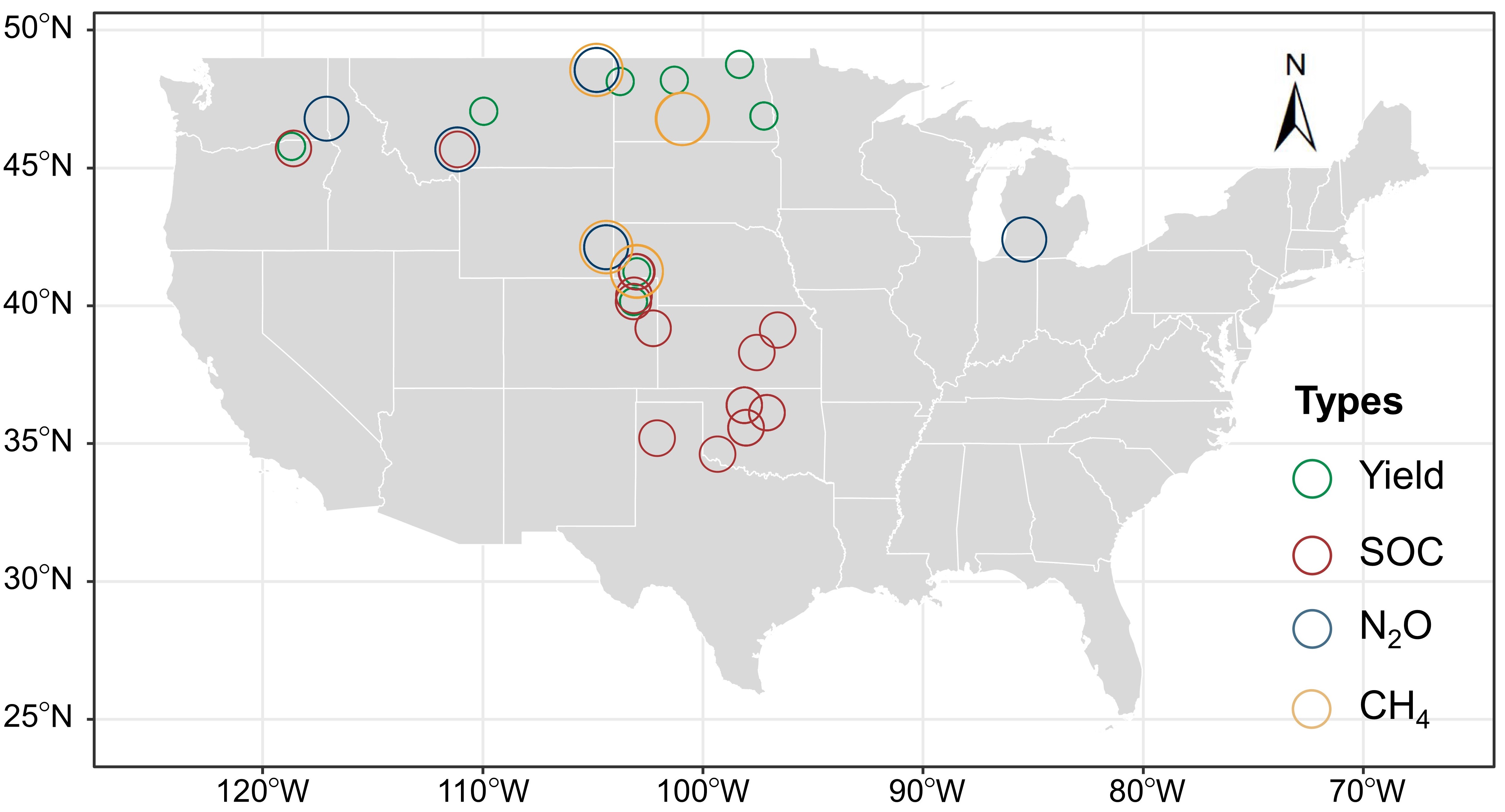


## Figure S2

**Spatial distribution of field observations for yield, soil organic carbon (SOC), nitrous oxide (N_2_O), and methane (CH_4_)**. Sites from the same location with different treatments were slightly offset to avoid overlap.


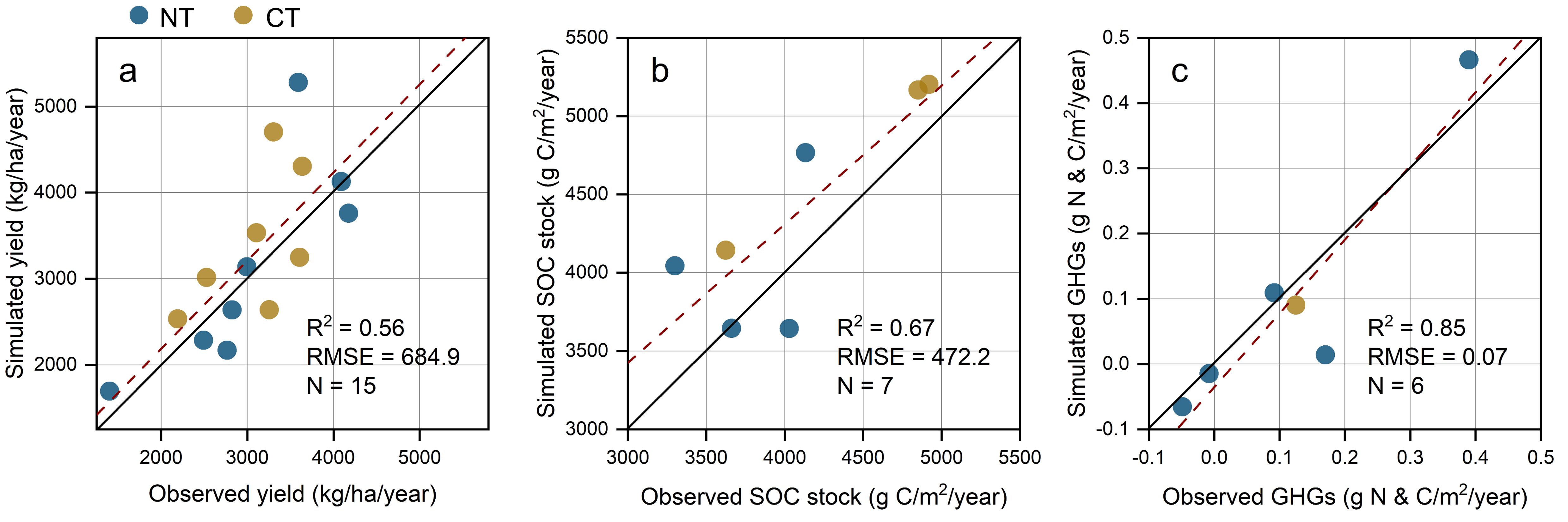


## Figure S3

**Site-scale comparisons between model estimates and field observations using the calibration dataset (20% of total observations).** Panels a–c illustrate the model performance in reproducing wheat yield, soil organic carbon (SOC) stock, and greenhouse gas emissions (CH_4_ and N_2_O) under no-tillage (NT) and conventional tillage (CT) practices. The red dashed lines and black solid lines indicate the linear regression fit and the 1:1 reference line, respectively.


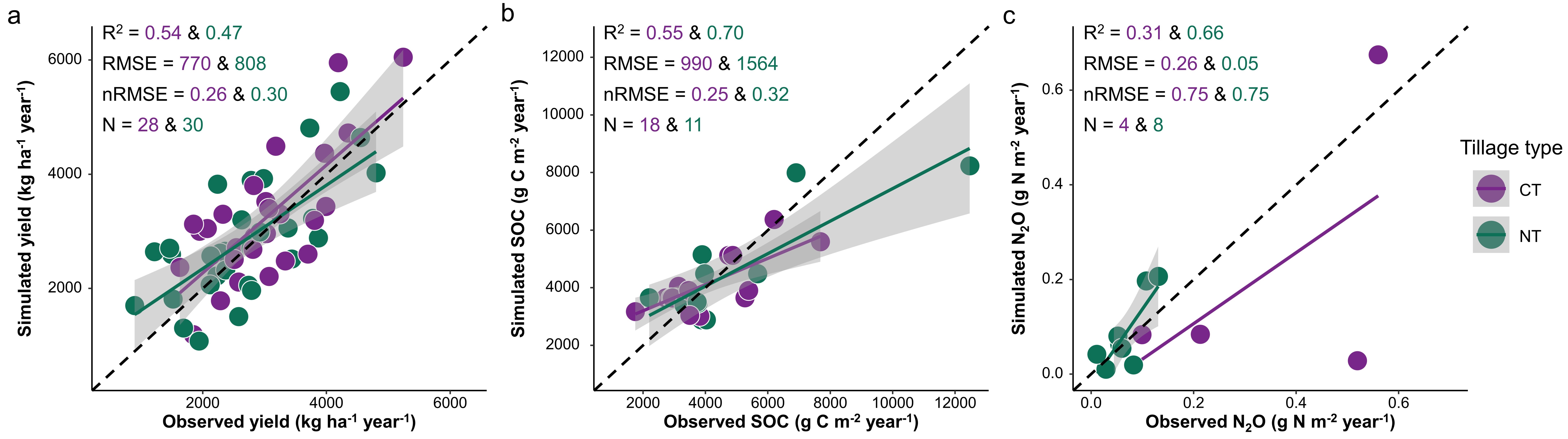


## Figure S4

**Comparison and validation of site observations and model simulations for the effect of no-tillage (NT) and conventional tillage (CT) on wheat yield (a), soil organic carbon (b, SOC), and nitrous Oxide (c, N_2_O) emissions**. The dashed and solid lines in each scatter plot represent the 1:1 line and the linear regression between observed and simulated values, respectively. Shaded bands indicate 95% confidence intervals for the mean predictions.


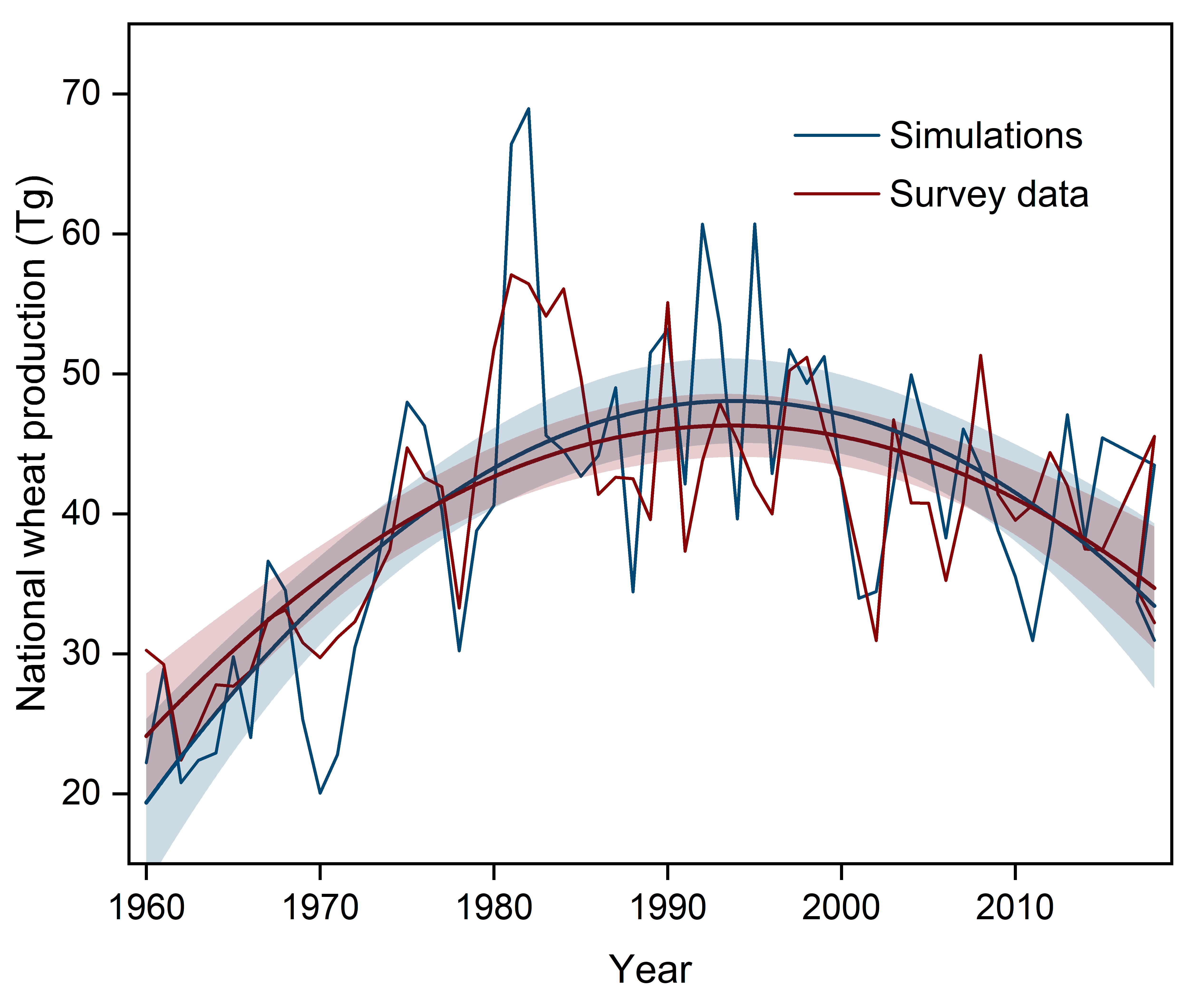


## Figure S5

**Model performance in reproducing the observed annual national wheat production**. The trend was fitted using a second-order polynomial, with the shaded band indicating 95% confidence intervals for the mean predictions.





## Figure S6

**Seasonal variations in measured and simulated GHG emissions of winter wheat at** **the Kellogg Biological Station** **Long-term Ecological Research site (42.4°N, 85.4°W)**. Measurements were conducted intermittently in 1995, 1998, 2001, 2004, 2007, 2010, and 2013. Methane (CH₄) and nitrous oxide (N₂O) emissions were quantified using permanently installed, in situ static chambers with gas chromatography. Observations are presented as means ± standard deviation from four replicates under conventional tillage treatment.

**
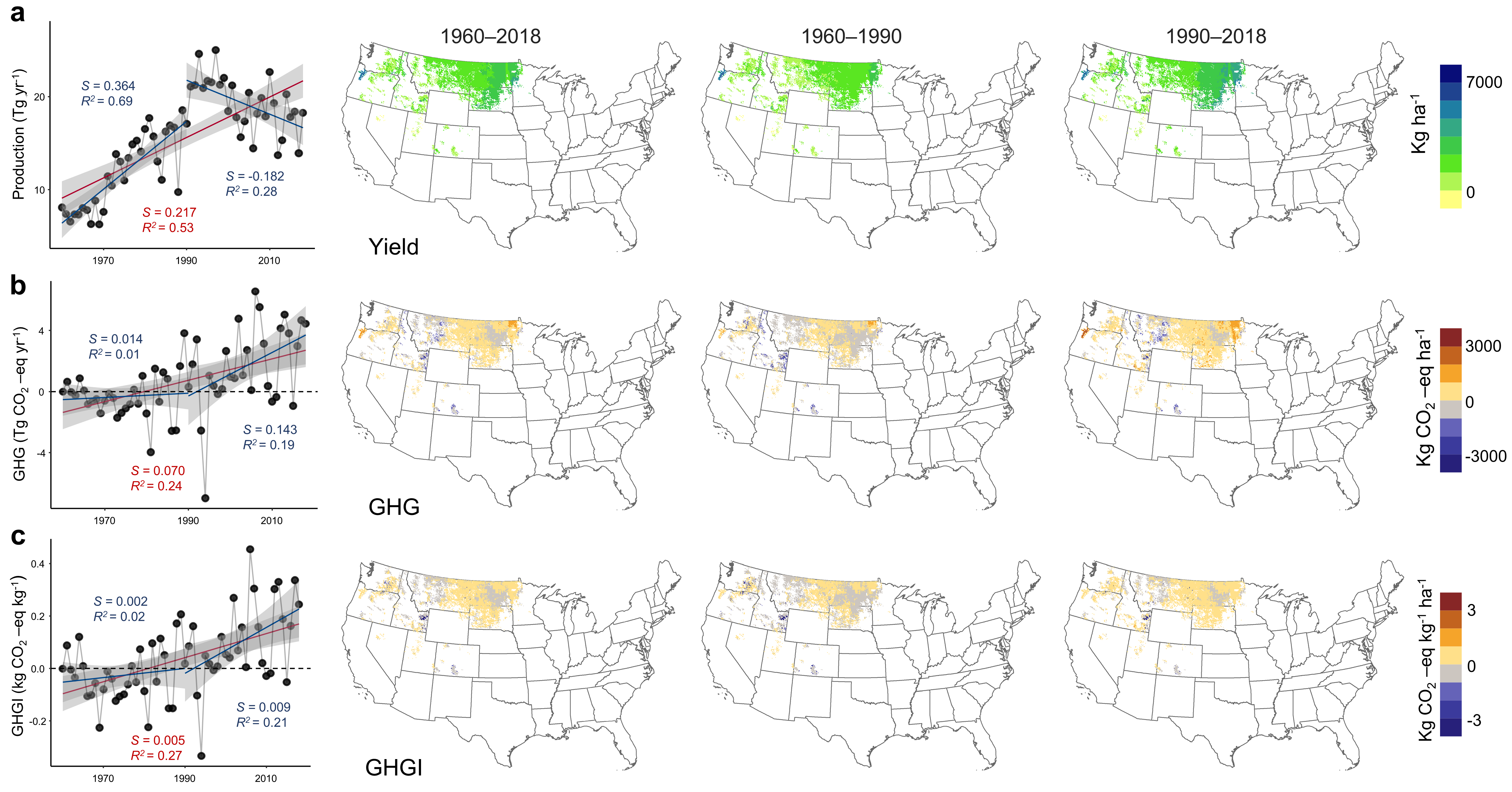
**

## Figure S7

**Spatial-temporal variations of yield, net greenhouse gases (GHG) emission, GHG emission intensity for spring wheat from 1960 to 2018 in the United States**. The simulated results are from Dynamic Land Ecosystem Model Agriculture Version 4.0. Two blue solid lines are the trend line of the two periods (1960–1990 and 1990–2018) and the red solid line is the trend line of the whole period (1960–2018) in each line graph. Shaded bands indicate 95% confidence intervals for the mean predictions. The negative value for GHG emission and GHG emission intensity represents the uptake.


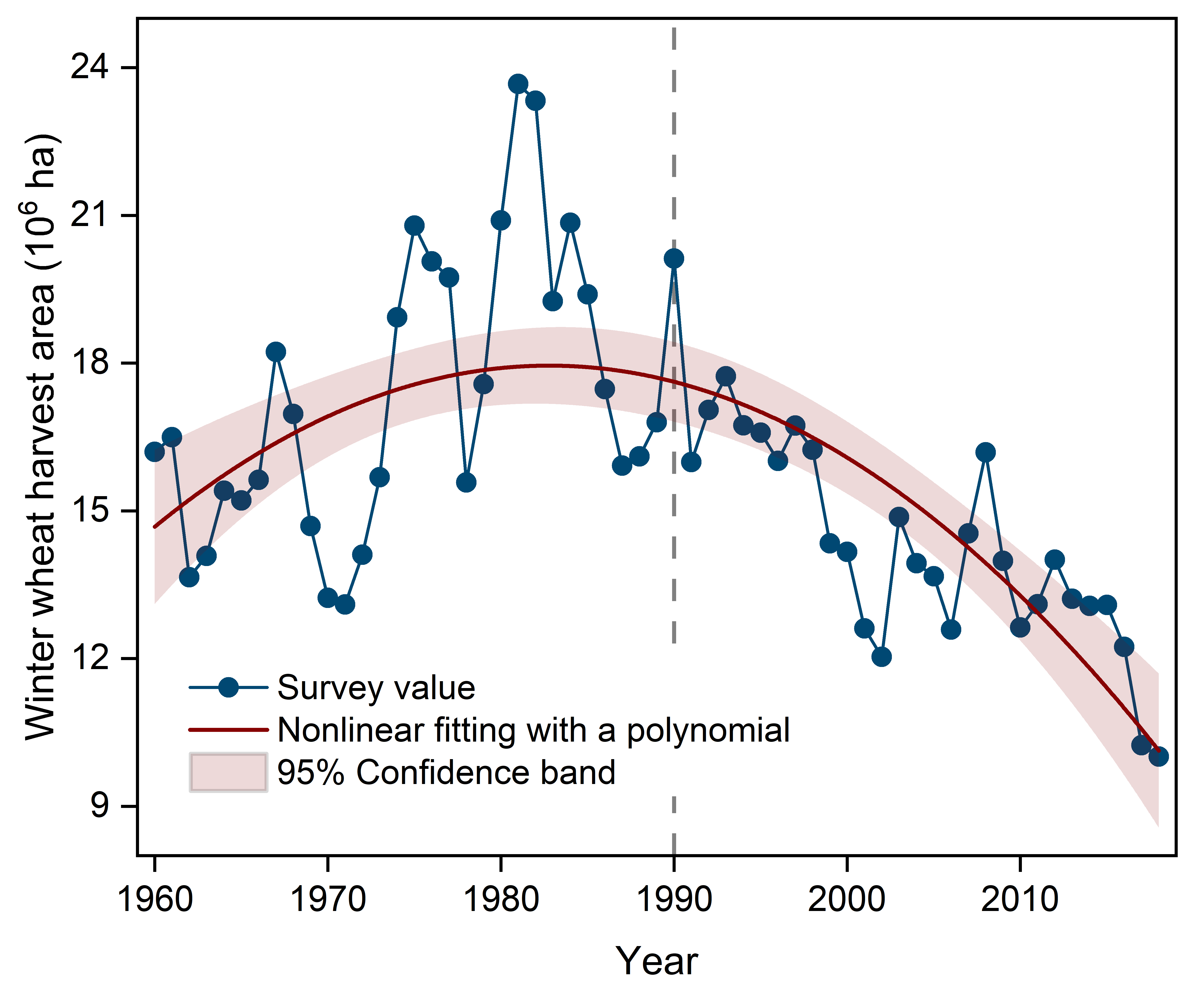


## Figure S8

**Changes in harvested area of winter wheat in the United States from 1960 to 2018.** The gray dashed line indicates the year 1990. The survey data are sourced from the USDA National Agricultural Statistics Service and were used to drive the wheat production calculations in DLEM v4.0. Shaded bands indicate 95% confidence intervals for the mean predictions


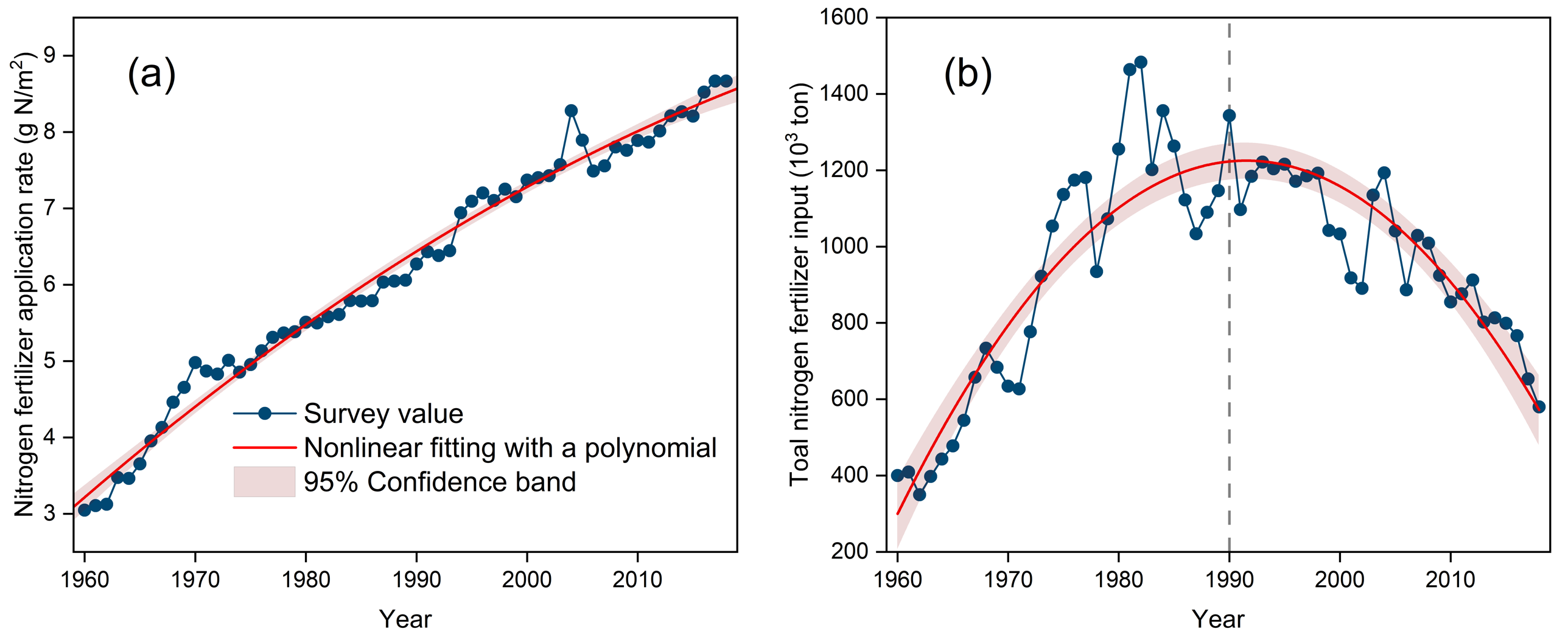


## Figure S9

**Changes in nitrogen fertilizer application rate and its total inputs for winter wheat in the United States from 1960 to 2018.** The gray dashed line indicates the year 1990. The survey data were reconstructed using state-level nitrogen use rates from the USDA-NASS and national-level commercial nitrogen consumption data from the USDA-ERS to drive the wheat simulations in DLEM v4.0. Shaded bands indicate 95% confidence intervals for the mean predictions


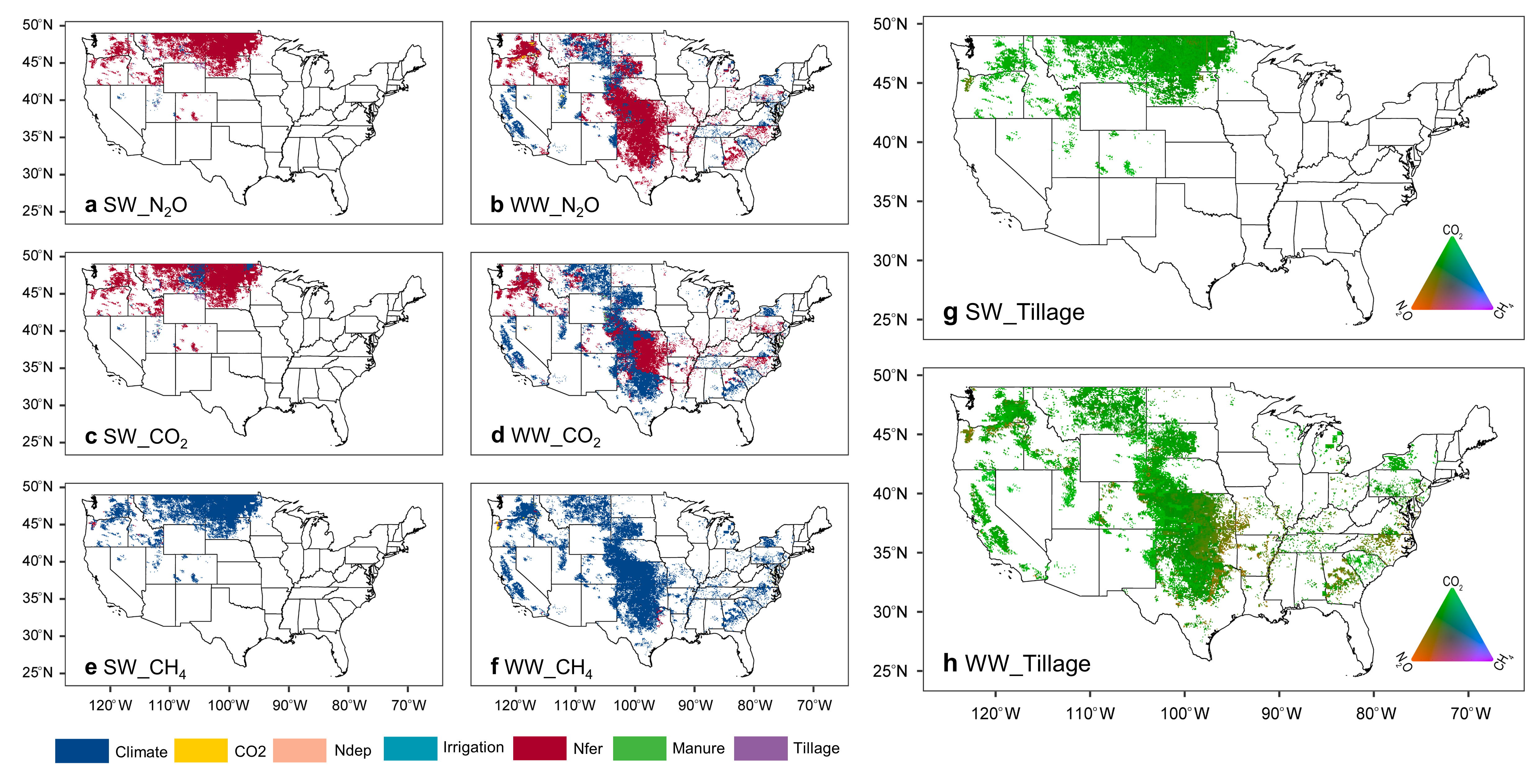


## Figure S10

**Spatial-temporal importance of multi-environmental factors to N_2_O, CO_2_, and CH_4_ for wheat in United States.** Spatial patterns of dominant factors for spring wheat (panel **a**, **c**, **and e**) and winter wheat (panel **b**, **d**, **and f**) were calculated based the period 1960–2018. Panel g and h represent the spatial impact of historical tillage practices on three greenhouse gases. Ndep: nitrogen deposition; and Nfer: Nitrogen fertilizer application; WW: winter wheat; SW: spring wheat.

**
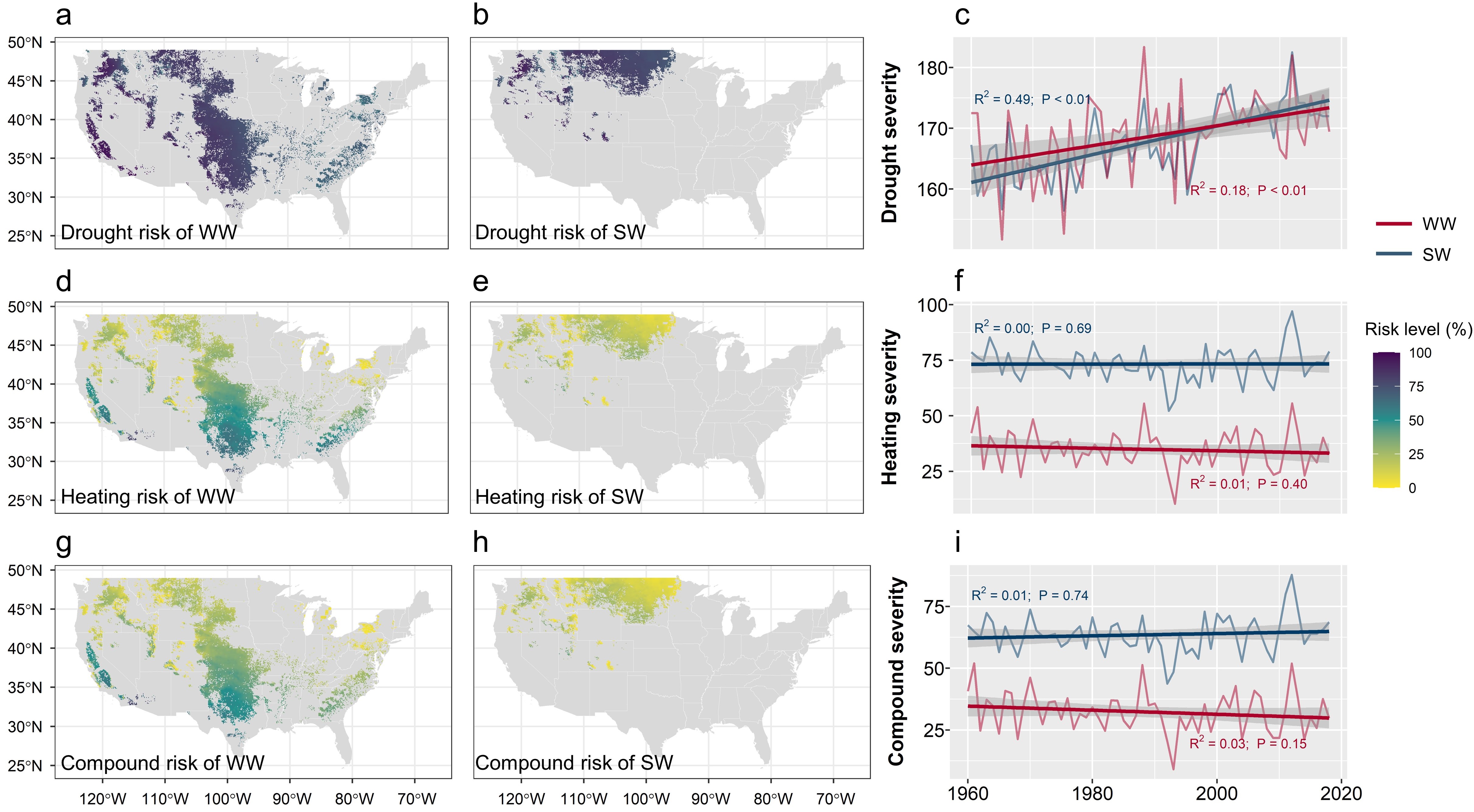
**

## Figure S11

**Risk and trends of extreme dry-heat events for US wheat over past six decades.** The ratio of occurring dry-heat-event years to total years is used as the risk level. WW and SW represent winter wheat and spring wheat. The solid line is the trend line of the whole period (1960–2018) in the panel **c**, **f**, and **i**. The shaded bands indicate 95% confidence intervals for the mean predictions.


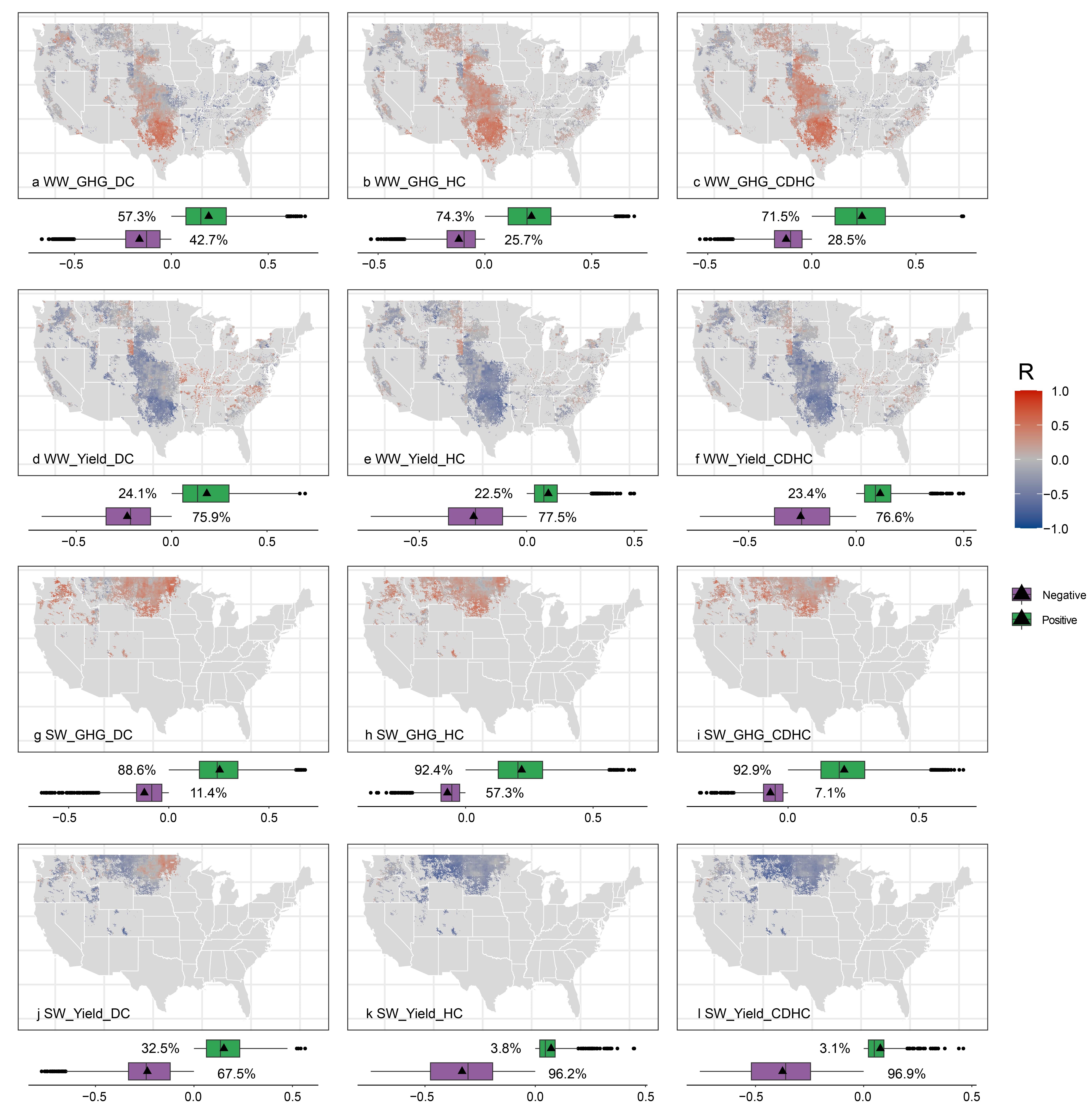


## Figure S12

**Sensitivity of wheat net greenhouse gases (GHG) emission and yield to the dry-heat events during 1960 to 2018**. Pearson correlation coefficients (R) is used to reflect the sensitivity of net GHG emission/yield to the dry-­heat events. And the red and blue bands show the positive and negative effects of dry-heats. The black triangle represents the mean value and the black points are outliers in each boxplot. WW (panel **a-f**) and SW (panel **g-l**) are winter wheat and spring wheat, respectively. DC, HC, and CDHC represent heat, dry, and compound dry-heat conditions, respectively.

**
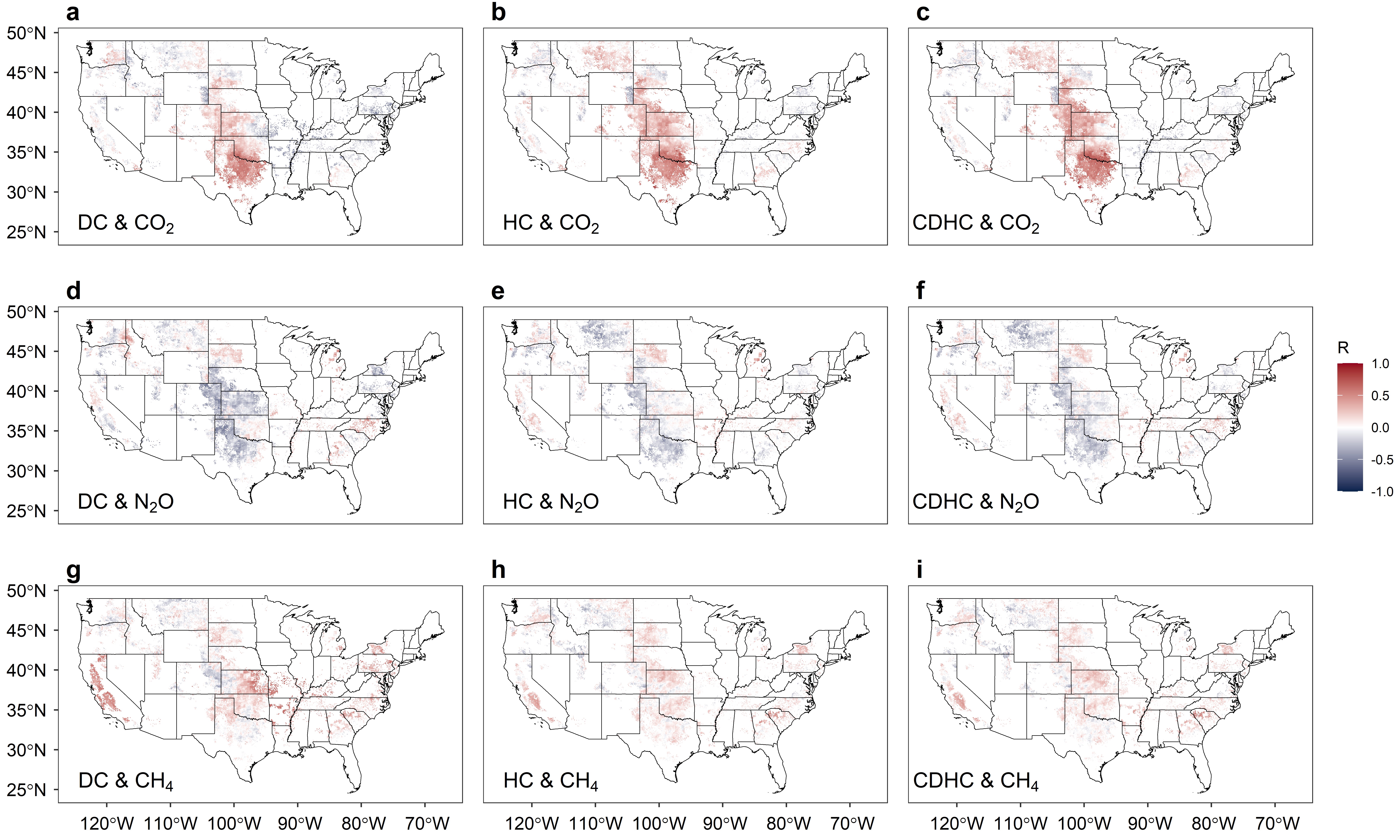
**

## Figure S13

**Sensitivity of** **individual greenhouse gas (GHG) emissions to the dry-heat events for winter wheat during 1960 to 2018**. Panels **a–c**, **d–f**, and **g–i** illustrate the sensitivities of carbon dioxide (CO_2_), nitrous oxide (N_2_O), and methane (CH_4_), respectively, to dry (DC), heat (HC), and compound dry-heat (CDHC) conditions. Sensitivity pairs are denoted using the “&” symbol (e.g., AI & CO_2_). Sensitivities are quantified using Pearson correlation coefficients (R), as detailed in Section 2.6 of the main text. The sign of R indicates the direction of the response (red for positive, blue for negative), while its magnitude reflects the sensitivity strength.

**
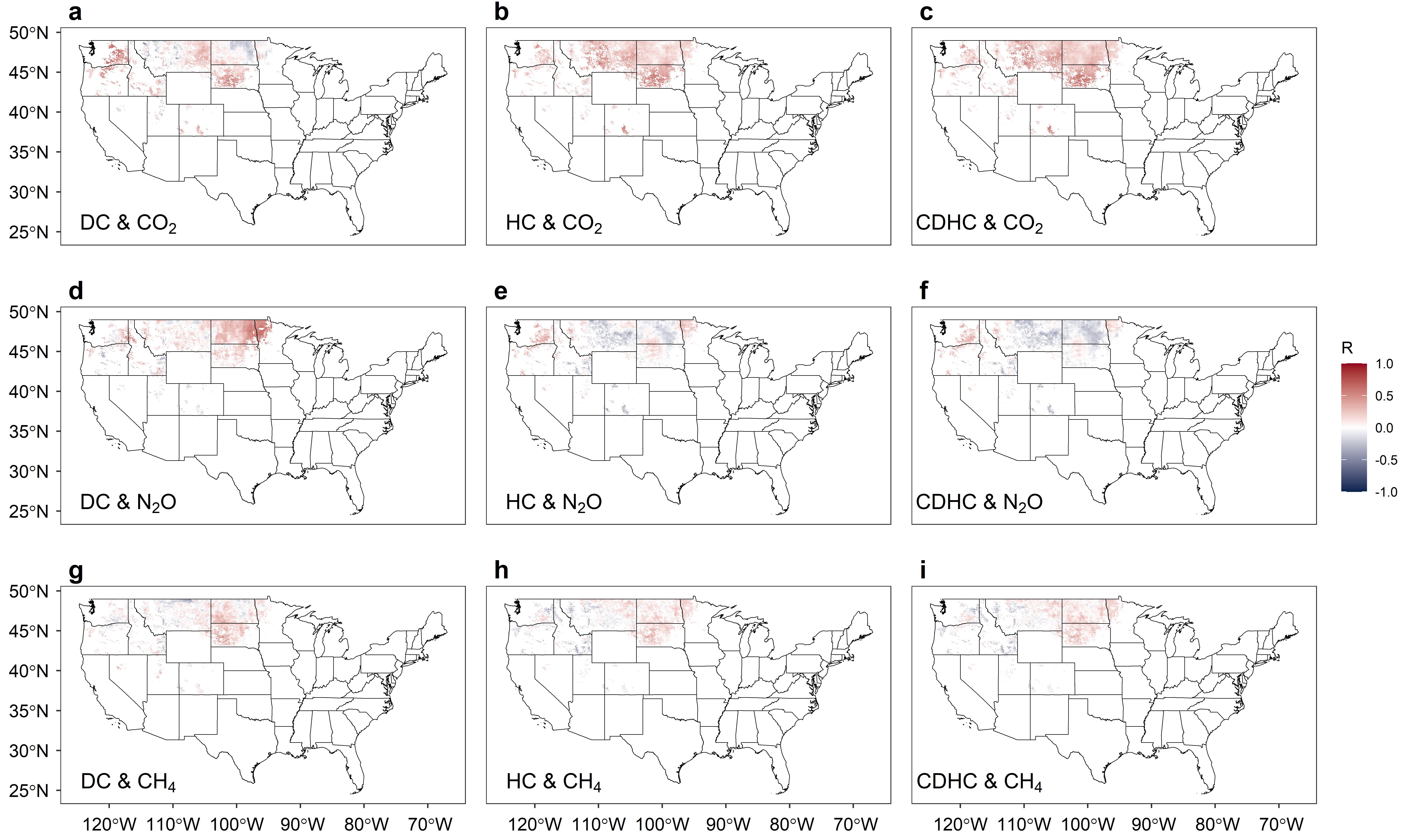
**

## Figure S14

**Sensitivity of** **individual greenhouse gas (GHG) emissions to the dry-heat events for spring wheat during 1960 to 2018**. Panels **a–c**, **d–f**, and **g–i** illustrate the sensitivities of carbon dioxide (CO_2_), nitrous oxide (N_2_O), and methane (CH_4_), respectively, to dry (DC), heat (HC), and compound dry-heat (CDHC) conditions. Sensitivity pairs are denoted using the “&” symbol (e.g., AI & CO_2_). Sensitivities are quantified using Pearson correlation coefficients (R), as detailed in Section 2.6 of the main text. The sign of R indicates the direction of the response (red for positive, blue for negative), while its magnitude reflects the sensitivity strength.


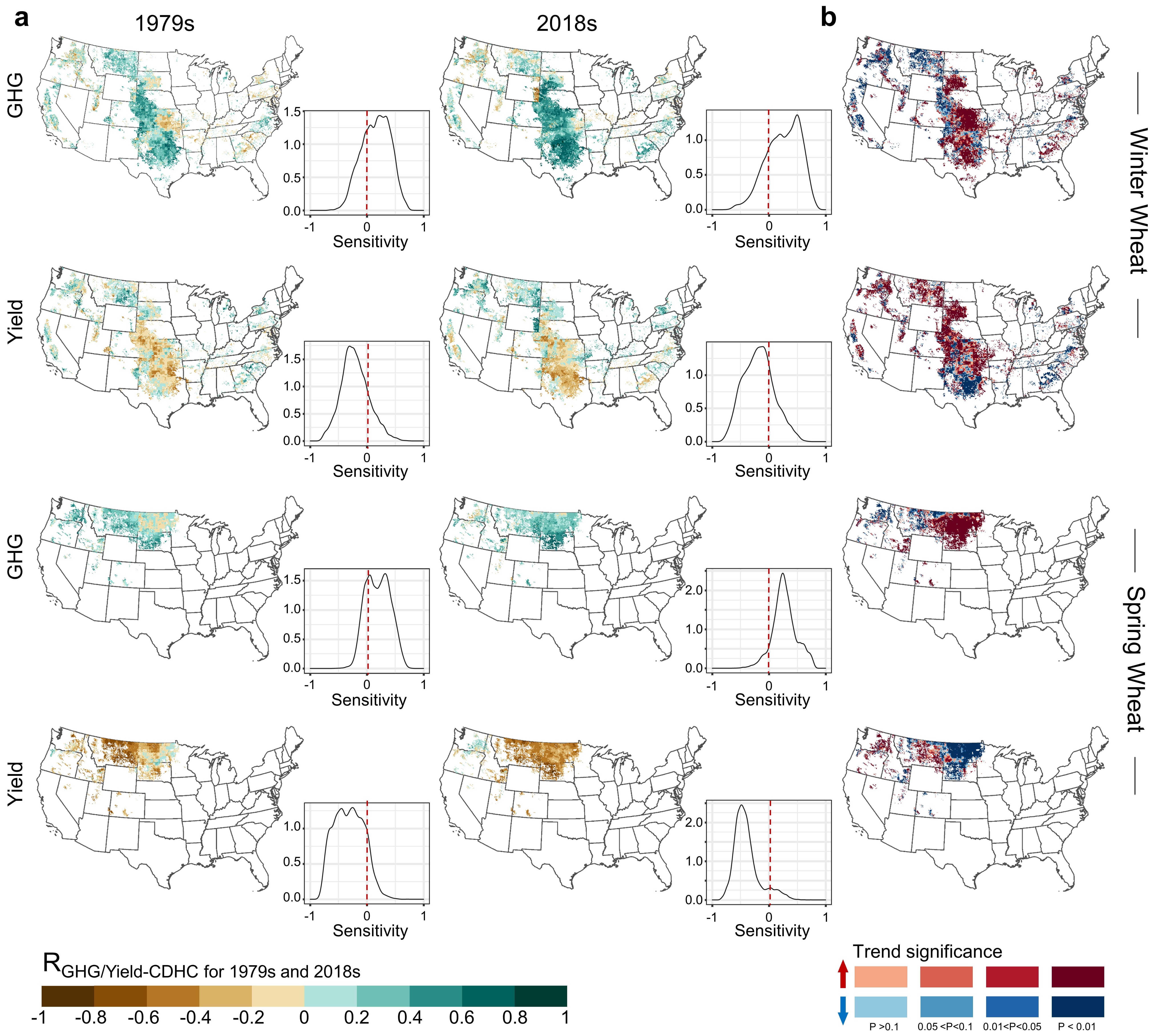


## Figure S15

**Sensitivity variations of net greenhouse gas (GHG) emissions and yield to the compound dry-heat conditions (CDHC) for wheat from the 1979s to 2018s.** A 20-year sliding window approach was used to examine the temporal variations in sensitivity to CDHC, with sensitivities within each window quantified using Pearson correlation coefficients (R), as detailed in Section 2.6 of the main text. Panel **a** shows the sensitivity maps for the first (1979s, 1960–1979) and last (2018s, 1999–2018) 20-year sliding windows. A probability density plot in the lower-left corner of each map illustrates the distribution of pixel-level sensitivities, with the horizontal axis representing R values and the vertical axis showing probability density. Panel **b** shows the linear temporal trend of sensitivity at the pixel scale, along with its statistical significance (P value).

**
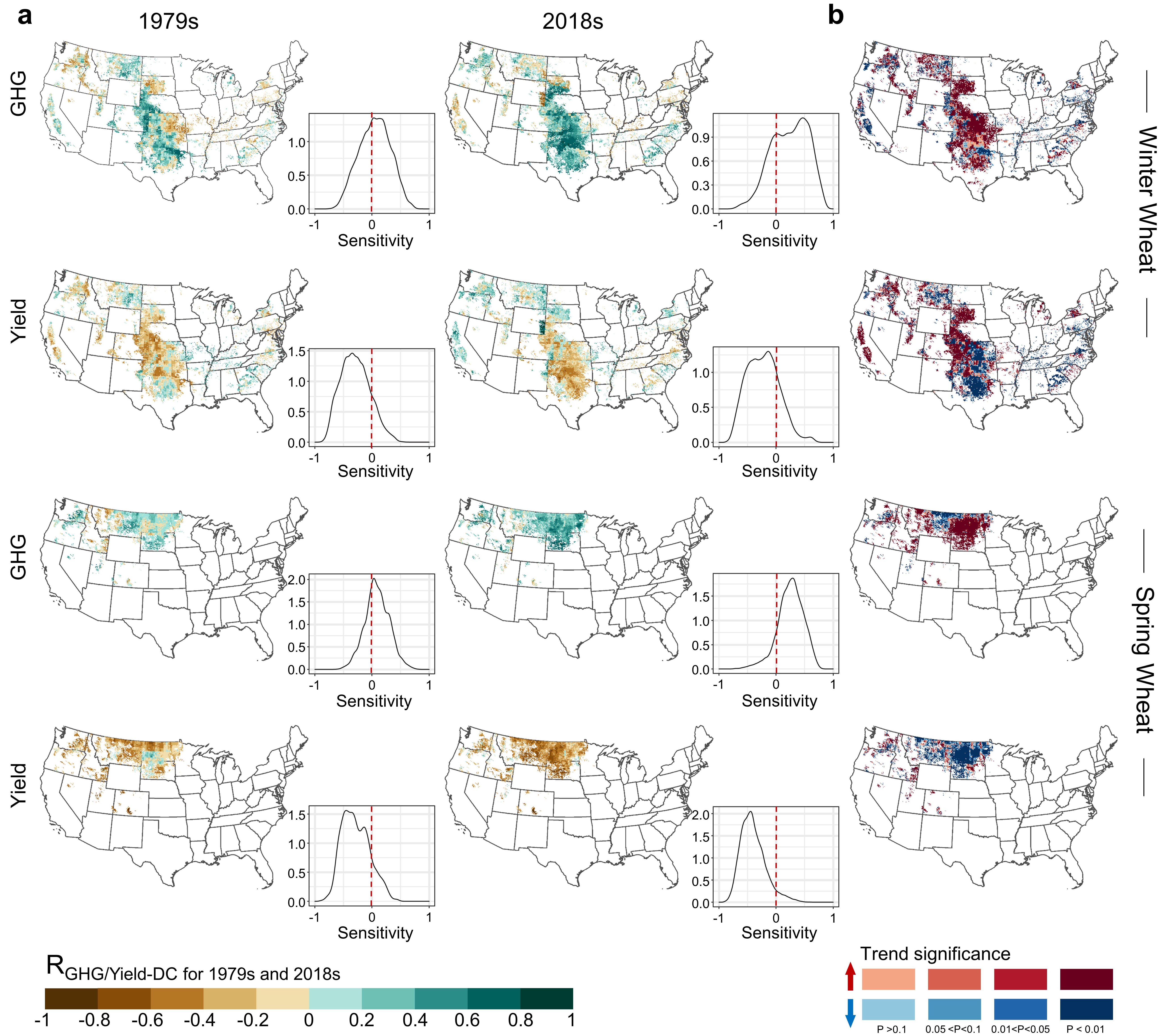
**

## Figure S16

**Sensitivity variation of net greenhouse gas (GHG) emissions and yield to dry conditions (DC) for wheat from the 1979s to 2018s.** A 20-year sliding window approach was used to examine the temporal variations in sensitivity to DC, with sensitivities within each window quantified using Pearson correlation coefficients (R), as detailed in Section 2.6 of the main text. Panel **a** shows the sensitivity maps for the first (1979s, 1960–1979) and last (2018s, 1999–2018) 20-year sliding windows. A probability density plot in the lower-left corner of each map illustrates the distribution of pixel-level sensitivities, with the horizontal axis representing R values and the vertical axis showing probability density. Panel **b** shows the linear temporal trend of sensitivity at the pixel scale, along with its statistical significance (P value).

**
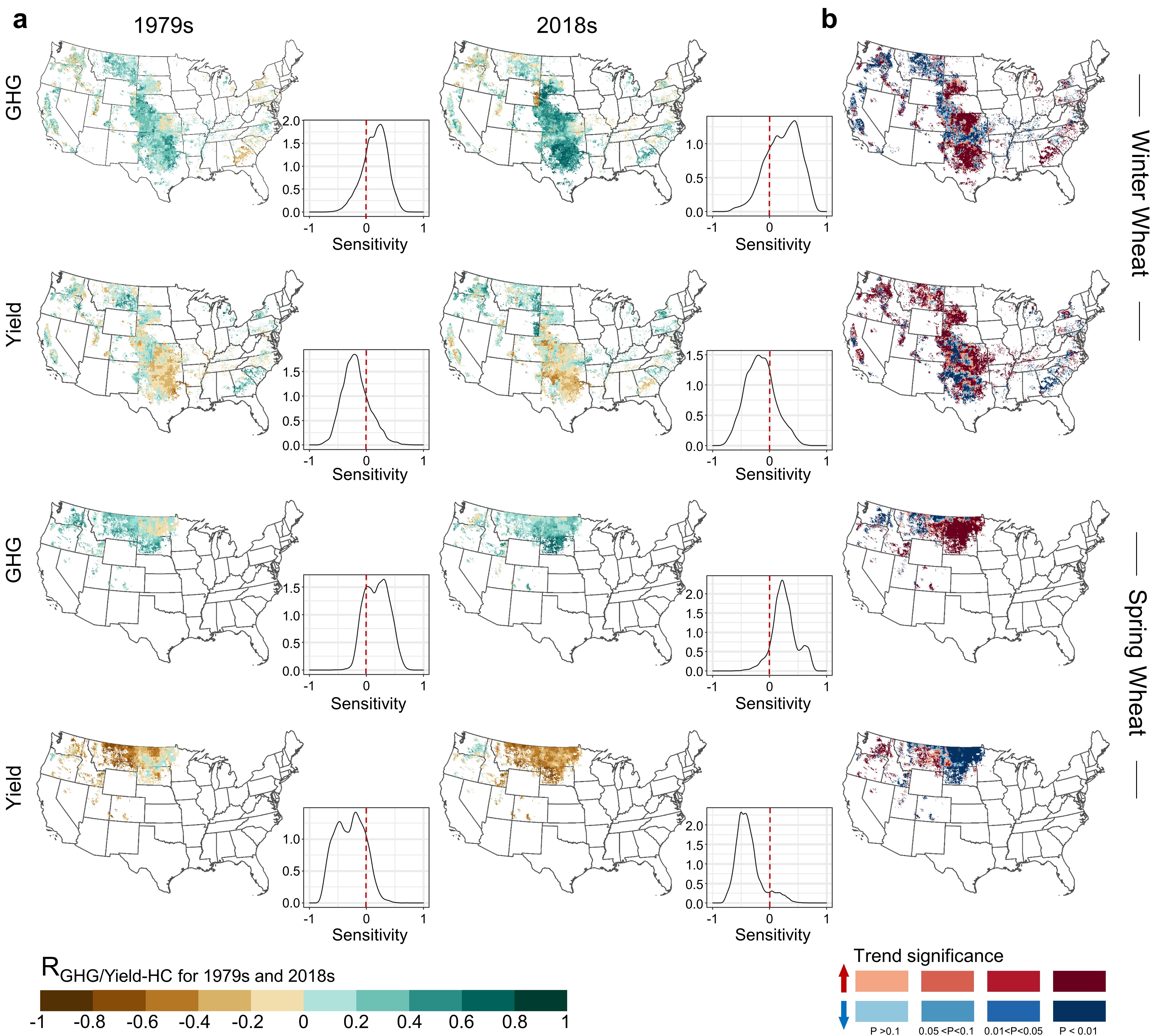
**

## Figure S17

**Sensitivity variation of net greenhouse gas (GHG) emissions and yield to heat conditions (HC) for wheat from the 1979s to 2018s.** A 20-year sliding window approach was used to examine the temporal variations in sensitivity to HC, with sensitivities within each window quantified using Pearson correlation coefficients (R), as detailed in Section 2.6 of the main text. Panel **a** shows the sensitivity maps for the first (1979s, 1960–1979) and last (2018s, 1999–2018) 20-year sliding windows. A probability density plot in the lower-left corner of each map illustrates the distribution of pixel-level sensitivities, with the horizontal axis representing R values and the vertical axis showing probability density. Panel **b** shows the linear temporal trend of sensitivity at the pixel scale, along with its statistical significance (P value).


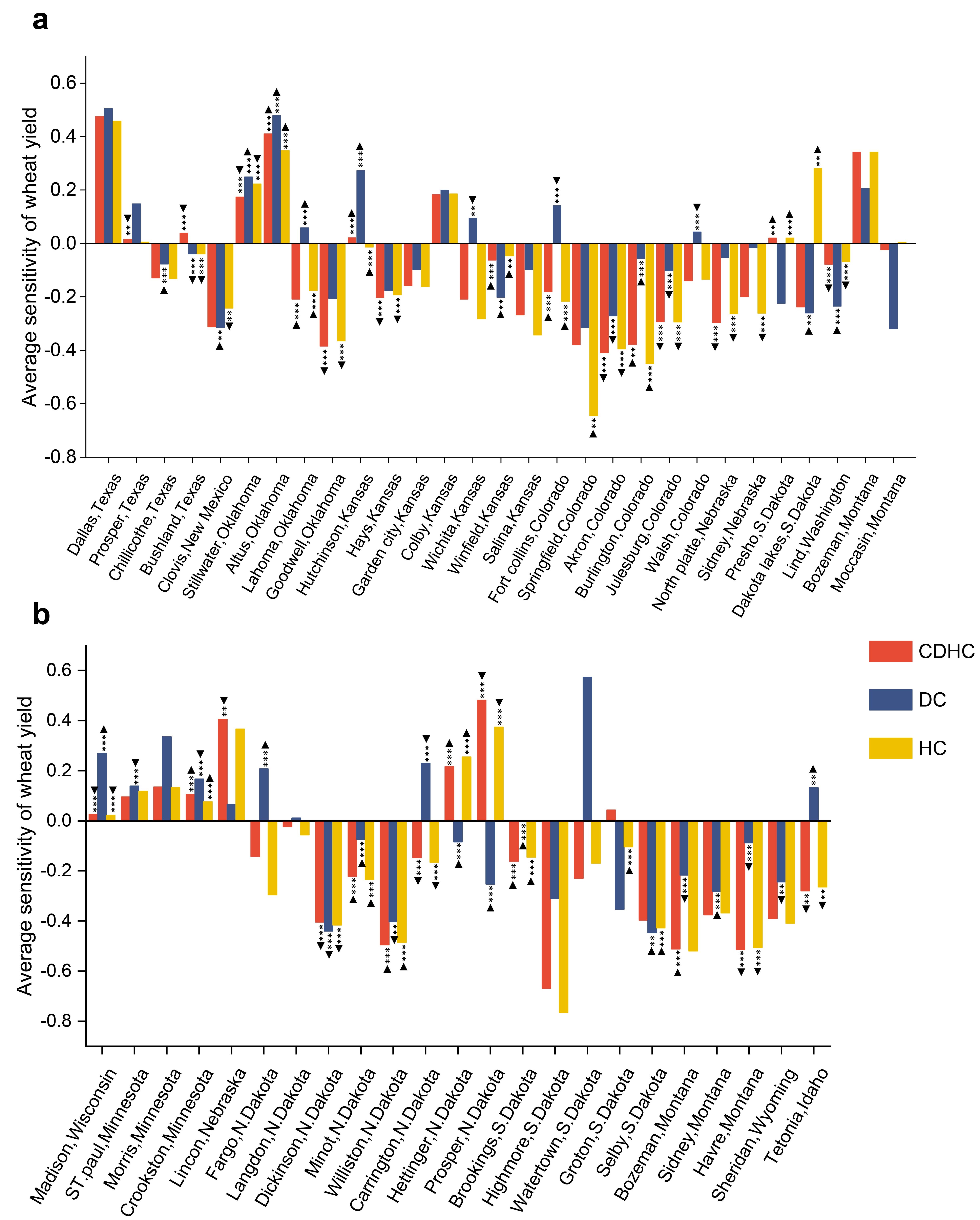


## Figure S18

**Sensitivity of wheat yield to extreme dry-heat conditions from nursery statistics over 1960-2018.** Panel **a** (winter wheat) and **b** (spring wheat) are the sensitivity of yield to dry-heat conditions. The direction of black triangle represents the trend (up or down) resulting from dry-heat conditions for wheat yield. Symbols for the significance test are: * for p < 0.05, and ** for p < 0.01. DC, HC, and CDHC represent heat, dry, and compound dry-heat conditions, respectively.


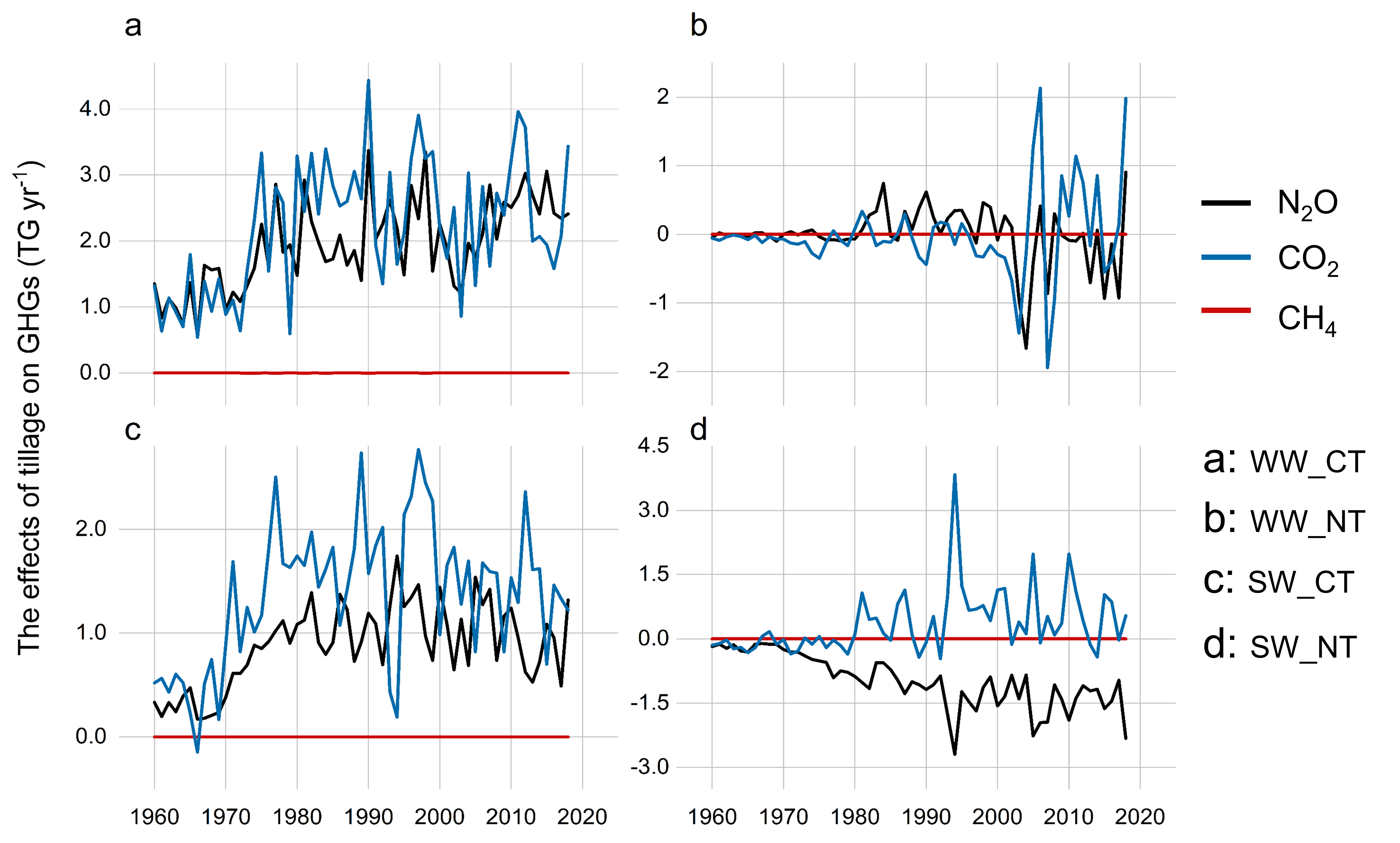


## Figure S19

**The impact of tillage practice switching on three greenhouse gases at the temporal scale**. CT and NT represent the switching from historical tillage practices to full CT and NT across the U.S., respectively. WW: winter wheat; SW: spring wheat; CT: convention tillage; NT: no tillage.


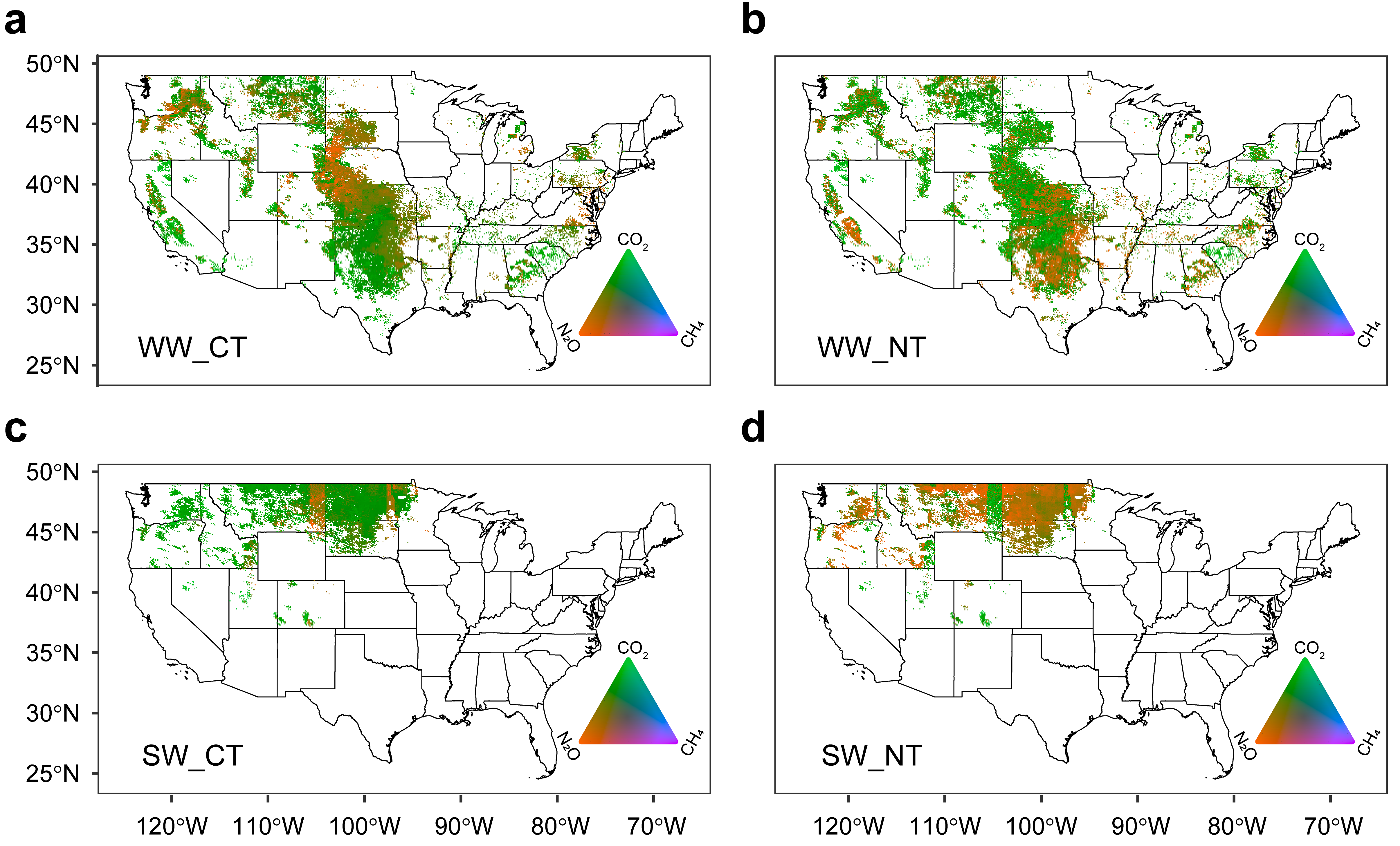


## Figure S20

**The impact of tillage practice switching on three greenhouse gases at the spatial scale**. CT and NT represent the switching from historical tillage practices to full CT and NT across the U.S., respectively. The spatial effects indicate the contribution of tillage switching to net greenhouse gas emissions, rather than providing specific values. WW: winter wheat; SW: spring wheat; CT: convention tillage; NT: no tillage.

**
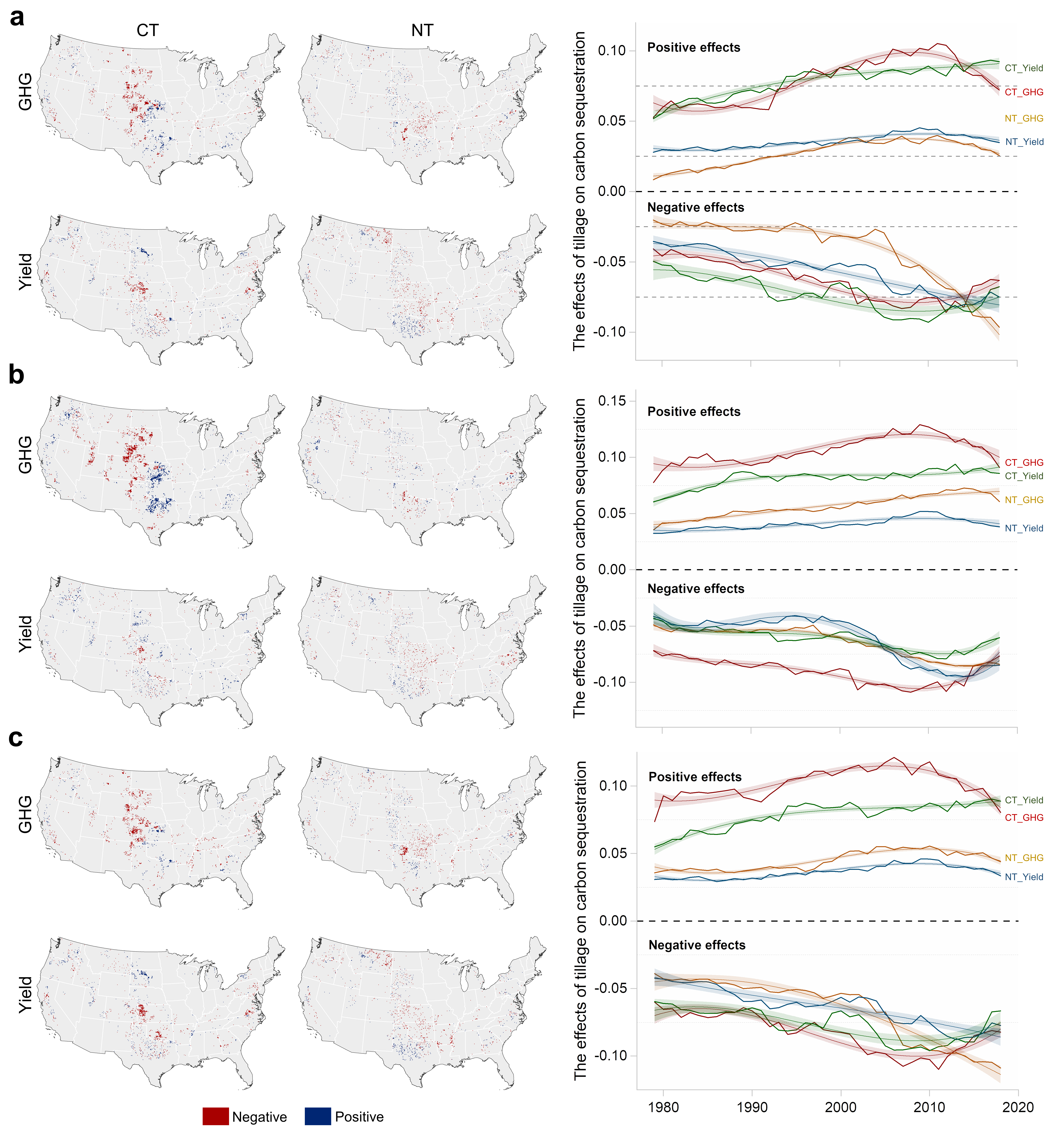
**

## Figure S21

**Effects of tillage management on the sensitivity of winter wheat net greenhouse gases (GHG) emission and yield under extreme dry-heat conditions**. Panel **a**, **b**, and **c** represent compound dry-heat, dry, and heat conditions, respectively. The red and blue pixels show consistently negative and positive effects resulting from tillage management under the dry-heat events to net GHG emissions and yield during 1979s to 2018s. The positive effects for net GHG emission and yield represent the uptake and increases, respectively, compared to the all-combined scenario and vice versa. The shaded bands indicate 95% confidence intervals for the mean predictions. CT: conventional tillage; NT: no tillage.

**
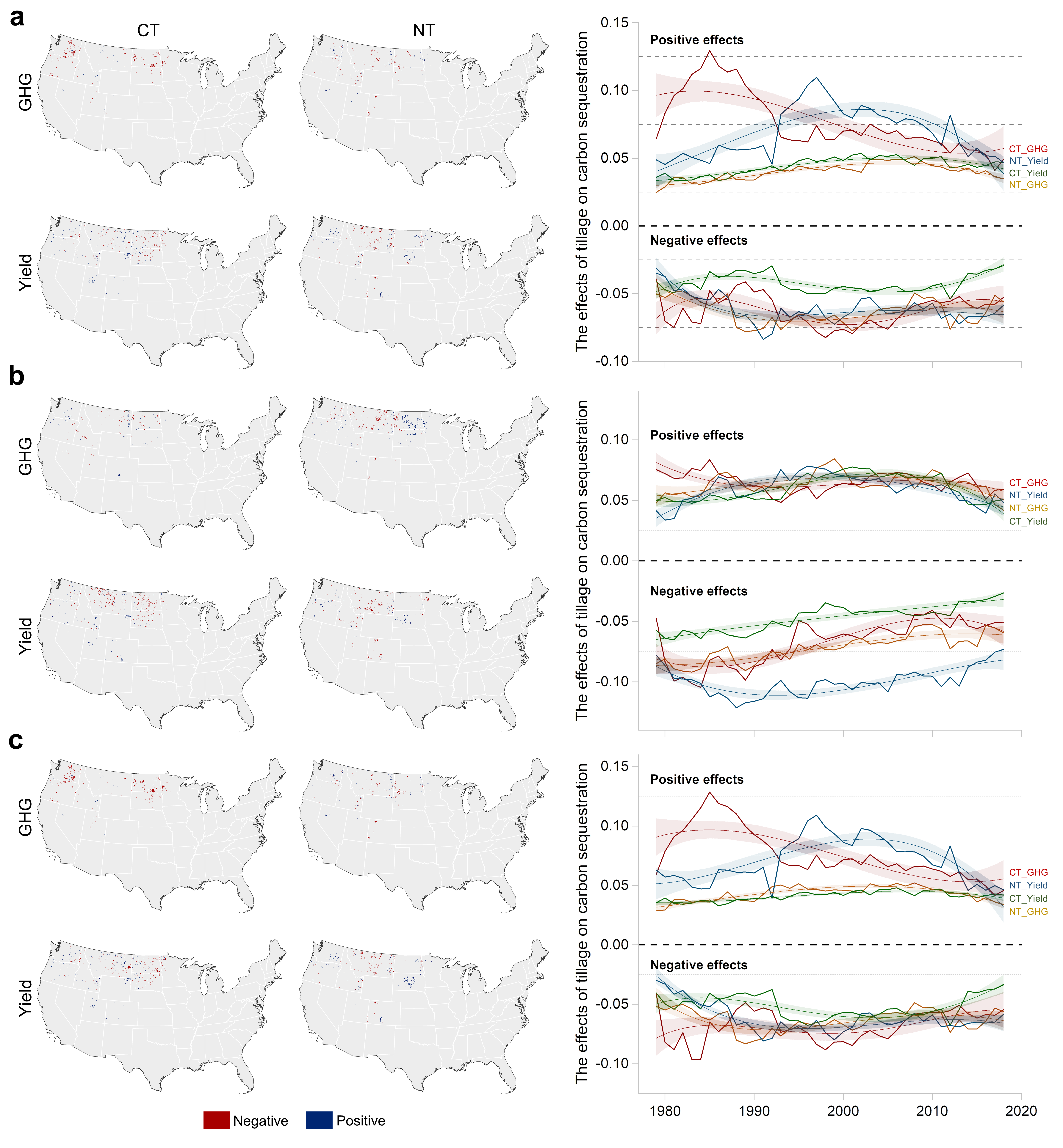
**

## Figure S22

**Effects of tillage management on the sensitivity of spring wheat net greenhouse gases (GHG) emission and yield under extreme dry-heat events**. Panel **a**, **b**, and **c** represent compound dry-heat, dry, and heat conditions, respectively. The red and blue pixels show consistently negative and positive effects resulting from tillage management under the dry-heat events to net GHG emissions and yield during 1979s to 2018s. The positive effects for net GHG emission and yield represent the uptake and increases, respectively, compared to the all-combined scenario and vice versa. The shaded bands indicate 95% confidence intervals for the mean predictions. CT: conventional tillage; NT: no tillage.


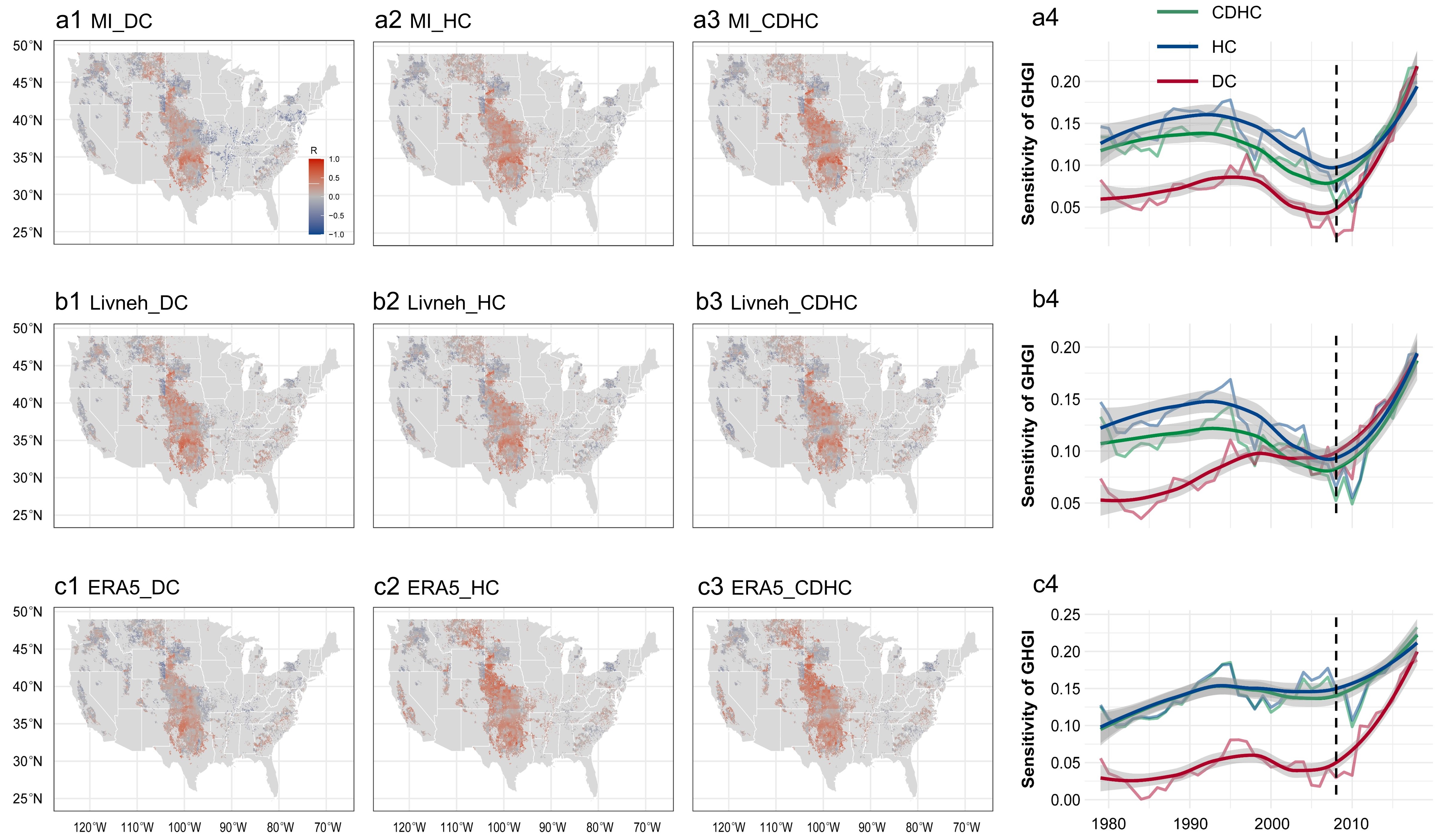


## Figure S23

**Spatiotemporal patterns of winter wheat sensitivity to dry-heat conditions based on different meteorological datasets**. The three datasets are sourced from Livneh, ERA5, and DLEM model inputs (MI). Sensitivity is expressed as the Pearson correlation coefficient between dry-heat conditions and GHGI. DC, HC, and CDHC represent heat, dry, and compound dry-heat conditions, respectively. The shaded bands indicate 95% confidence intervals for the mean predictions


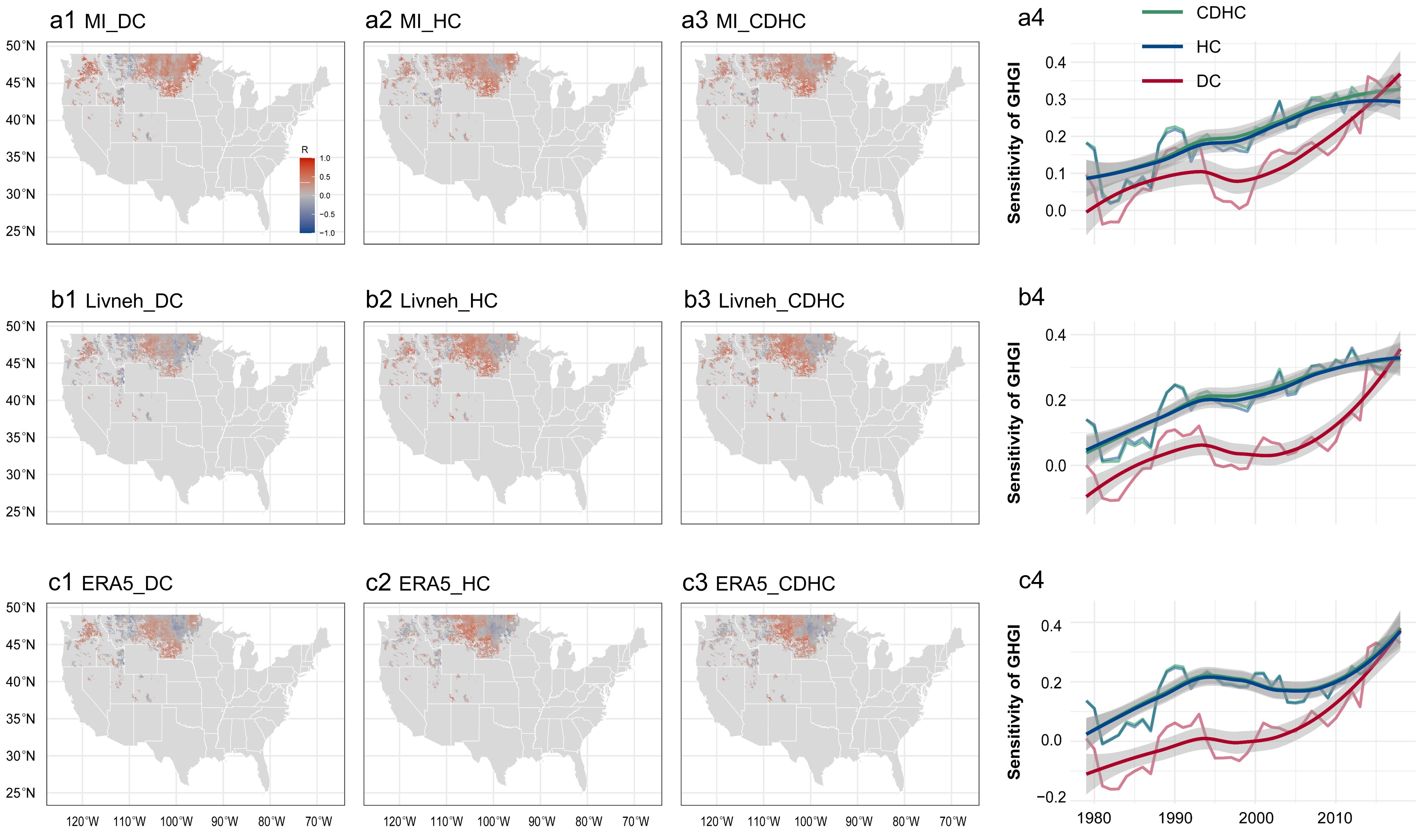


## Figure S24

**Spatiotemporal patterns of spring wheat sensitivity to dry-heat conditions based on different meteorological datasets.** The three datasets are sourced from Livneh, ERA5, and DLEM model inputs (MI). Sensitivity is expressed as the Pearson correlation coefficient between dry-heat conditions and GHGI. DC, HC, and CDHC represent heat, dry, and compound dry-heat conditions, respectively. The shaded bands indicate 95% confidence intervals for the mean predictions


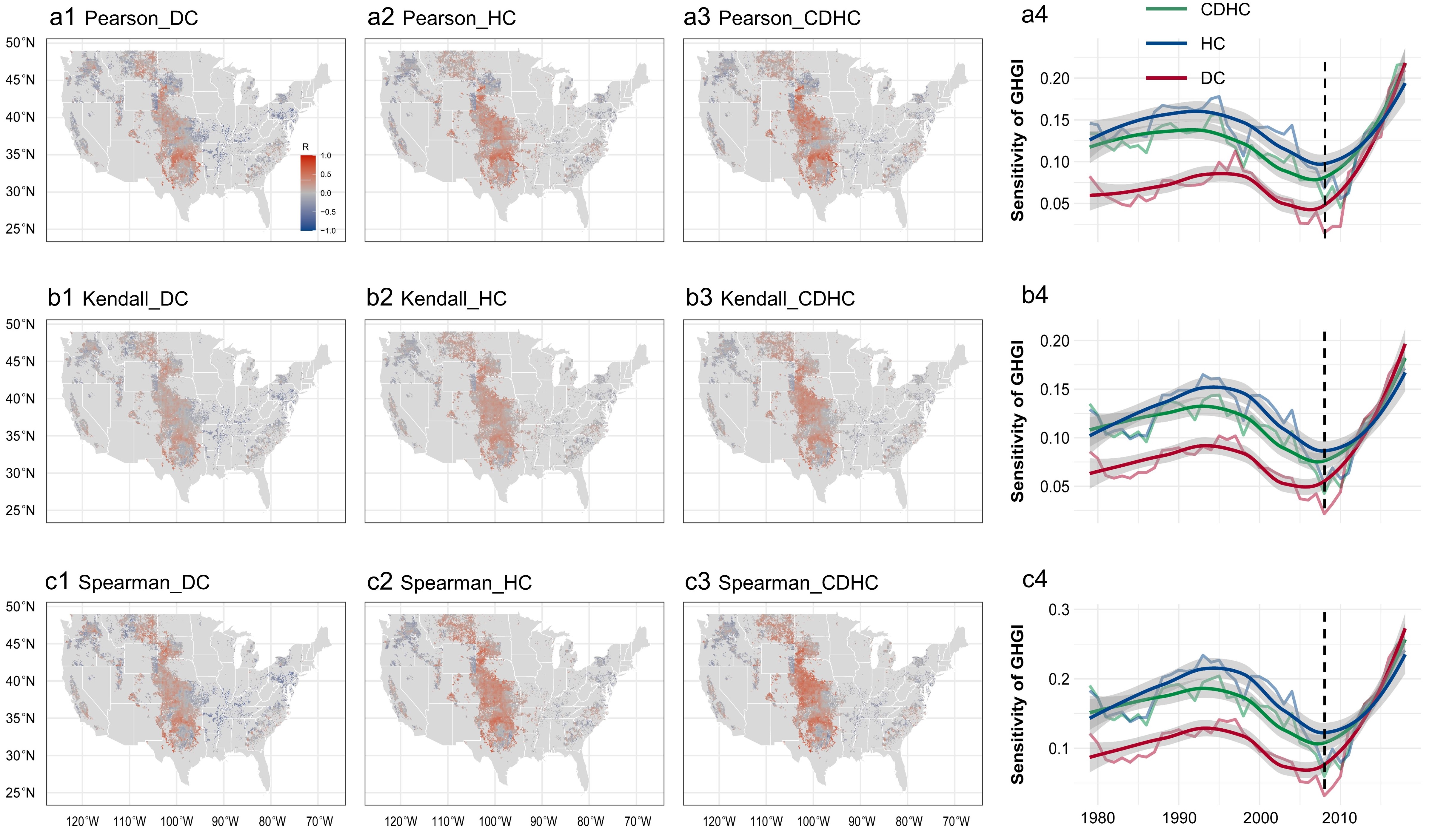


## Figure S25

**Spatiotemporal patterns of winter wheat sensitivity to dry-heat conditions based on different sensitivity methods: Pearson, Kendall, and Spearman**. Sensitivity was calculated using meteorological drivers from the DLEM. DC, HC, and CDHC represent heat, dry, and compound dry-heat conditions, respectively. The shaded bands indicate 95% confidence intervals for the mean predictions


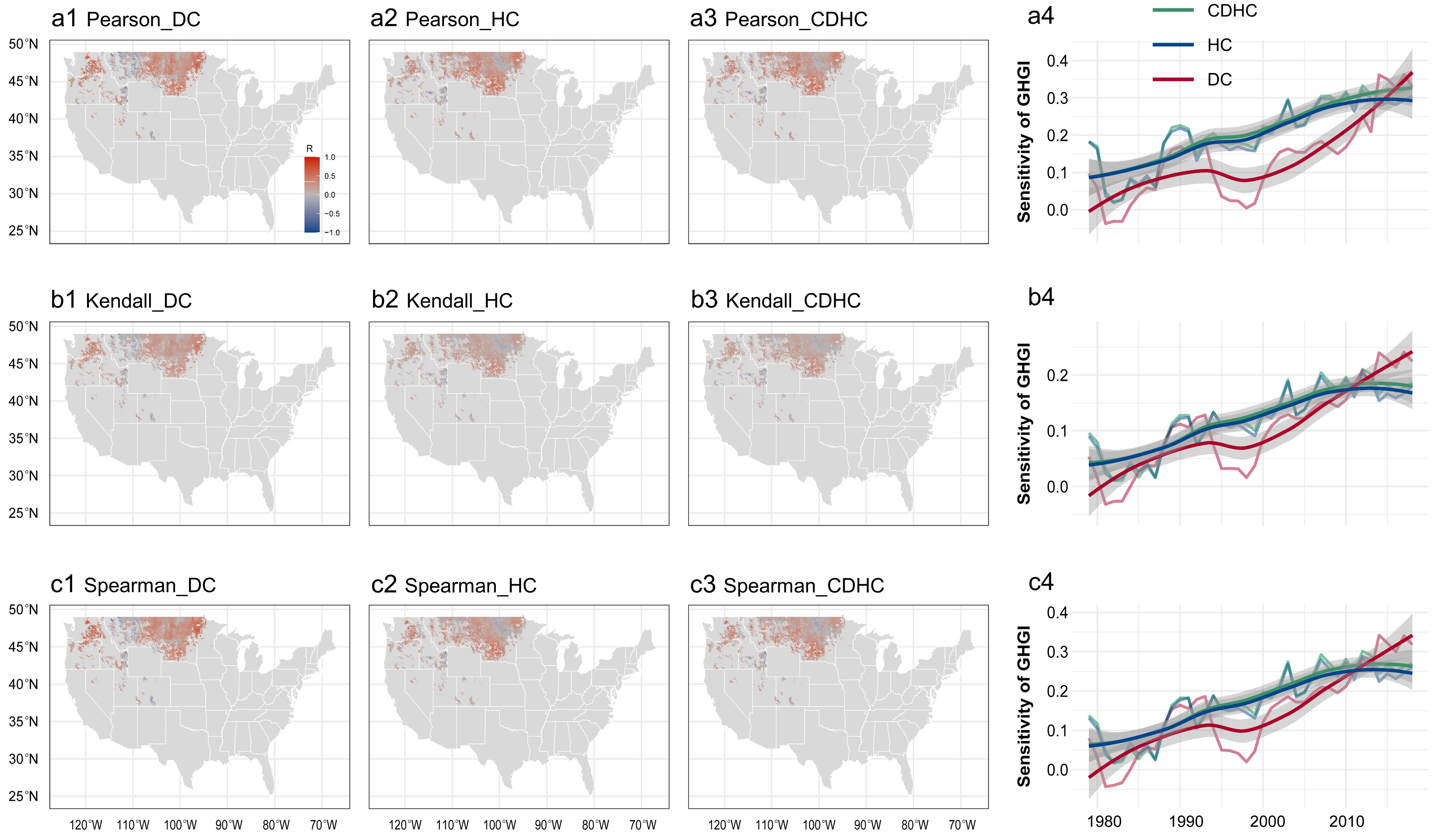


## Figure S26

**Spatiotemporal patterns of spring wheat sensitivity to dry-heat conditions based on different sensitivity methods: Pearson,** **Kendall, and Spearman**. Sensitivity was calculated using meteorological drivers from the DLEM. DC, HC, and CDHC represent heat, dry, and compound dry-heat conditions, respectively. The shaded bands indicate 95% confidence intervals for the mean predictions


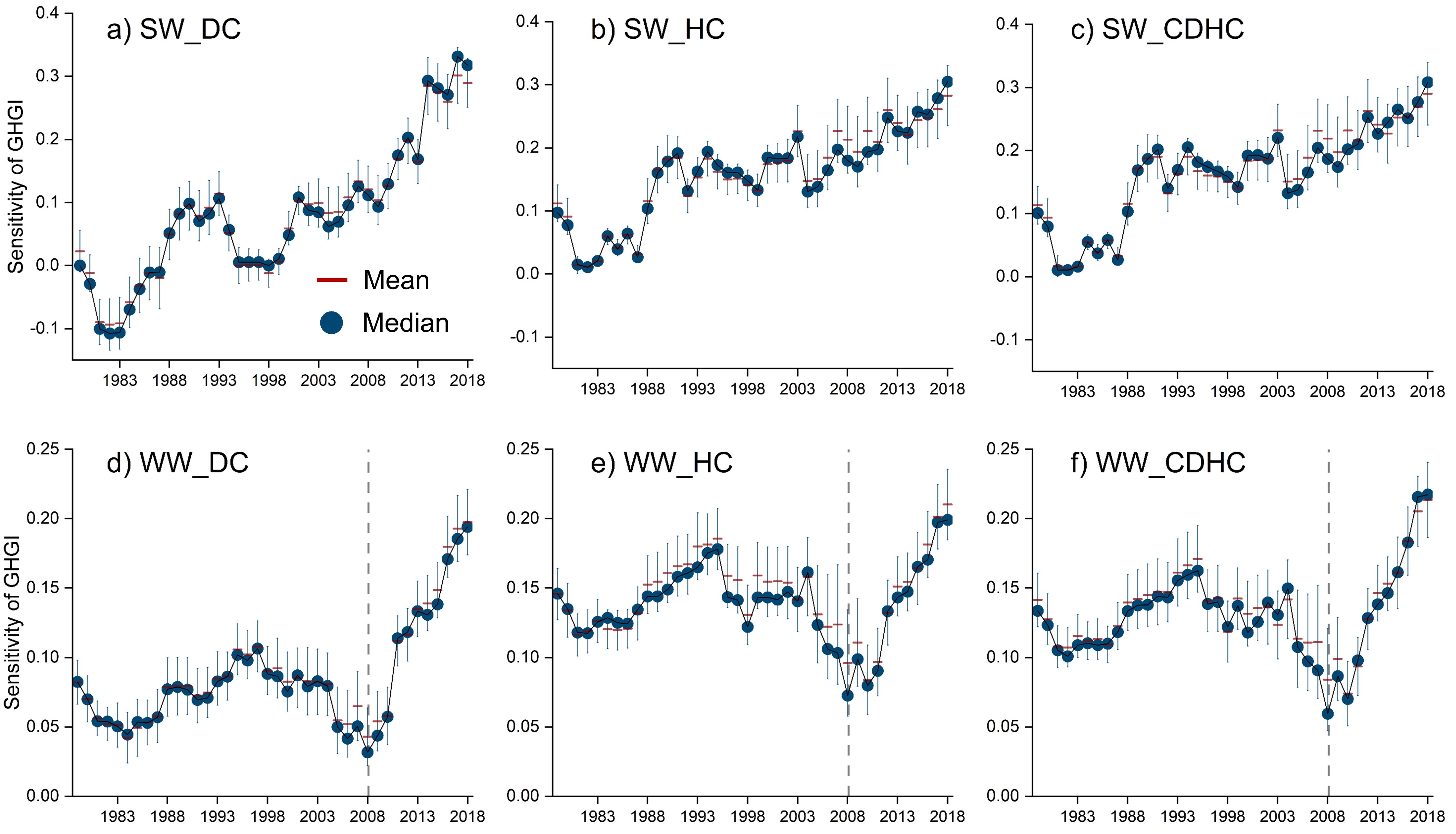


## Figure S27

**Uncertainty in GHGI sensitivity to dry-heat conditions based on an ensemble simulation using three meteorological datasets and three sensitivity methods.** The box plot whiskers represent Mean ± 95% confidence intervals. DC, HC, and CDHC represent heat, dry, and compound dry-heat conditions, respectively. WW: Winter wheat; SW: Spring wheat.


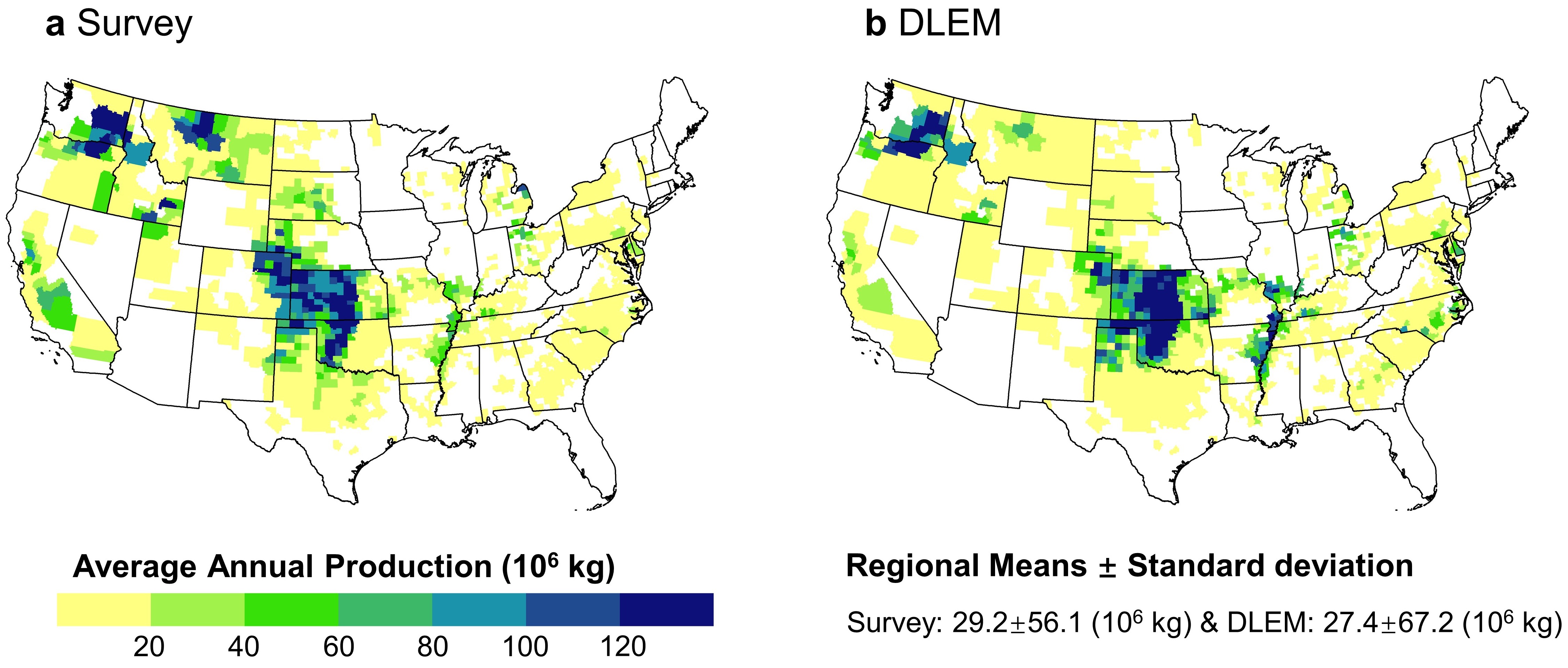


## Figure S28

**Regional comparisons between** **survey-based (a) and DLEM-based (b) estimations for winter wheat production.** The simulations from DLEM were averaged county level to match the format of survey data from United States Department of Agriculture (USDA, <https://quickstats.nass.usda.gov/>).


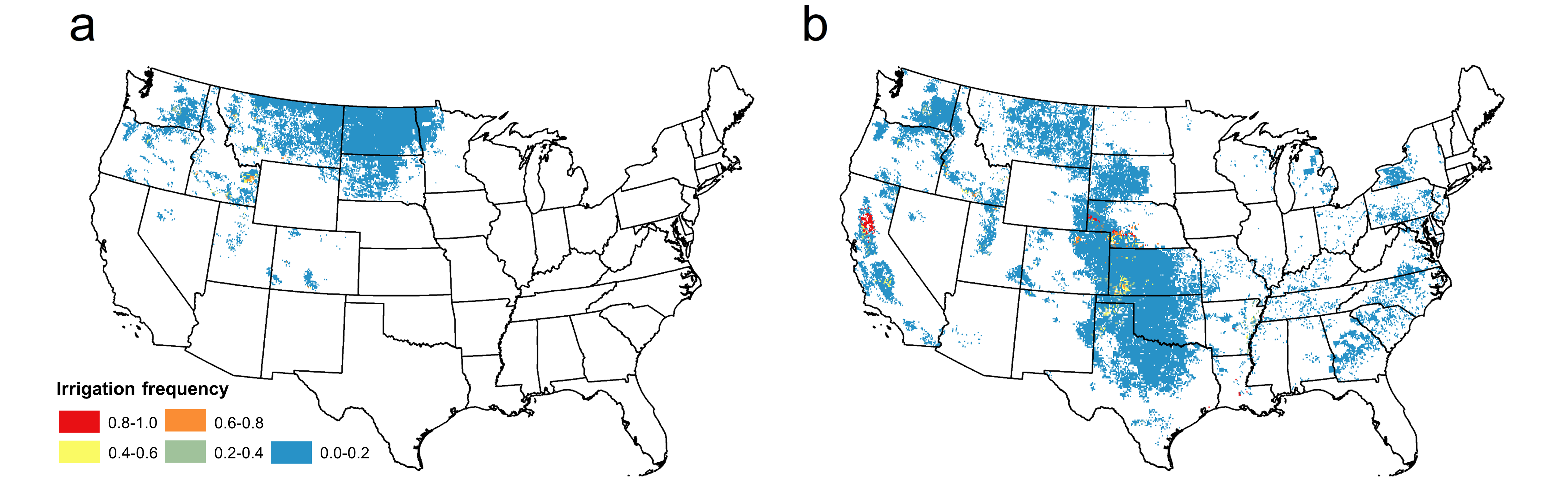


## Figure S29

**Irrigation frequency in the planting regions of US wheat during 1960 to 2018.** Panel a and b represent spring and winter wheat, respectively.

Table S1

Four tillage practices implemented in the DLEM 4.0.

| **Tillage parameters** | **No**  **tillage** | **Conservation tillage** | **Reduced**  **tillage** | **Conventional tillage** |
| --- | --- | --- | --- | --- |
| Depth (cm) | 5 | 10 | 20 | 20 |
| Mixing efficiency (%) | 5 | 50 | 90 | 90 |
| Soil layer inversion | No | Yes | Yes | Yes |
| Soil surface covered by residues after planting (%) | > 30 | 15 > & <30 | 15 > & <30 | < 15 |

**Table S2**

Details for drivers

| Dataset name | Period | Spatial  resolution | Temporal  resolution | Methods and data sources |
| --- | --- | --- | --- | --- |
| Climate (precipitation, solar  radiation, maximum, minimum  and mean temperatures) | 1860–2018 | 5 arc-min | Daily | Reconstructed from the North American Land Data Assimilation System product(Mitchell et al., 2004; Xia et al., 2012), the Climate Research Unit-National Centers for Environmental Prediction dataset (Mitchell and Jones, 2005), and the IPSL Climate Model dataset(Boucher et al., 2020), using a revised delta downscaling method(Liu et al., 2013) |
| CO_2_ concentration | 1860–2018 | 5 arc-min | Monthly | Obtained from the NOAA GLOBALVIEW-CO2 data set ([www.esrl.noaa.gov](http://www.esrl.noaa.gov/)) |
| Nitrogen deposition | 1860–2018 | 5 arc-min | Yearly | Acquired from the International Global Atmospheric Chemistry (IGAC)/Stratospheric Processes and Their Role in Climate (SPARC) Chemistry–Climate Model Initiative (CCMI)(Eyring et al., 2013) |
| Soil physical and chemical  properties (e.g., texture and pH) | One time | 5 arc-min | One time | Obtained from the ISRIC-WISE Harmonized Global Soil Profile dataset(Batjes, 2008) |
| Land use and land cover change (e.  g., cropland fraction) | 1860-2016 | 5 arc-min | Yearly | Acquired from Yu and Lu (2018)(Yu and Lu, 2018) |
| Crop rotation maps | 1910–2018 | 5 arc-min | Yearly | Developed by combining the United States Department of Agriculture (USDA) Cropland Data Layer (CDL) product, the USDA-National Agricultural Statistics Service (NASS) survey data of county-scale crop planting area, and the Google Earth Engine, using the spatialization method implemented in Yu et al. (2018)(Yu and Lu, 2018) |
| Crop-specific nitrogen fertilizer  use rate | 1910–2018 | State-level | Yearly | Reconstructed using the state-level N fertilizer use rates from USDA-NASS and the national-level commercial N fertilizer consumption data from Mehring et al. (1957)(Mehring et al., 1957) and USDA-ERS (https://www.ers.usda.gov/data-products/fertilizer-use-and-price/), following a method similar to that used in Cao et al. (2018)(Cao et al., 2018) |
| Manure nitrogen application | 1860–2018 | 5 arc-min | Yearly | Acquired from Bian et al. (2021)(Bian et al., 2021) |
| Crop-specific irrigation map | 1950–2018 | 5 arc-min | Yearly | Using the MODIS Irrigated Agriculture Dataset (MIrAD)(Pervez and Brown, 2010; Brown and Pervez, 2014) as a base map, and then combining the county-scale irrigation reanalysis dataset derived from the United States Geological Survey (USGS)(McManamay et al., 2021) and the USDA-NASS county-scale irrigated cropland area to extrapolate the spatially explicit irrigation map in historical years |
| Tillage map | 1960–2018 | 5 arc-min | Yearly | Reconstructed from the county-scale tillage practices survey data obtained from the National Crop Residue Management Survey (CRM) of the Conservation Technology Information Center (https://www.ctic.org/CRM), where tillage maps for missing years were kept consistent with the nearest years for which data were available |
| The earliest and latest crop  planting dates | One time | State-level | One time | Obtained from the USDA-NASS survey report(NASS, 2010) |
| Auxiliary data (e.g., topography  and river network) | One time | 5 arc-min | One time | Obtained from previous DLEM studies(Tian et al., 2010; Tian et al., 2012; Xu et al., 2019; Tian et al., 2020) |

## Table S3

Overview of data compiled by field-observation collection. A Y (yes) or N (no) indicate if yield, soil organic carbon (SOC), nitrous oxide (N_2_O), or methane (CH_4_) fluxes were reported in the study. A “×” indicates that a comparison of this practice (e.g., no-tillage versus conventional tillage, no fertilization versus fertilization) was included in the study, and a ‘+’ indicates that this practice was included in the study but without a comparison. In the absence of irrigation activities, the system is considered rainfed.

| Reference | Location | Lat | Long | Cropping system | Variables | | | | Management practices | | |
| --- | --- | --- | --- | --- | --- | --- | --- | --- | --- | --- | --- |
|  |  |  |  |  | Yield | SOC | N_2_O | CH_4_ | Nitrogen fertilization | Tillage | Irrigation |
| Kessavalou et al. (1998) | Sidney, NE | 41.23 | -103.00 | WW | Y | N | N | Y | + | × |  |
| Lamb et al. (1985) | Sidney, NE | 41.23 | -103.00 | WW | Y | Y | N | N | + | × |  |
| Halvorson et al. (1997) | Akron, CO | 40.15 | -103.15 | WW | Y | N | N | N |  | × |  |
| Cox (1991) | Fargo, ND | 46.88 | -97.23 | WW | Y | N | N | N |  | × |  |
| Cox (1991) | Langdon, ND | 48.75 | -98.33 | WW | Y | N | N | N |  | × |  |
| Cox (1991) | Minot, ND | 48.18 | -101.3 | WW | Y | N | N | N |  | × |  |
| Cox (1991) | Williston, ND | 48.13 | -103.75 | WW | Y | N | N | N |  | × |  |
| Machado et al. (2007) | Pendleton, OR | 45.78 | -118.68 | WW | Y | N | N | N | + | × |  |
| Lin and Chen (2014) | Bozeman, MT | 47.05 | -109.95 | WW | Y | N | N | N | + | × |  |
| Raun et al. (1998) | Oklahoma | 36.12 | -97.09 | WW | N | Y | N | N | × | + |  |
| Raun et al. (1998) | Oklahoma | 36.39 | -98.12 | WW | N | Y | N | N | × | + |  |
| Raun et al. (1998) | Oklahoma | 34.61 | -99.33 | WW | N | Y | N | N | × | + |  |
| O’Dea et al. (2015) | Bozeman, MT | 45.67 | -111.15 | WW | N | Y | N | N | + | + |  |
| Somenahally et al. (2018) | El Reno, OK | 35.57 | -98.04 | WW | N | Y | N | N | × | × |  |
| Sherrod et al. (2018) | Sterling, CO | 40.37 | -103.13 | WW | N | Y | N | N |  | + |  |
| Sherrod et al. (2018) | Stratton, CO | 39.18 | -102.26 | WW | N | Y | N | N |  | + |  |
| Lin et al. (2023) | Ashland, KS | 39.1167 | -96.6 | WW | N | Y | N | N | × | × |  |
| Machado et al. (2006) | Pendleton, OR | 45.7 | -118.6 | WW | N | Y | N | N | × | × |  |
| Cambardella and Elliott (1992) | Sidney, NE | 41.23 | -103.00 | WW | N | Y | N | N |  | × |  |
| Doran et al. (1998) | Sidney, NE | 41.23 | -103.00 | WW | N | Y | N | N | + | × |  |
| Mikha et al. (2013) | Akron, CO | 40.15 | -103.15 | WW | N | Y | N | N | + | × |  |
| Blanco-Canqui et al. (2011) | Hutchinson, KS | 38.3 | -97.55 | WW | N | Y | N | N |  | × |  |
| Potter et al. (1998) | Bushland, TX | 35.19 | -102.08 | WW | N | Y | N | N | × | × |  |
| Dusenbury et al. (2008) | Bozeman, MT | 45.67 | -111.15 | WW-SW | N | N | Y | N | × | + |  |
| Sainju et al. (2020) | Sidney, MT | 48.55 | -104.83 | SW | N | N | Y | N | + | + |  |
| Bista et al. (2017) | Laramie, WY | 42.12 | -104.39 | WW | N | N | Y | Y |  | + |  |
| Gelfand et al. (2016) | KBS, MI | 42.4 | -85.4 | WW | N | N | Y | N | + | + |  |
| Waldo et al. (2019) | Pullman, WA | 46.78 | -117.09 | WW | N | N | Y | N | × | × |  |
| Sainju et al. (2021) | Bozeman, MT | 45.67 | -111.15 | SW | N | N | N | Y | × | + |  |
| Hurisso et al. (2016) | Sidney, NE | 41.25 | -103 | WW | N | N | N | Y | × | × |  |
| Liebig et al. (2010) | Mandan, ND | 46.77 | -100.91 | WW | N | N | N | Y | + | + |  |
| Liebig et al. (2019) | Mandan, ND | 46.771 | -100.949 | SW | N | N | N | Y | + | + |  |

## Table S4

Experiments design

| **No.** | **Scenario** | **Climate** | **CO_2_** | **Ndep** | **Tillage** | **Nfer** | **Manure** | **Irrigation** | **LULC** |
| --- | --- | --- | --- | --- | --- | --- | --- | --- | --- |
| S1 | Baseline | 1860 | 1860 | 1860 | 1860 | 1860 | 1860 | 1860 | 1960 |
| S2 | With all drivers | 1860-2018 | 1860-2018 | 1860-2018 | 1860-2018 | 1860-2018 | 1860-2018 | 1860-2018 | 1960-2018 |
| S3 | Without climate | 1860 | 1860-2018 | 1860-2018 | 1860-2018 | 1860-2018 | 1860-2018 | 1860-2018 | 1960-2018 |
| S4 | Without CO_2_ | 1860-2018 | 1860 | 1860-2018 | 1860-2018 | 1860-2018 | 1860-2018 | 1860-2018 | 1960-2018 |
| S5 | Without N deposition | 1860-2018 | 1860-2018 | 1860 | 1860-2018 | 1860-2018 | 1860-2018 | 1860-2018 | 1960-2018 |
| S6 | Without tillage | 1860-2018 | 1860-2018 | 1860-2018 | 1860 | 1860-2018 | 1860-2018 | 1860-2018 | 1960-2018 |
| S7 | Without N fertilization | 1860-2018 | 1860-2018 | 1860-2018 | 1860-2018 | 1860 | 1860-2018 | 1860-2018 | 1960-2018 |
| S8 | Without manure | 1860-2018 | 1860-2018 | 1860-2018 | 1860-2018 | 1860-2018 | 1860 | 1860-2018 | 1960-2018 |
| S9 | Without irrigation | 1860-2018 | 1860-2018 | 1860-2018 | 1860-2018 | 1860-2018 | 1860-2018 | 1860 | 1960-2018 |
| S10 | Climate+CO_2_+Ndep | 1860-2018 | 1860-2018 | 1860-2018 | 1860 | 1860 | 1860 | 1860 | 1960 |
| S11 | Climate+CO_2_+Ndep+LULC | 1860-2018 | 1860-2018 | 1860-2018 | 1860 | 1860 | 1860 | 1860 | 1960-2018 |
| S12 | No-tillage (NT) | 1860-2018 | 1860-2018 | 1860-2018 | ALL NT | 1860-2018 | 1860-2018 | 1860-2018 | 1960-2018 |
| S13 | Conventional tillage (CT) | 1860-2018 | 1860-2018 | 1860-2018 | ALL CT | 1860-2018 | 1860-2018 | 1860-2018 | 1960-2018 |

Note: LULC, land use and cover change.

Daily mean, minimum and maximum temperature, precipitation, and shortwave solar radiation were included in climate-model simulations.

In the simulation experiments with LULC, the spatial distributions of wheat cropping before 1960 were assume to be constant at the level of 1960 due to limited data.

In addition, LULC was shown as spatial distribution because the value is calculated based on unit area, i.e.,

LULC statistics in every year = Value_1960_ *(changing area in every year for wheat) + value_1960_

(Similar to the linear changes related to the Value_1960_)

**Table S5**

Multifactor- and management-practice induced GHGI (kg CO^2^-eq/kg), production (×10^9^ kg/year), and soil GHG (Tg CO^2^-eq/year) from 1949 to 2012 for US wheat

|  |  | Winter wheat | | Spring wheat | |
| --- | --- | --- | --- | --- | --- |
| Output | Year | Mean | Trend | Mean | Trend |
| **Multifactor induced** |  |  |  |  |  |
| GHG emissions intensity (kg CO_2_-eq/kg) | 1960-2018 | 0.13 | -0.005** | 0.04 | 0.004** |
|  | 1960-1990 | 0.23 | 0.003 | -0.03 | 0.002 |
|  | 1990-2018 | 0.02 | -0.009** | 0.11 | 0.009* |
| Production (×10^9^ kg/year) | 1960-2018 | 40.17 | 0.238** | 15.39 | 0.217** |
|  | 1960-1990 | 37.44 | 1.035** | 11.88 | 0.364** |
|  | 1990-2018 | 43.18 | -0.544** | 19.29 | -0.219** |
| GHG emissions (Tg CO_2_-eq/year) | 1960-2018 | 4.91 | -0.168** | 0.67 | 0.068** |
|  | 1960-1990 | 8.53 | 0.355** | -0.31 | 0.014 |
|  | 1990-2018 | 0.91 | -0.410** | 1.75 | 0.147 |
| **Nitrogen fertilizer induced** |  |  |  |  |  |
| GHG emissions intensity (kg CO_2_-eq/kg) | 1960-2018 | 0.01 | -0.001 | -0.085 | 0.005** |
|  | 1960-1990 | 0.04 | 0.007** | -0.159 | 0.004 |
|  | 1990-2018 | -0.03 | -0.005 | 0.003 | 0.009** |
| Production (×10^9^ kg/year) | 1960-2018 | 16.21 | 0.263** | 5.23 | 0.131** |
|  | 1960-1990 | 12.49 | 0.528** | 3.00 | 0.157** |
|  | 1990-2018 | 20.33 | -0.119 | 7.71 | -0.110 |
| GHG emissions (Tg CO_2_-eq/year) | 1960-2018 | 1.99 | -0.083 | -0.55 | 0.062** |
|  | 1960-1990 | 4.10 | 0.354** | -1.45 | 0.004 |
|  | 1990-2018 | -0.34 | -0.279* | 0.45 | 0.158** |
| **Manure induced** |  |  |  |  |  |
| GHG emissions intensity (kg CO_2_-eq/kg) | 1960-2018 | 0.003 | -0.000** | 0.003 | 0.000 |
|  | 1960-1990 | 0.004 | -0.000 | 0.002 | 0.001** |
|  | 1990-2018 | 0.001 | -0.000 | 0.003 | -0.000** |
| Production (×10^9^ kg/year) | 1960-2018 | 0.15 | 0.004** | 0.00 | -0.001** |
|  | 1960-1990 | 0.09 | 0.002 | 0.01 | -0.002** |
|  | 1990-2018 | 0.22 | 0.002 | 0.00 | 0.001 |
| GHG emissions (Tg CO_2_-eq/year) | 1960-2018 | 0.10 | -0.003* | 0.049 | 0.001 |
|  | 1960-1990 | 0.15 | 0.004 | 0.042 | 0.006** |
|  | 1990-2018 | 0.04 | -0.006* | 0.058 | -0.004** |
| **Irrigation induced** |  |  |  |  |  |
| GHG emissions intensity (kg CO_2_-eq/kg) | 1960-2018 | -0.018 | 0.000 | -0.005 | 0.000 |
|  | 1960-1990 | -0.022 | -0.002** | -0.006 | -0.000** |
|  | 1990-2018 | -0.013 | 0.001** | -0.004 | 0.000** |
| Production (×10^9^ kg/year) | 1960-2018 | 0.19 | 0.001 | 0.08 | 0.001 |
|  | 1960-1990 | 0.20 | 0.013** | 0.07 | 0.004** |
|  | 1990-2018 | 0.17 | -0.009* | 0.10 | -0.002** |
| GHG emissions (Tg CO_2_-eq/year) | 1960-2018 | -0.73 | 0.003 | -0.069 | 0.000 |
|  | 1960-1990 | -0.89 | -0.072** | -0.075 | -0.005** |
|  | 1990-2018 | -0.55 | 0.055** | -0.062 | 0.006** |
| **Tillage induced** |  |  |  |  |  |
| GHG emissions intensity (kg CO_2_-eq/kg) | 1960-2018 | 0.063 | 0.004** | -0.004 | 0.000** |
|  | 1960-1990 | 0.008 | 0.001** | -0.007 | 0.000** |
|  | 1990-2018 | 0.124 | 0.008** | -0.001 | 0.000 |
| Production (×10^9^ kg/year) | 1960-2018 | -0.18 | 0.073** | -0.13 | -0.005** |
|  | 1960-1990 | -1.24 | -0.051** | -0.05 | -0.002** |
|  | 1990-2018 | 1.00 | 0.225** | -0.21 | -0.007** |
| GHG emissions (Tg CO_2_-eq/year) | 1960-2018 | 2.44 | 0.160** | -0.076 | 0.000 |
|  | 1960-1990 | 0.09 | 0.024** | -0.087 | 0.000 |
|  | 1990-2018 | 5.06 | 0.318** | -0.064 | -0.003 |

Note: The stars indicate statistical significance from MK trend test at the 0.95 and 0.99 level (**p* < 0.05 and ***p*<0.01). GHG represent greenhouse gas emissions. GHGI is GHG intensity

**References**
