# Supplementary material for: Extreme Dry‐Heat Climate Impacts on Greenhouse Gas Emission Intensity in Wheat Production: Insights and Mitigation Strategies
Source: Glob Chang Biol. 2025 Jul 11;31(7):e70349. doi: 10.1111/gcb.70349 (PMC12247449; doi:10.1111/gcb.70349)
Supplement: Supplementary file 2 — Table S6. [file GCB-31-e70349-s002.docx]

**Table S6**

Explanation of Symbols in Equations and Variables

|  | **Symbol** | **Description** | **Unit** |
| --- | --- | --- | --- |
| 1 | *AI* | Aridity index | Unitless |
| 2 | *AKW* | Actual kernel weight at physiological maturity | g |
| 3 | $ATT$ | Accumulated thermal time | ℃ |
| 4 | $ATT_{min}$ | Threshold of minimum thermal time | ℃ |
| 5 | $avn$; $avn_{opt}$ | Actual and optimum available soil N | g N/m^2^ |
| 6 | $av_{NH4}$ | Available ammonia nitrogen in soil | g N/m^2^ |
| 7 | $BD_{soil}$ | Soil bulk density | g soil/m^3^ |
| 8 | $Card_{max}$;$Card_{min}$;  $Card_{opt1}$;$Card_{opt2}$ | Crop-specific maximum, minimum, lower optimal, and upper optimal air temperatures required for photosynthesis | ℃ |
| 9 | *CDHC* | Number of days with simultaneous dry and heat conditions for the period from March 1 through to September 30 in each year | days |
| 10 | $CPP$ | Cultivar-specific critical daylength | Hour |
| 11 | $CUMVER$ | Cumulative vernalization days | Days |
| 12 | $D_{t}$ | Tillage depth | cm |
| 13 | *DC* | Number of days when the AI falls below 0.2 for the period from March 1 through to September 30 | days |
| 14 | $DGHG_{t-a}$ | Sensitivity differences in GHG between the tillage and actual scenarios | Unitless |
| 15 | $DGHGI_{t-a}$ | Sensitivity differences in GHGI between the tillage and actual scenarios | Unitless |
| 16 | $DY_{t-a}$ | Sensitivity differences in yield between the tillage and actual scenarios | Unitless |
| 17 | $dailyBD$ | Daily crop development rate | ℃ |
| 18 | $dailyTT$ | Daily thermal time | ℃ |
| 19 | $E_{CO_{2}}$ | CO_2_ emissions in CO_2_ equivalents | Tg CO_2_-eq yr^-1^ or kg CO_2_-eq ha^-1^ |
| 20 | $E_{CH_{4}}$ | CH_4_ emissions in CO_2_ equivalents | Tg CO_2_-eq yr^-1^ or kg CO_2_-eq ha^-1^ |
| 21 | $E_{N_{2}O}$ | N_2_O emissions in CO_2_ equivalents | Tg CO_2_-eq yr^-1^ or kg CO_2_-eq ha^-1^ |
| 22 | $E_{GHG}$ | Net GHG emissions | Tg CO_2_-eq yr^-1^ or kg CO_2_-eq ha^-1^ |
| 23 | $EF_{mix}$ | Mixing efficiency of tillage | Unitless |
| 24 | $EVAP_{lit}$ | Litter evaporation | mm |
| 25 | $EVAP_{soil}$ | Soil evaporation | mm |
| 26 | $ext_{coef}$ | Canopy light extinction coefficient | Unitless |
| 27 | $F_{CO_{2}}$ | CO_2_ flux | Tg C yr^-1^ or  kg C ha^-1^ |
| 28 | $F_{CH_{4}}$ | CH_4_ flux | Tg C yr^-1^ or  kg C ha^-1^ |
| 29 | $F_{N_{2}O}$ | N_2_O flux | Tg N yr^-1^ or  kg N ha^-1^ |
| 30 | $f_{BDtill,pri}$ | Bulk density effect before tillage | Unitless |
| 31 | $f_{BDtill,aft}$ | Fraction of bulk density changes after tillage | Unitless |
| 32 | $f_{cm,i}$ | Cumulative effect of tillage at day *i* | Unitless |
| 33 | $f_{lit}$ | Fraction of soil surface covered by litter | Unitless |
| 34 | $f_{mix}$ | Vertical displacement of litter under no-tillage and natural vegetation conditions | Unitless |
| 35 | $f_{till}$ | Tillage scalar | Unitless |
| 36 | $f_{stress}$ | Environmental stress effect on crop development rate | Unitless |
| 37 | $fCBD$ | Fraction of accumulated $dailyBD$ | Unitless |
| 38 | $fheat$ | Heat stresses during crop development | Unitless |
| 39 | $fL$ | Light stresses during crop development | Unitless |
| 40 | $fN$ | Nitrogen stresses during crop development | Unitless |
| 41 | $froot$ | Root fraction in the soil layer | Unitless |
| 42 | $fW$ | Drought stresses during crop development | Unitless |
| 43 | $f\left(c_{av_{NO3}}\right)$ | Effect of nitrate concentration | Unitless |
| 44 | $f\left(clay_{_{soil\_dec}}\right)$ | Effect of soil texture on litter decomposition | Unitless |
| 45 | $f\left(clay_{N2O\_emi}\right)$ | Effect of soli texture on N_2_O emission | Unitless |
| 46 | $f\left(N_{_{soil\_dec}}\right)$ | Effect of N availability on litter decomposition | Unitless |
| 47 | $f\left(N_{mi}\right)$ | Effect of N availability when mineralization occurs | Unitless |
| 48 | $f\left(N_{im}\right)$ | Effect of N availability when immobilization occurs | Unitless |
| 49 | $f\left(T_{soil\_dec}\right)$ | Effect of soil temperature on litter decomposition | Unitless |
| 50 | $f\left(T_{soil\_N2O\_emi}\right)$ | Effect of soil temperature on N_2_O emission | Unitless |
| 51 | $f\left(T_{soil\_nit\_emi}\right)$ | Effect of soil temperature on N₂O emissions in nitrification process | Unitless |
| 52 | $f\left(T_{soil\_denit}\right)$ | Effect of soil temperature on denitrification process | Unitless |
| 53 | $f\left(T_{soil\_nit}\right)$ | Effect of soil temperature on nitrification | Unitless |
| 54 |  | Effect of soil moisture on litter decomposition | Unitless |
| 55 | $f\left(w_{soil\_N2O\_emi}\right)$ | Effect of soil moisture on N_2_O emission | Unitless |
| 56 | $f\left(w_{soil\_denit}\right)$ | Effect of soil moisture on denitrification process | Unitless |
| 57 | $f\left(w_{soil\_nit}\right)$ | Effect of soil moisture on nitrification | Unitless |
| 58 | *HC* | Number of days when the daily maximum air temperature exceeds 30°C for the period from March 1 through to September 30 | days |
| 59 | $k_{nit}$ | Potential fraction of NH_4_^+^ | Unitless |
| 60 | $k_{np}$ | Proportion of N intermediates resulting in N_2_O in nitrification process | Unitless |
| 61 | $k_{pool}$ | Actual decomposition rate of each litter pool | g C/m^2^/yr |
| 62 | $k_{pot,pool}$ | Potential decomposition rate of each litter pool | g C/m^2^/yr |
| 63 | $kden$ | Plant functional type-dependent parameters for denitrification | g N/m^2^/d |
| 64 | $Litter_{ag}$ | Aboveground litter pool | g C/m^2^ |
| 65 | $Litter_{bg}$ | Belowground litter pool | g C/m^2^ |
| 66 | *M* | Total number of soil layers affected by tillage operation | Unitless count |
| 67 | $N_{denit}$ | Denitrification | g N/m^2^/d |
| 68 | $N_{nit}$ | Nitrification | g N/m^2^/d |
| 69 | $N_{pot,denit}$ | Potential denitrification rate | g N/m^2^/d |
| 70 | *N_2_O* | Nitrous oxide emission | g N/m^2^/d |
| 71 | $Ngas_{nit}$ | Nitrogen gas emission in nitrification process | g N/m^2^/d |
| 72 | $Ngas_{denit}$ | Nitrogen gas emission in denitrification process | g N/m^2^/d |
| 73 | $n_{imm}$ | Potential N immobilization estimated by the tentative decomposition procedure | g N/m^2^ |
| 74 | $P_{clay}$ | Percentage of clay content | % |
| 75 | *PET* | Potential evaporation | mm |
| 76 | $PP$ | Daylength | Hour |
| 77 | *pKGR* | Potential kernel growth rate | g |
| 78 | $pKW$ | Potential kernel weight | g |
| 79 | $ppsen$ | Photoperiod sensitivity coefficient | Unitless |
| 80 | $ppfun$ | Effects of photoperiod on crop development rate | Unitless |
| 81 | $q_{1}$ | Water flow rate in layer *l* | mm/s |
| 82 | $R_{a}$ | Extraterrestrial solar radiation | MJ m^-2^ d^-1^ |
| 83 | $Rh$ | Soil respiration rate | g C/m^2^/d |
| 84 | $SDate$ | Simulated sowing dates | Day of year |
| 85 | $SDate_{earliest}$ | Earliest sowing dates | Day of year |
| 86 | $SDate_{latest}$ | Latest sowing dates | Day of year |
| 87 | $T_{avg}$; $T_{max}$ | Average and maximum air temperature | ℃ |
| 88 | $T_{avg}^{10d}$; $T_{min}^{10d}$ | Actual 10-day running average and minimum air temperatures | ℃ |
| 89 | $T_{avg}^{p}$; $T_{min}^{p}$ | Thresholds of the 10-day running average and minimum temperatures | ℃ |
| 90 | $T_{soil}$ | Soil temperature | ℃ |
| 91 | $tempfun$ | Effects of temperature on crop development rate | Unitless |
| 92 | $Ver_{min}$;  ;$Ver_{max}$ | Minimum, lower optimal, upper optimal, and maximum air temperatures required for vernalization | ℃ |
| 93 | $VERDAY$ | Vernalization day | Days |
| 94 | $VDSAT$ | Vernalization days needed to saturate the vernalization response | Days |
| 95 | $verfun$ | Effects of vernalization on crop development rate | Unitless |
| 96 | $vsen$ | Vernalization sensitivity coefficient | Unitless |
| 97 | $wfp$ | Fraction of soil porosity that is filled with water | Unitless |
| 98 | $whc_{lit,act}$ | Actual water holding capacity of surface litter | mm |
| 99 | $whc_{lit,max}$ | Maximum water holding capacity of surface litter | mm |
| 100 | $X_{l}$ | Amount of SOM/nutrients in layer *l* after mixing | g C/m^2^ or g N/m^2^ |
| 101 | $X_{ol}$ | Original amount of SOM/nutrients in layer *l* before mixing | g C/m^2^ or g N/m^2^ |
| 102 | $Z$ | Depth to the bottom of the tilled layer | cm |
| 103 | $\beta$ | Soil matric potential-related factor | Unitless |
| 104 | $\Psi$ | Soil matric potential | mm |
| 105 | $\psi$ | Water potential of soil layer | mm |
| 106 | $\psi_{max}$ | Maximum leave water potential | mm |
| 107 | $\psi_{open}$; $\psi_{close}$ | Water potential under which the stomata fully opens and closes | mm |
| 108 | $\theta$; $\theta_{sat}$; $\theta_{fc}$ | Actual soil moisture content, soil moisture content at saturation, and soil moisture content at field capacity | mm |
| 109 | $\theta_{sat,pri}$; $\theta_{fc,pri}$ | Original soil moisture content at saturation and field capacity before tillage | mm |
| 110 | $\theta_{sat,aft}$; $\theta_{fc,aft}$ | Modified soil moisture content at saturation and field capacity after tillage | mm |
